# UNLOCKING MULTI-SAMPLE DIFFERENTIAL EXPRESSION FOR SPATIAL TRANSCRIPTOMICS DATA WITH TESSERA

**DOI:** 10.64898/2026.04.27.720955

**Published:** 2026-04-30

**Authors:** Florica Constantine, Zoltan Laszik, Sandrine Dudoit, Elizabeth Purdom

**Affiliations:** 1 Department of Statistics, University of California, Berkeley; 2 Department of Pathology, University of California, San Francisco; 3 Department of Statistics and Division of Biostatistics, School of Public Health, University of California, Berkeley

**Keywords:** Spatial transcriptomics, Differential expression, Spatial statistics, Multi-sample analysis, Generalized linear spatial model, Generalized linear mixed model, Scalable inference

## Abstract

Spatial transcriptomics allows the unprecedented examination of gene expression levels at the resolution of spatially-situated single cells in a high-throughput manner. As the technology is adopted more broadly, studies frequently collect data from multiple tissue samples, which leads to unique challenges that traditional spatial statistical methods are not equipped to handle. In particular, factors that differ across samples, such as different coordinate systems, different numbers and types of cells, different underlying tissue architectures, among others, preclude the application of traditional methods to our new setting. In this work, we propose a novel method, TESSERA, based on a spatial generalized linear model, for analyzing multi-sample spatial transcriptomics count data. Importantly, we provide a mathematical and computational framework for efficient and scalable model fitting and statistical inference to accompany the specification of our model. Our method for fitting the model enables the estimation of a common set of fixed effects across samples. This allows us to address a variety of differential expression questions, such as identification of which genes are differentially expressed between conditions (e.g., diseases, treatments), while accounting for spatial correlation between cells within a sample. We benchmark our proposed method on simulated data and apply it to a spatial transcriptomics dataset of human kidney samples. We find that our method provides a hitherto nonexistent extension to the multi-sample setting while remaining competitive with or outperforming existing algorithms in the single-sample setting.

## 1. Introduction.

### 1.1. Biological Background.

Spatial transcriptomics allows for the extraction of mRNA expression measurements at cellular or near-cellular resolution while simultaneously recording the physical coordinates for cell locations within a tissue (Williams et al., 2022; Ståhl et al., 2016; Rodriques et al., 2019; Vickovic et al., 2019). Spatial transcriptomics datasets typically contain hundreds to tens of thousands of cells or “spots” (i.e., groups of approximately 1 to 10 cells (10x Genomics, 2025a)). The data are highly sparse, with the majority of gene–cell or gene–spot entries equal to zero. This sparsity arises from a combination of true biological absence of expression and technical dropout, where only a fraction of the total transcripts present are successfully captured during data collection (Moses and Pachter, 2022; Williams et al., 2022; Zhao et al., 2022). Prior to the introduction of spatial transcriptomics technology, practitioners had been using single-cell RNA sequencing (scRNA-seq) to obtain gene expression measurements at the resolution of individual cells. However, scRNA-seq cannot be applied *in situ* as the tissue is dissociated to allow for the extraction of mRNA from each cell, which results in the loss of spatial information regarding cell locations within the tissue. By contrast, spatial transcriptomics technology operates at a cellular or near-cellular resolution by retaining a cell’s or spot’s spatial location in a tissue. There are two main categories of technologies, next-generation sequencing and imaging. Next-generation sequencing technologies capture mRNA molecules using barcodes along a grid of known spatial locations (Vickovic et al., 2019; Ståhl et al., 2016; Rodriques et al., 2019), while imaging-based methods detect mRNA molecules through the generation of fluorescence images from the barcoding of fluorescent probes via *in situ* sequencing or hybridization (Rao et al., 2021; Lee et al., 2014; Chen et al., 2015). The retention of the spatial positioning of the cells in the tissue enables identification of neighborhoods/neighboring cells, which allows us to better study cell behavior, as a cell’s function and associated cell-cell interactions are often highly location-dependent (Dries et al., 2021; Pareja-Lorente and Aloy, 2025; Joost et al., 2016; Hunter et al., 2021).

### 1.2. Motivation: Multiple Samples and Differential Expression.

**Figure 1:**
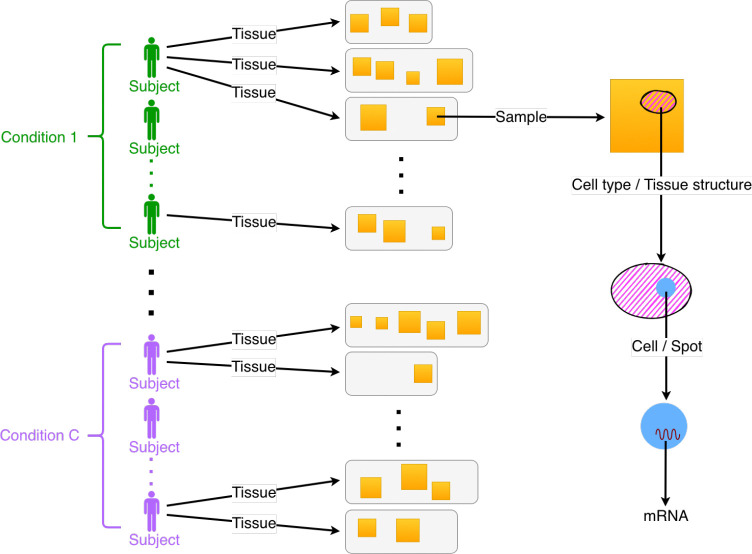
Experimental design and nomenclature. We present a general multi-sample spatial transcriptomics experimental design and associated nomenclature for discussing said design.

Researchers will often use mRNA expression measurements to identify differentially expressed genes between populations (e.g., patients with different responses to treatment, wild-type and mutant mice) in order to better understand biological processes, disease mechanisms, or the effects of treatments. A gene that exhibits distributional differences in mRNA abundance measures between groups is referred to as differentially expressed (DE). In a typical setting for bulk RNA-seq or scRNA-seq, we seek to identify differential expression for individual genes between two different cell types or states (scRNA-seq) or phenotypes (bulk RNA-seq and scRNA-seq) and can draw on a range of well-established methods that generally involve testing for differences in means (Love, Huber and Anders, 2014; Ritchie et al., 2015; Robinson, McCarthy and Smyth, 2010; Soneson and Robinson, 2018; Finak et al., 2015). However, for spatial transcriptomics, framing the question of differential expression is more complex due to the range of dimensions along which genes can be differentially expressed, including spatial location, cell type, and condition (Figure 1); i.e., there are multiple ways in which one can define differential expression, some of which may be of greater biological interest than others.

Statistical analyses for spatial transcriptomics have focused thus far on inference for a single tissue sample at a time, e.g., nnSVG (Weber et al., 2023), C-SIDE (Cable et al., 2022), SPARK-X (Zhu, Sun and Zhou, 2021), spatialDE (Svensson, Teichmann and Stegle, 2018), trendsceek (Edsgärd, Johnsson and Sandberg, 2018), among others. Rather than the conventional differential expression analysis just described, these methods address questions such as finding genes that are “spatially variable”, i.e., genes whose expression exhibits systematic variation across a given sample, forming discernible spatial patterns. Such spatial patterning might manifest as gradients of expression, localized hotspots, or regional differences that reflect underlying tissue structure, cell organization, or microenvironmental influences. Most methods also include a way to perform differential gene expression across groups, such as cell types, but only within a single tissue sample. As spatial transcriptomics technology is adopted more broadly, we can now collect data from multiple tissue samples. Existing methods do not adapt to this new setting.

Multiple samples lead to unique challenges that traditional spatial statistical methods are not equipped to handle, such as different coordinate systems that do not necessarily align, different numbers and types of cells, different tissue architectures, etc. The geospatial analog would be analysis of data from disconnected islands, with highly complex and diverse within-island spatial structure. Existing approaches would analyze each island separately, whereas we seek to use all of the data at the same time (Freni-Sterrantino, Ventrucci and Rue, 2018). In the same analogy, we would seek to discover differences in some quantity across islands, while controlling for the spatial correlation within islands. In fact, the presence of data from multiple samples or islands is a benefit, as the greater sample size yields greater statistical power and generalizability relative to data from a single sample.

The multi-sample experimental setup, besides necessitating novel statistical methodology, enables us to ask and answer a richer set of biological questions, especially with regards to DE analyses. Consider that if the goal is to find differences in expression across diseases, a single sample comes from a patient with a single disease, and hence cannot alone be used to infer a disease effect. With multiple specimens, and hence multiple samples from different groups, we may now ask if there are differences in expression across diseases or phenotypes, while controlling for spatial correlation of measurements within the samples. Were we to apply a model designed for a single sample to our multi-sample setting, we would be restricted to estimating a set of fixed effects for each sample; in contrast, the question of interest calls for the estimation of a single set of fixed effects that are shared across all samples. In the example of spatial transcriptomics, an appropriately designed multi-sample experiment and corresponding model should allow the estimation of a common disease effect as well as a single, unambiguous set of cell type fixed effects.

### 1.3. Contributions and Outline.

In this paper, we propose a novel method, TESSERA (Tool for Estimating Spatial and Sample-level Effects via Regression Analysis), for analyzing multi-sample spatial transcriptomics data. Mirroring its namesake in mosaic art, TESSERA integrates data from multiple individual samples—representing discrete “tessera” pieces—to estimate a single, unified set of fixed effects across samples, thereby enabling broader biological inferences that are not achievable when analyzing samples in isolation. To this end, we extend well-established single-sample spatial models to the multi-sample setting by allowing each sample to have its own spatial correlation structure while sharing a common set of fixed effects, and we provide a mathematical and computational framework that makes this extension efficient and scalable for model fitting and statistical inference. In particular, our framework explicitly leverages the sparsity in the four spatial correlation structures we consider. The TESSERA method is also novel within spatial statistics, as it provides a hitherto nonexistent extension to the multi-sample setting, and outperforms relevant prior baselines. It has applications beyond the biological focus of this manuscript, such as in urban planning or ecology. For example, we might identify drivers of economic activity within and across multiple metropolitan areas or study species interactions across multiple disconnected geographical regions.

We provide the mathematical specification of our TESSERA method in Section 2, with a description of our notation and spatial generalized linear mixed model (GLMM) in Section 2.1, an overview of covariance structures in Section 2.2, an outline of our model fitting method in Section 2.3, a framework for performing hypothesis testing in Section 2.4, and an overview of prior work in Section 2.5. We present a simulation study designed to validate the TESSERA method in Section 3, including power results in Section 3.4. We report on an application of the TESSERA method to a human kidney spatial gene expression dataset in Section 4. Details about data processing, design matrices, and computation are given in Section S1, and a longer discussion of the covariance structures is in Section S2. Detailed derivations and algorithmic specifications are given in Section S3. Theorems characterizing model identifiability are presented in Section S4, with proofs in Section S5.

The TESSERA method is implemented in an R package of the same name and available under the Artistic License 2.0. The package source code and documentation are currently available at https://github.com/floricaconstantine/TESSERA, and the code to reproduce the results in the manuscript is provided at https://github.com/floricaconstantine/TESSERA_manuscript. The package will be submitted to Bioconductor (https://www.bioconductor.org), the primary open-source repository for computational biology software, following publication.

## 2. The TESSERA Method.

### 2.1. Model Specification.

Consider the setting wherein we have n spatially-resolved samples indexed by i=1,2,…,n. For example, we might have disconnected samples from several tissue specimens from multiple subjects as seen in Figure 1. Within the samples, we have multiple measurements of a quantity of interest and their corresponding local (i.e., specific to the sample) spatial coordinates. In the example of samples from tissue specimens, we have numerous cells per sample, identified to spatial coordinates via the position of their centroids, and measurements of gene expression associated to each cell. To accompany each measurement, we might have values of covariates that can include cell-level or sample-level biological factors, such as sample condition, tissue structure, or cell type, as well as technical factors, such as the specific microarray slide or batch in which the cells were processed. We focus on modeling counts taken at the centroids, i.e., gene expression measures, as a function of these covariates. We want to model the counts with the primary goal of estimating fixed effects, shared across samples, for covariates of interest. However, unlike standard regression models used for non-spatial count data, such as scRNA-seq data, we have spatially-resolved count measurements and must account for the spatial correlation in our model.

In what follows, we will develop theory and methods for a single gene (or a single outcome or dependent variable in a general setting, of which spatial transcriptomics is a special case) at a time and will not use an index for genes to simplify notation. Let $Z_{i,j}$ denote the count for location (cell) j in sample i, where there are $J_{i}$ locations in sample i. We model the measured counts as

(1) $$Z_{i,j}|\theta_{i,j}\sim Pois\left(\theta_{i,j}C_{i,j}\right),$$

where the $Z_{i,j}$ are assumed to be independent across samples (for different indices i) but not necessarily across locations within a sample (for different indices j and the same index i). Here, $\theta_{i,j}$ is an unobserved random variable that provides a location-specific mean which depends on the covariate fixed effects as well as a random effect that encapsulates spatial dependence. $C_{i,j}$ is a known location-dependent scaling factor, which may be a random variable, e.g., the library size (i.e., total read count across all genes for cell j in sample i), in which case we condition on $C_{i,j}$ for the distribution of $Z_{i,j}$. Without loss of generality, one can set $C_{i,j}=1$, if scaling is not needed for a given problem.

Applying a logarithmic link, we define $\eta_{i,j}=log\;\theta_{i,j}$ and let

(2) $$\eta_{i,j}=X_{i,j}^{⊤}\beta +\phi_{i,j},$$

where $X_{i,j}\in R^{p}$ denotes known covariates to adjust for for location j in sample $i,\;\beta \in R^{p}$ is a vector of regression coefficients (the fixed effects) to be estimated, and $\phi_{i,j}$ is an unobserved spatially-varying random effect (Cressie, 2015, Section 7.5.2, p. 544–548). This is a very natural and well-known strategy for modeling count data with spatial correlation in the context of a single spatial sample (Stoehr and Robin, 2026; Murakami and Matsui, 2022; Chiquet, Mariadassou and Robin, 2021; Wang and Kockelman, 2013; Leroux, Lei and Breslow, 2000; Clayton and Kaldor, 1987). Importantly, the vector β, our parameter of interest, has no index i, as it is shared across all samples. Additionally, we note that the random effects, $\phi_{i,j}$, while accounting for spatial dependence, also add over-dispersion between samples and cells beyond that of the Poisson model, similar to the negative binomial distribution for count data.

We model the vector $\phi_{i}\in R^{J_{i}}$, consisting of $\phi_{i,j},\;j=1,\ldots ,J_{i}$, for sample i, as a multivariate normal random vector $\phi_{i}\sim 𝒩\left(0,\Sigma_{i}\right)$, with zero mean and a covariance matrix $\Sigma_{i}\in R^{J_{i}\times J_{i}}$ that captures spatial dependence between the locations. We assume that the $\phi_{i,j}$ are independent of the $X_{i,j}$. Thus, we can write the model, which we call the TESSERA model, as

(3) $$\begin{array}{c} Z_{i,j}|\theta_{i,j}\sim Pois\left(\theta_{i,j}C_{i,j}\right),\\ \eta_{i}=log\;\theta_{i}|X_{i}\sim 𝒩\left(X_{i}\beta ,\Sigma_{i}\right), \end{array}$$

where $\eta_{i}\in R^{J_{i}}$ is the collection of $\eta_{i,j},\;j=1,\ldots ,J_{i}$, for sample i, and $X_{i}\in R^{J_{i}\times p}$ is the design/covariate matrix for sample i, with the j th row set to $X_{i,j}^{⊤}$. The matrix $\Sigma_{i}$ defines how a given measurement’s nearest neighbors’ values affect its own value. For example, we might expect nearby cells in a tissue sample to influence each others’ gene expression.

We use ν to denote the entire set of model parameters for TESSERA, including the shared regression parameter β and the sample-specific parameters $\psi_{i}$ for the covariance matrices $\Sigma_{i}$ of the random effects $\phi_{i}$, that is, $\nu =\left(\beta ,\psi_{1},\ldots ,\psi_{n}\right)$. Section 2.2 below introduces two classes of models for the covariance structure.

The TESSERA method goes beyond the single-sample setting and enables modeling in the multi-sample setting. Importantly, we jointly model multiple samples with a single set of shared fixed effects β but a different covariance structures $\Sigma_{i}$ for each sample. The TESSERA method is also easily adaptable to many realistic settings and allows for statistical hypothesis testing concerning biological parameters of interest. The design matrices $X_{i}$ can incorporate multiple covariates to account for complex study designs and, by testing contrasts of the elements of β, we can perform DE analyses with respect to any quantity that can be encoded into the design matrices $X_{i}$.

### 2.2. Models for Covariance Structure.

The distribution of the spatially-varying random effects $\phi_{i}$ is governed by their covariance matrices $\Sigma_{i}$. For the implementation of the TESSERA method, we assume the $\Sigma_{i}$ come from one of two classes of models for the covariance structure: Lattice-based models and sparse nearest-neighbor Gaussian process (spNNGP) models (Datta et al., 2016; Vecchia, 1988). Each of these have been proposed previously, only in the context of a single spatial sample. We show in Section S4 that, under relatively mild conditions, the multi-sample TESSERA model of Equation (3), coupled with any of the covariance models described below, results in parameters β that are identifiable.

In what follows, we briefly describe the two different classes of covariance models and how they are parameterized. We discuss the parameters involved for a single Σ, with the understanding that for TESSERA, these parameters are defined separately for each sample, resulting in distinct $\Sigma_{i}$ for each sample (i.e., the subscript i is dropped to simplify notation). The models are discussed in greater detail in Section S2.

Both the lattice and spNNGP models specify a precision matrix $P=\Sigma^{-1}\in R^{J\times J}$, describing aspects of the spatial relationship between the observations in a given sample, but differ in how P is parameterized (Table 1). We denote by ψ the (typically unknown) parameters for P and, when relevant, use the longer notation P(ψ) to indicate the dependence of the precision matrix on these parameters.

#### 2.2.1. Lattice-Based Models.

For the lattice models, the precision matrix P is parameterized in terms of a scale parameter $\tau^{2}$, a parameter γ controlling the strength of the correlation between neighboring observations, and a user-defined (i.e., assumed to be known) adjacency matrix W specifying the neighbors of each observation. Thus, $\psi =\left(\tau^{2},\gamma \right)$ and the precision matrix may be written as $P(\psi )=P^{*}(\gamma )/\tau^{2}$, where the matrix $P^{*}$ is also a function of the known W. We consider three lattice models: Simultaneous autoregressive (SAR) and conditional autoregressive (CAR) models (Besag, 1974), and a modification of the CAR model known as the Leroux model (Leroux, Lei and Breslow, 2000). For the CAR and SAR models, γ∈(-1,1), and for the Leroux model, γ∈[0,1), where we exclude γ=±1 to ensure that P is positive-definite. The exact role of γ differs between the models. In the CAR and SAR models, γ quantifies the spatial dependence between neighboring observations. In the Leroux model, the random effect is modeled in terms of both a spatially-correlated component as well as a non-spatially-correlated component, so that γ defines a tradeoff between i.i.d. observations at γ=0 and an improper CAR model (i.e., CAR with γ=1) at γ=1. In all models, γ controls the strength of the correlation between neighboring observations. Unlike the Leroux models, the CAR and SAR models cannot represent i.i.d. data for arbitrary W; setting γ=0 leads to independent but not identically distributed measurements. Importantly, as each location or cell has only a handful of direct neighbors, the matrix P is extremely sparse. This sparsity enables scalable fitting and inference for our model. In application to spatial transcriptomics, W could be defined by determining neighboring cells or, if the cell boundaries are not available, by thresholding the distances between cell centroids. While the SAR model allows an asymmetric W, both the CAR and Leroux models require W to be symmetric; thus we will assume a symmetric W in our applications. We also require that W have no isolated points to ensure that P is invertible in all models, though not strictly required for Leroux.

#### 2.2.2. Sparse Nearest-Neighbor Gaussian Process Models.

A Gaussian process model for Σ is of the form K(ψ), for some kernel matrix K that is parameterized by ψ. For J locations in a sample, K(ψ) is a J×J matrix, where the $\left(j,j^{\prime }\right)$ entry is a function of the locations of the measurements with indices j and $j^{\prime }$. Common choices for the kernel function include the exponential, Gaussian, and Matérn kernel functions; these functions have the advantage of isotropy, wherein they only depend on the distance between pairs of locations, as opposed to the locations themselves (Rasmussen and Williams, 2006, Chapter 4). We use the Matérn kernel as the default for TESSERA. Along with spatial variance (partial sill), range, and nugget parameters, this kernel includes a smoothness parameter that provides the flexibility to represent a wide range of spatial processes. A smoothness parameter of 0.5 yields the exponential kernel and the Gaussian kernel is recovered in the infinite limit.

While Gaussian process models offer extreme flexibility in capturing spatial structure, they also lead to computational challenges. In particular, the need to consider all pairs of locations leads to a dense matrix for Σ. The authors in Vecchia (1988) noted that considering only the k nearest neighbors for each location yields a sufficiently accurate approximation to Σ for a user-specified k as small as 10–20. This suggests using a sparse nearest-neighbor Gaussian process, which assumes $\Sigma =P^{-1}$, where P is a sparse version of K(ψ), with entries of K(ψ) set to 0 outside of the k nearest neighbors (Datta et al., 2016). This choice enables scalable fitting and inference for our model, as in the lattice model setting. In spatial transcriptomics, the distances between cell centroids would be provided, K(ψ) would be calculated, and the sparse version P would be based on a user-specified parameter k for the number of neighbors to consider.

### 2.3. Fitting the TESSERA Model.

Fitting the multi-sample TESSERA model necessitates novel estimation and algorithmic procedures. A natural framework for solving this problem is maximum likelihood estimation. However, a direct maximization of the likelihood function with respect to ν would involve a high-dimensional integral over the $\phi_{i}$ and would not scale well. A Monte Carlo approach could approximate the integral, but would also require large amounts of sampling and computation. Instead, the TESSERA method is based on the expectation-conditional-maximization (ECM) algorithm, which is an iterative algorithmic framework for estimating parameters where the data-generating model depends on unobserved latent variables (Meng and Rubin, 1993).

Briefly, the ECM fitting procedure modifies the maximization step in the expectation-maximization (EM) algorithm to alternate between maximizing over different portions of the parameter vector ν separately, resulting in the following steps for the TESSERA model.

- **Expectation (E) step.** Compute the expected value of the joint log-likelihood L(ν;Z,η) of the observed counts Z and unobserved variables η, conditional on the observed counts Z, the covariates X, and current estimates ${\hat{\nu }}^{\left(k\right)}$ of the unknown parameters ν,
(4) $$Q(\nu |{\hat{\nu }}^{\left(k\right)})=E[L(\nu ;Z,\eta )|Z,X,{\hat{\nu }}^{\left(k\right)}].$$
- **Conditional maximization (CM) step.** Sequentially maximize $Q(\nu |{\hat{\nu }}^{\left(k\right)})$ with respect to ν for a single element (or subset of elements) of ν at a time (Dempster, Laird and Rubin, 1977; Meng and Rubin, 1993).

Generally, the E-step simplifies to calculating $\hat{\eta }=E[\eta |Z,X]$ or other moments of η∣Z,X, and the ‘conditioning’ here results in sequentially maximizing over one parameter from ν while holding the other parameters in the optimization constant. ECM is useful when a global maximization of $Q(\nu |{\hat{\nu }}^{\left(k\right)})$ is slow, but conditional updates can be done rapidly (or analytically). The results of Meng and Rubin (1993) show that maximizing the conditional likelihood is an approximation for the standard M-step.

Recall that for TESSERA the set of model parameters ν are the shared regression parameter β and the sample-specific parameters $\psi_{i}$ for the covariance structure of the random effects $\phi_{i}$. Due to the intractable log-likelihood, we use a Gaussian approximation proposed by Clayton and Kaldor (1987) for the distribution of η∣Z,X in the E-step (see Section S3). For the CM-step, we fix β and sequentially optimize in each sample’s spatial correlation parameters $\psi_{i}$, then optimize in β while holding the sample-specific correlation parameters fixed; this is iterated until convergence of $Q(\nu |{\hat{\nu }}^{\left(k\right)})$. EM algorithms are commonly used for single-sample spatial models (Clayton and Kaldor, 1987). The conditional maximization step of TESSERA, where each sample’s spatial correlation parameters are optimized separately per sample, results in an algorithm that is similar structurally to an EM algorithm per sample, but with the added optimization of the shared β parameter globally over all of the samples. The details of the TESSERA fitting algorithm are presented in Section S3 (see pseudocode in Algorithm 1).

The TESSERA algorithm can work with arbitrary covariance structures, but we focus on the lattice and spNNGP models, both of which have a sparse precision matrix P. This sparsity enables an efficient, scalable implementation of the ECM framework. Relative to a method based on Markov chain Monte Carlo (MCMC) that must generate thousands of posterior draws, TESSERA is able to achieve faster computational runtimes (Supplementary Figure S-1), with the caveat that, unlike exact MCMC methods, an ECM algorithm only guarantees convergence to a local optimum of the log-likelihood. Moreover, while approximations like the integrated nested Laplace approximation (INLA) can replace MCMC samplers and offer dramatic speedups at minimal loss of accuracy, INLA scales exponentially in the number of hyper-parameters, in this case, the spatial covariance parameters (Rue, Martino and Chopin, 2009; Opitz, 2017). The ECM framework, on the other hand, maintains a linear scaling in the number of parameters. Hence, the TESSERA method, through its ECM fitting procedure, can directly model count data while scaling to large numbers of samples and measurements, all with low computational overhead and fast time-to-results.


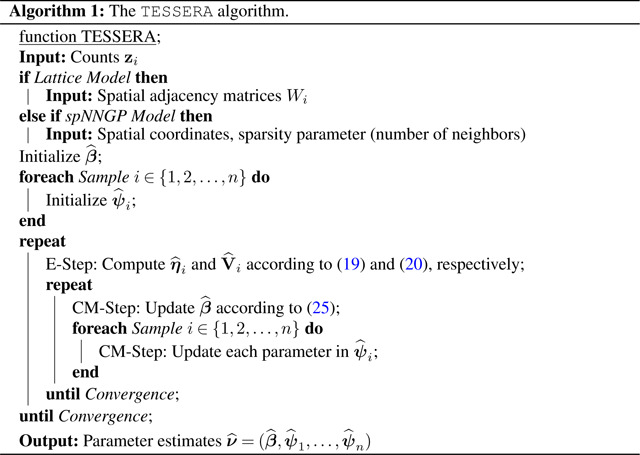


### 2.4. Hypothesis Testing.

Given an appropriate design matrix, we can use the fitted $\hat{\beta }$ from TESSERA to perform differential expression analyses, where the precise parameterization of β depends on the scientific question. We distinguish between two primary classes of covariates: within-sample (observation-level) covariates, which vary across cells or spots (e.g., cell type or tissue structure), and across-sample (sample-level) covariates, which remain constant within an individual sample but vary across samples (e.g., disease condition or treatment group). Testing the associated elements of $\hat{\beta }$ allows us to address different types of DE questions. We may identify genes differentially expressed between cell types or tissue structures, or test for interaction effects with across-sample covariates like disease condition. By accounting for these interactions, we can detect differences specific to a given cell type or structure, thereby revealing how disease effects vary across different tissue contexts. More generally, we can perform hypothesis testing for any covariate that can be encoded in a design matrix by testing relevant contrasts of the elements of $\hat{\beta }$. Details for the design matrices and associated β parameterizations used in our simulated and real data analyses (Sections 3 and 4) are provided in Section S1.2.

To perform statistical hypothesis testing for the differential expression questions we consider, we may invoke the asymptotic normality of the maximum likelihood estimator, even in the case of dependent data as in spatial transcriptomics—see Crowder (1976) and Lehmann and Casella (2006, Section 6.7, p480–481). Indeed, the likelihood function for the TESSERA model herein is smooth and sufficiently regular. Using the fact that the maximum likelihood estimator is asymptotically normal, with mean equal to the true underlying parameter value and covariance equal to the inverse of the Fisher information, we may specify a Wald test statistic for contrasts of the parameters, e.g., of the fixed effects β and of the spatial correlation parameters $\gamma_{i}$. Indeed, consider a general parameter vector $\xi \in R^{d},\;q$ linear contrasts encoded in a matrix $C\in R^{q\times d}$, and a vector of constants $c\in R^{q}$. Define the null hypothesis $H_{0}$ and the alternative hypothesis $H_{1}$ as follows

$$\left\{\begin{array}{l} H_{0}:C\xi =c\\ H_{1}:C\xi \neq c \end{array}\right..$$

Given the maximum likelihood estimator $\hat{\xi }$ of ξ, with Fisher information V and observed information $\hat{V}$, the Wald test statistic is defined as

(5) $$T=(C\hat{\xi }-c{)}^{⊤}{\left[C{\hat{V}}^{-1}C^{⊤}\right]}^{-1}(C\hat{\xi }-c).$$

Classically, under appropriate assumptions and regularity conditions, the Wald test statistic T converges in distribution to a $\chi_{q}^{2}$ random variable (Wald, 1943). However, the asymptotics of Wald statistics are poorly understood for generalized linear mixed models, and the standard approaches of degree-of-freedom estimation are not applicable to our ECM procedure (Stroup, 2013, 2015; Bell and Grunwald, 2011). Moreover, in an ECM procedure, it is unclear how to obtain standard errors. Hence, we propose the following Wald test procedure, inspired by the empirical null distribution estimation procedure from Efron (2004).

From the Hessian matrix of the expected log-likelihood, we may obtain estimated standard errors for $\hat{\beta }$ (Meilijson, 1989). Given these standard errors, we may form Wald test statistics in the usual manner, as in Equation (5). We might then take inspiration from the methodology in the limma package (Smyth, 2004) and note that, while we do not know the exact distribution of the Wald statistics and the asymptotics of the Wald statistics are measured in terms of the number of samples as opposed to the number of cells/locations, it is plausible that the Wald statistic for a single contrast behaves like a scaled $\chi_{1}^{2}$ random variable. Moreover, for finite samples, the bias in the Hessian and in the estimator of β might be non-trivial; allowing for a non-centrality parameter could account for this bias. The problem is that we do not know, a priori, what the scale and non-centrality parameters are of this scaled non-central $\chi_{1}^{2}$ distribution. If we had some threshold below which the set of Wald statistics was comprised mostly of null cases, we could use it to estimate the $\chi_{1}^{2}$ parameters.

We propose the following scheme to automatically choose the threshold. Under the null hypothesis, the p-value for a test statistic with a continuous density function should have a uniform distribution on [0, 1], 𝒰(0,1). For a given contrast, let $T_{g}$ denote the Wald test statistic for gene g=1,2,…,G. Assume that the $T_{g}$ corresponding to true null hypotheses follow a scaled non-central chi-squared distribution, $a\chi_{1}^{2}(c)$. To try to isolate those test statistics, for a given threshold value t, define the set $𝒯(t)=\left\{T_{g}:T_{g}\leq t\right\}$ from which to estimate a and c. However, since we are conditioning on $T_{g}$ being less than t, we will need to estimate a and c for a $a\chi_{1}^{2}(c)$ distribution conditioned to be less than t. We estimate the parameters by numerically maximizing the conditional likelihood in a and c.

To determine the best choice of cutoff t, we compute the p-values $p_{g}(t)$ of the $T_{g}\in 𝒯(t)$ assuming their null distribution is $\hat{a}(t)\chi_{1}^{2}(\hat{c}(t))$, and compare these p-values to a 𝒰(0,1) distribution. Let $p_{g}(t)=P\left(X\geq T_{g}\right)$, for $X\sim \hat{a}(t)\chi_{1}^{2}(\hat{c}(t))$, where $\hat{a}(t)$ and $\hat{c}(t)$ are estimates of the scale and non-centrality parameters. Given the p-values $p_{g}(t)$, compute the squared $L^{2}$ error between their empirical CDF and the CDF of the uniform distribution on [0, 1]:

(6) $$\epsilon \left(t\right)=\int_{0}^{1}{\left(u-\frac{1}{\left|𝒯\left(t\right)\right|}\sum_{\left\{g:T_{g}\in 𝒯\left(t\right)\right\}}I\left(p_{g}\left(t\right)\leq u\right)\right)}^{2}du,$$

where I is the indicator function. Hence, for each threshold t, we obtain an error ϵ(t); we choose the threshold t that minimizes ϵ(t).

As in other genomic settings, and as for virtually all differential expression methods, probability statements related to p-values rely on hard-to-verify assumptions and often do not account for prior preprocessing steps. We therefore refrain from attaching strong probabilistic interpretations to p-values and use these mostly as useful numerical summaries to determine which genes exhibit differential expression. Empirical evidence for Type I error control and power can be obtained from simulation studies or by reference to known biology, as in Sections 3 and 4 below.

### 2.5. Comparison with Prior Work.

While spatial count models, such as Poisson models with spatially-varying random effects and non-spatially-varying fixed effects, have been studied extensively (Glaser, 2017; Dass, Lim and Maiti, 2010), existing models can only handle a single sample at a time. Here, we have multiple disconnected samples with no common coordinate system and want to estimate a single set of fixed effects across samples, while estimating the spatial random effects within each sample. In this section, using our notation, we describe the evolution of non-spatial to spatial models as well as the extension of single-sample to multi-sample settings, and describe the limitations of existing methods.

#### 2.5.1. Non-Spatial Models.

The simplest applicable model for our data is a non-spatial generalized linear model based on a Poisson distribution applied to a single sample. In our notation, we would have observations $Z_{i,j}$ where

$$Z_{i,j}\overset{\;\text{indep.}\;}{\sim }\;\text{Pois}\;\left(\theta_{i,j}C_{i,j}\right),$$

and

(7) $$\eta_{i,j}=\log\theta_{i,j}=X_{i,j}^{⊤}\beta .$$

Note that this model can apply to a single sample, in which case we would drop the sample index i. Importantly, this model treats all cells or measurements as independent, even if they come from the same sample. Thus, if applied to multiple samples, this model considers all the cells to be thousands of independent observations.

An alternative model would be to consider a normal distribution, instead of a Poisson. Though we observe counts $Z_{i,j}$, we might consider logarithmically transforming them as $log\left(c+Z_{i,j}\right)$, for some constant c, typically 1 or 1/2 (Booeshaghi and Pachter, 2021; Lun, 2018). Then, the GLM for the normal distribution is equivalent to a simple linear model,

(8) $$log\left(c+Z_{i,j}\right)=X_{i,j}^{⊤}\beta +\phi_{i,j},$$

where $\phi_{i,j}\overset{\text{i.i.d.}}{\sim }𝒩\left(0,\sigma^{2}\right)$ for some variance parameter $\sigma^{2}>0$. It should be noted that the log-transformed counts are not truly continuous and treating them as such does not capture the essence of the data. In particular, in spatial transcriptomics, the observed data are quite sparse and, for many genes, the values $Z_{i,j}$ range mostly between 0 and 3. This will be a concern for all methods discussed here that use Gaussian models on log-transformed counts, rather than modeling the data with a count distribution.

Though not typically considered as a spatial model, a generalized additive model (GAM) can account for spatial information using functions of the spatial coordinates $\left(x_{j},y_{j}\right)$ of the measurements as covariates (Wood, 2017). Models of this form have been considered for spatial transcriptomics data in Cable et al. (2022). A common form for these functions is a smoothing spline, e.g., a thin-plate spline, and these models can be fit via maximum likelihood or restricted maximum likelihood approaches. It is also possible to fit a model with a per-sample spatial spline component and a common set of fixed effects via the mgcv package (Wood, 2017).

#### 2.5.2. Single-Sample Spatial Models.

As already mentioned, our TESSERA model is a generalization of models described previously in the literature for a single spatial sample, which account for spatial dependency between locations by adding a random effect to the Poisson GLM of (7),

(9) $$\eta_{j}=log\;\theta_{j}=X_{j}^{⊤}\beta +\phi_{j},$$

where $\phi_{j}$ are potentially correlated zero-mean Gaussian random variables. The CAR, SAR, Leroux, and spNNGP models correspond to different ways to describe the correlation structure of the $\phi_{j}$.

Other methods have instead extended the Gaussian model of Equation (8) by assigning a spatial correlation structure to the $\phi_{j}$. Of particular interest in the Gaussian setting is the BRISC method (Saha and Datta, 2018), which assumes continuous observations and applies a spNNGP structure to $\phi_{j}$ of Equation (8). The algorithm in BRISC builds on recent work on sparsification via nearest-neighbor approximations to accelerate the fitting of Gaussian processes. As a point of interest, the nnSVG method proposed for spatial transcriptomics uses the BRISC algorithm to test a single sample for the presence of spatial patterning, i.e., testing for the existence of spatial dependencies (Weber et al., 2023).

#### 2.5.3. Block-Wise Spatial Models.

Another class of single-sample methods supports disjoint or disconnected partitions of the sample, wherein each partition corresponds to a distinct connected component. This results in block covariance matrices, with the blocks corresponding to the different partitions. This is similar to our setting, where separate samples correspond to disjoint partitions. Indeed, the TESSERA model could be equivalently written with random effects having a block covariance matrix, with blocks defined by $\Sigma_{i}$.

One such model is the hierarchical spatial autoregressive (HSAR) model from Dong and Harris (2015), which builds on the Gaussian model in (8) with a SAR covariance structure for $\phi_{i,j}$, but allows for a block-wise structure of the SAR adjacency matrix, i.e., an adjacency matrix W that is not fully connected. However, the SAR parameters are shared across all samples, that is, there is only a single $\tau^{2}$ and γ. If we expect the spatial structure to change across samples, this model is clearly not the right fit for our data. Estimation of HSAR is accomplished via MCMC, though, at the time of writing, the relevant HSAR package is no longer in the R CRAN package repository. It should be noted, also, that HSAR assumes a Gaussian distribution, thus must be applied to the log-counts, with the ensuing problems mentioned above.

#### 2.5.4. Multivariate Models.

Another related model is the *multivariate* CAR (MCAR) model from Jin, Carlin and Banerjee (2005). This model assumes a single set of spatial locations indexed by j and allows for multivariate data $Z_{j}\in R^{K}$ to be collected at each j, e.g., expression measures for different genes for cell j. This gives a model of the form

(10) $$\eta_{j,k}=X_{j,k}^{⊤}\beta_{k}+\phi_{j,k},$$

where the counts $Z_{j,k}$ are generated according to a Poisson distribution with parameter $\theta_{j,k}=exp\;\eta_{j,k}$. Here, each outcome k has a corresponding covariate matrix $X_{k}$ and its own fixed effect vector $\beta_{k}$. The covariance structure of the random effects $\phi_{j,k}$ allows for dependence both spatially for each outcome and across outcomes, with acommon CAR structure for each outcome. The CAR structure has a correlation parameter γ that is shared across outcomes, but the scale parameter $\tau_{k}^{2}$ can vary between outcomes, as can the strength of correlation between pairs of outcomes.

In principle, this model has similar components to our TESSERA model, if we let the outcomes k be instead samples k. However, the very differing contexts for the two models result in different structural constraints: First, we wish to estimate a single set of fixed effects whereas the MCAR $\beta_{k}$ vary across samples/outcomes; second, we do not want to restrict ourselves to the setting of identical spatial locations across samples; and third, we want to allow for the values of the correlation parameter to vary across samples.

#### 2.5.5. Model Fitting.

In the single-sample setting, MCMC methods for fitting spatial Poisson models have been used successfully for small datasets (e.g., a few hundred measurements) (Zhang, 2002; Christensen, 2004). However, these methods often have problems for larger datasets, including slow mixing times due to correlation of the observations (here, the counts $Z_{i,j}$), expensive calculations and evaluations of the complicated likelihood function, and the need to repeatedly sample a high-dimensional vector of random effects (Guan and Haran, 2018, 2019). Beyond the issues with MCMC, many of these models involve complex covariance structures, e.g., Matérn or Gaussian covariances, for the spatial random effects, and for large numbers of measurements (cells, in this case), these covariance structures lead to large dense matrices and high computational demands (Sun, Zhu and Zhou, 2020; Zhu, Sun and Zhou, 2021; Dupont, Wood and Augustin, 2022; Anderson et al., 2022). While sparse approximations as in BRISC (Saha and Datta, 2018) help, they do not solve the underlying issues of the MCMC: speed and slow mixing. Moreover, off-the-shelf implementations of MCMC methods for the Poisson spatial model are relatively few, and a non-trivial degree of expertise is required to implement them from scratch—the same goes for alternatives such as Laplace approximations like INLA (integrated nested Laplace approximation) (Rue, Martino and Chopin, 2009). The INLA approximation replaces the MCMC sampler and is generally faster; examples of INLA for single-sample spatial models include the work in Vicente et al. (2023). Of particular note is the fact that, unlike existing off-the-shelf MCMC implementations in R, the R-INLA implementation can be used to specify and fit a multi-sample Poisson spatial model with distinct spatial correlation structures for each sample. However, as the timing results in Supplementary Figure S-1 indicate, this procedure is not scalable to realistically sized spatial transcriptomics datasets. For INLA, each additional sample requires estimating additional hyper-parameters (spatial covariance parameters) but the computational scaling is exponential in the number of hyper-parameters (Rue, Martino and Chopin, 2009; Opitz, 2017). Simpler than both MCMC and INLA is the EM algorithm applied to a single sample in Clayton and Kaldor (1987). At the cost of some convergence guarantees (global vs. local optima) and a slight loss of accuracy, the ECM algorithm offers simplicity and speed (see Supplementary Figure S-1).

## 3. Simulations Studies.

### 3.1. Synthetic Data Based on Real Data.

To evaluate the performance of TESSERA, we conduct a series of simulation studies based on the model defined in Equation (3). We generate data using two distinct spatial covariance models, Leroux and spNNGP, to assess the method across different types of spatial dependence. To ensure biological realism, we choose the parameters ν for these simulations by applying TESSERA to the kidney spatial transcriptomics dataset described in Section 4 and retain the original spatial coordinates and covariates (cell type and disease condition). Within a given simulation study, the datasets are generated independently across trials from the specified Poisson-Leroux or Poisson-spNNGP distribution for the $Z_{i,j}$, conditioned on these fixed parameters. We benchmark TESSERA against a Poisson and a negative binomial (NB) GLM, as well as a generalized additive model. Where applicable, we also compare our method to pseudobulk methods DESeq2 and limma-voom, which aggregate expression measures within each sample and cell type (Lun and Marioni, 2017). We assess performance in terms of accuracy for estimating model parameters, model fit, power to detect differential expression, and robustness to model misspecification.

### 3.2. Performance of TESSERA on Multi-Sample Data.

We focus on two genes, the ones with the highest and lowest variances of the raw counts across all cells and samples, thereby representing two extremes of the data. For each gene and data-generating scenario, we conduct 100 simulation trials and, for each trial, apply every method to be evaluated.

#### 3.2.1. Estimation Accuracy.

**Figure 2:**
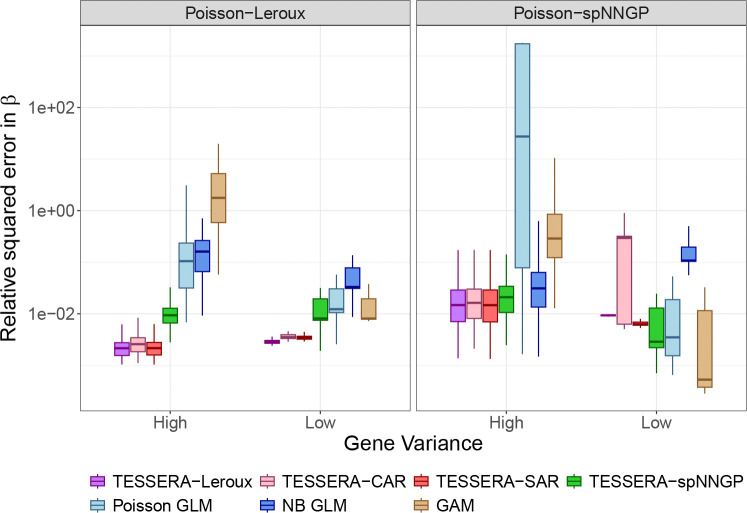
Simulation study: Relative error in estimating the shared regression coefficient β. Shown are boxplots of the relative squared error in estimating $\beta ,\|\beta -\hat{\beta }{\|}_{2}^{2}/\|\beta {\|}_{2}^{2}$, across 100 trials of simulated data. Different colored boxplots represent different methods for estimating the parameter β: TESSERA with different choices of spatial covariance structure, Poisson GLM, negative binomial GLM, and GAM. We generate synthetic data based on the kidney dataset from Abedini et al. (2024), by sampling from both the multi-sample Poisson-Leroux (left) and Poisson-spNNGP (right) distributions with parameters derived from fitting the model to the original, real data. We show results for two sets of parameters, those corresponding to the genes with the maximum (“High”) and minimum (“Low”) variances of the raw counts across all cells and samples.

When data are generated from a Poisson-Leroux distribution, the results demonstrate that TESSERA applied using this model consistently outperforms the other methods in estimating the fixed effects β in terms of the relative squared error, as shown in the left panel of Figure 2. TESSERA also produces accurate estimates of the parameters γ and $\tau^{2}$ for the spatial covariance matrix. We see that the error in γ∈[0,1) is generally low (Supplementary Figure S-2). For $\tau^{2}$, the absolute error is low for genes with true values near 0 (Supplementary Figure S-3). The relative error is consistent across genes and reflects a slight bias toward underestimating $\tau^{2}$ (Supplementary Figure S-4).

Notably, even when the model is misspecified, with data generated from a Poisson-spNNGP model, TESSERA (applied with a Poisson-Leroux model) still performs better than other methods for high-variance genes, as seen in the right panel of Figure 2. For low-variance genes, TESSERA maintains low relative squared error in β, indicating the method’s robustness even in the presence of model misspecification. For genes with variances falling between the extremes, the results are comparable, as demonstrated in Supplementary Figure S-5.

#### 3.2.2. Capturing Spatial Autocorrelation.

To compare methods in terms of their ability to account for spatial autocorrelation, we compute the Moran’s I (see Definition 4) of their residuals and contrast it to the Moran’s I of residuals for data generated using an independent baseline model (with γ=0). Use of the independent baseline allows us to differentiate between capturing genuine spatial structure and potential artifacts of model complexity. Across both the Poisson-Leroux and Poisson-spNNGP data-generating scenarios, TESSERA fit with lattice-based models consistently outperforms TESSERA fit with a spNNGP model and the non-spatial methods, by more effectively accounting for spatial autocorrelation. While other models frequently leave behind residual autocorrelation or show high variability in performance, the lattice-based models yield residuals that more closely align with spatial independence regardless of the underlying generative process (Supplementary Figures S-6 and S-7).

#### 3.2.3. Model Fit.

TESSERA fit with the multi-sample lattice models provides the best fitted counts in terms of mean squared error (MSE), with errors an order of magnitude smaller than those from the GLM and the GAM; TESSERA fit with the multi-sample spNNGP model shows errors typically falling in between the two (Supplementary Figure S-8). This indicates that explicitly modeling spatial dependence within samples can substantially improve model fit. Mirroring the analysis in Section 3.2.2, the benefit of explicitly accounting for spatial dependence is made even more apparent when we examine the difference in normalized MSE for spatially correlated data relative to the independent (γ=0) baseline. While the Poisson GLM, NB GLM, and GAM exhibit inconsistent and often substantial shifts in MSE when spatial dependence is introduced, indicating that the fit of non-spatial models is highly sensitive to gene-specific spatial patterns, TESSERA variants maintain a comparatively stable error profile. Across all genes and data-generating scenarios, TESSERA shows at most relatively small levels of increased MSE in response to the more complex setting of spatial dependence (Supplementary Figure S-9).

#### 3.2.4. Recommended Default Covariance Model for TESSERA.

In summary, these results reinforce that the TESSERA method, particularly with lattice-based models, not only provides accurate estimates of fixed effects but also excels in minimizing residuals and accounting for spatial autocorrelation, outperforming conventional methods like GLM and GAM. In what follows, for analyses where we only fit TESSERA for a single model, we use the Poisson-Leroux lattice model as our default; its covariance structure incorporates both spatial and non-spatial components—unlike the Poisson-CAR and Poisson-SAR models—and its spatial parameters are identifiable, in contrast to the Poisson-spNNGP model.

### 3.3. Performance of TESSERA on Single-Sample Data.

Although TESSERA is designed for a multi-sample setting, we validate the method on single-sample data to benchmark it against existing single-sample spatial methods. For this purpose, we generate data using the estimated parameters from a representative sample, *HK3035_ST*, within the kidney dataset (Abedini et al., 2024). We again focus on the two genes with the highest and lowest variances of the raw counts across all cells and samples in the entire kidney dataset. We conduct 100 simulation trials for each combination of gene and data-generating scenario and, for each trial, apply every method to be evaluated. We assess model performance by examining the relative squared error for the fixed effects β within a single-sample framework in Supplementary Figure S-10. TESSERA fit with lattice models consistently demonstrates estimation performance comparable to the MCMC and INLA implementations that are commonly used in spatial statistics. This indicates that TESSERA‘s computational efficiency does not come at the cost of accuracy. TESSERA fit with the Poisson-spNNGP model exhibits superior performance relative to BRISC, though the BRISC algorithm fits a Gaussian model to log-transformed counts and thus has a different underlying model. Notably, non-spatial Poisson and NB GLM exhibit significantly higher error rates, by several orders of magnitude, highlighting the necessity of accounting for spatial autocorrelation.

In the aforementioned analysis, we dropped four cells from the *HK3035_ST* sample, corresponding to two cell types: one with three cells and another with only one cell. While the corresponding entries of β are technically estimable, leaving these cells in the dataset might lead to worse results. To examine the effect of these small cell groups or of high variance elements in $\hat{\beta }$, we present the relative squared error of the fixed effects when we do not drop these four cells in Supplementary Figure S-11. The MCMC results show a substantial degradation in performance, characterized by much larger variances and error values. This pattern also appears when using INLA for genes with low variance. In contrast, the ECM-based TESSERA results remain stable, leading us to conclude that TESSERA is robust to the presence of these small subgroups.

### 3.4. TESSERA Power and Type I Error Control.

#### 3.4.1. Simulation Model.

The power simulations remain grounded in the kidney spatial transcriptomics dataset (Abedini et al., 2024), where the elements of β correspond to interactions between cell type and disease condition and to sample-specific main effects. For these simulations, however, we focus on a single gene and modify the specification of its β vector to concentrate on the detection of differences across disease conditions for a given cell type. Concretely, we begin with the $\hat{\beta }$ from either a Poisson-Leroux or Poisson-spNNGP model fit to a high-variance gene (*IGHG2*). For a given cell type, we set the coefficient corresponding to the control condition to the average of all coefficients in $\hat{\beta }$. We then vary the coefficient corresponding to the diabetic kidney disease (DKD) condition by adding a value in [−2, 2] to the value of the control coefficient to create an effect size for the contrast. We do this separately for each of four cell types, with on average approximately 40, 75, 165, and 600 cells per sample, to cover a wide range of realistic scenarios (see Supplementary Figure S-12 for the range of numbers of cells per sample). To evaluate power, we perform 100 simulation trials for each combination of contrast value and data-generating scenario and, for each trial, apply every method to be evaluated. Power is then calculated as the proportion of trials in which we reject the null hypothesis that the control-cell type and DKD-cell type interaction coefficients are equal.

To test for differential expression with TESSERA, we perform Wald tests following the procedure in Section 2.4. The parameters of the scaled non-central chi-squared null distribution are estimated using Wald statistics derived from the real data. Specifically, we fit a Poisson-Leroux model to all 3,000 genes and compute Wald statistics for all within-cell-type, across-condition contrasts. This collection of (3,000 genes × 3 condition pairs × 26 cell types) statistics yields a $a\chi_{1}^{2}(c)$ null distribution, which is then used to compute p-values for the Wald statistics generated in our power simulations. As detailed in Section S3.2, the covariance of $\hat{\beta }$ is used to compute these Wald statistics.

We benchmark the performance of the TESSERA method against the GLM and GAM alternatives as well as pseudobulk methods DESeq2 and limma-voom. To accommodate the multi-gene dispersion estimation required by pseudobulk methods, we fit their models using all available genes but replace a single gene with synthetic data generated by modifying $\hat{\beta }$ as described above.

While these simulations focus on a single gene, practical applications typically involve simultaneous testing across thousands of genes, bringing additional considerations such as multiple testing adjustment. Additionally, the parameters of the $a\chi_{1}^{2}(c)$ null distribution were estimated once based on a single realization of the data (specifically, that of the real data), thus missing in the simulations the variability in that estimation process. To capture this variability, these parameters would have to be re-estimated across a full set of simulated genes for every realization of the data, which is computationally prohibitive.

#### 3.4.2. Characterizing the Power and Type I Error Rate Trade-off.

**Figure 3:**
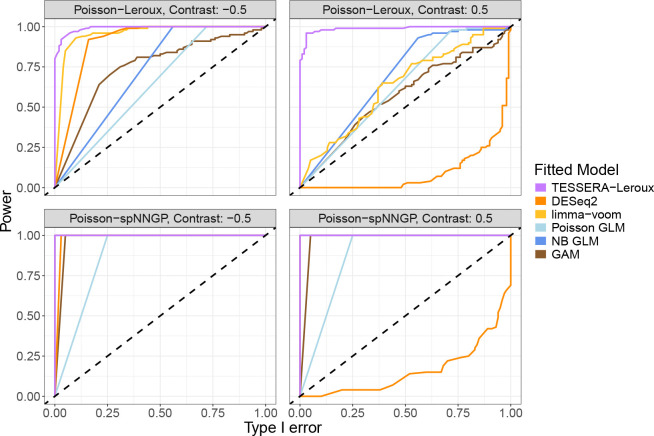
Simulation study: ROC curves. Receiver operating characteristic curves illustrate the trade-off between power (y-axis) and Type I error rate (x-axis) for detecting DE between conditions within a cell type for one gene. The dotted diagonal line represents the y=x identity line, where the power is exactly equal to the Type I error rate. Curves that rise more steeply toward the top-left corner indicate superior method performance. Different colored lines correspond to different methods, as indicated in the legend. Synthetic data are generated by sampling from either a multi-sample Poisson-Leroux (top row) or Poisson-spNNGP (bottom row) distribution with parameters derived from fitting the model to the *IGHG2* gene in the kidney dataset from (Abedini et al., 2024), except for entries of β which are modified to create the contrast values represented in the individual plot headers (see details in Section 3.4). Results are displayed for the *C_TAL* cell type and the DKD vs. Control condition comparison.

We now consider the relative performance of TESSERA in detecting differentially expressed genes. We generated receiver operating characteristic (ROC) curves across a broad range of effect sizes and cell types for the DKD vs. Control condition comparison. Representative results are shown in Figure 3 for the *C_TAL* cell type (~600 cells per sample) for two effect sizes; ROC curves for a wider range of values are provided in Supplementary Figures S-13 and S-14. The ROC curves illustrate that TESSERA consistently outperforms or matches the performance of other methods. Furthermore, TESSERA has consistently high performance, unlike other methods whose performance can vary greatly for different simulation models or effect sizes.

**Figure 4:**
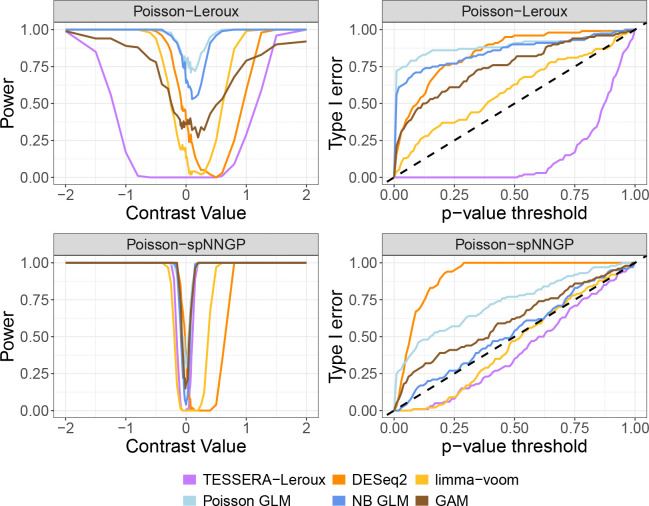
Simulation study: Power and Type I error rate. This figure examines power and Type I error for detecting DE between conditions within a cell type for one gene. In the left-hand column, we present plots of power (y-axis) as a function of the contrast value (x-axis), where power is estimated by the proportion of simulations in which the null hypothesis of no DE is rejected at nominal Type I error rate of 0.05. At an effect size of 0, this value represents the Type I error rate. In the right-hand column, we present curves of the Type I error rate (y-axis) as a function of the p-value threshold (x-axis), where exact control of the Type I error rate is represented by the dotted line y=x and methods with curves above this line do not effectively control the Type I error. Different colored lines correspond to different methods, as indicated in the legend. Synthetic data are generated by sampling from either a multi-sample Poisson-Leroux (top row) or Poisson-spNNGP (bottom row) distribution with parameters derived from fitting the model to the *IGHG2* gene in the kidney dataset from Abedini et al. (2024), except for entries of β which are modified to create the effect sizes represented on the x-axis for the power curves (see details in Section 3.4). Results are displayed for the *C_TAL* cell type and the DKD vs. Control condition comparison.

We can examine the overall behavior demonstrated in the ROC curves more closely by considering the power and Type I error explicitly (Figure 4). The right-hand column of Figure 4 shows the observed Type I error curve in simulation for different p-value thresholds. TESSERA is the only method that controls the Type I error across both settings. In contrast, the curves for all other methods (except limma-voom in the spNNGP setting) lie above the 45-degree line, indicating that they inflate the Type I error and produce more false positives than the reported nominal level.

Turning to the power analysis, the curve shown in the left-hand column of Figure 4 shows that all methods exhibit the expected V-shaped power curve, where power increases with the magnitude of the effect size (see Supplementary Figures S-15 and S-16 for additional cell types). Of particular note is a distinct rightward shift in the power curves of all methods other than TESSERA; while this is observed for all comparison methods in the Leroux setting, it persists for pseudobulk methods limma and DESeq2 across both settings. This shift also helps explain the lack of Type I error control for pseudobulk methods. Notably, when the effect size is zero, the plotted power value represents the empirical Type I error rate, and typically this is also the minimum of the power curve. The pseudobulk methods exhibit curves whose minimum power values are similar to TESSERA, but are shifted to the right. Because of this shift, these minima are not located at the point of zero effect size, but at a point where the methods should have power to detect an effect. This shift is also reflected in the behavior of the pseudobulk methods in the ROC curves of Figure 3, which dip below the 45-degree diagonal at small positive contrast values where the Type I error rate exceeds the power to detect the true signal. In both covariance settings, TESSERA is the only approach that achieves its power while strictly maintaining the Type I error rate at a nominal level of 0.05.

Beyond the differences between methods, we also observed a discrepancy in absolute power between the two covariance settings. While TESSERA has lower power in the Leroux setting than in the misspecified spNNGP setting, this difference is driven by the magnitude of the standard errors of the estimated contrasts. As seen in Figure S-17, the standard errors of the estimated contrasts in the spNNGP setting are less than half of those in the Leroux setting, while the estimated contrast values are similar (though we note that the spNNGP contrast estimates tend to be slightly larger in magnitude). The smaller standard errors lead to notably larger Wald statistics, and hence likely explain the higher power for the spNNGP setting relative to the Leroux setting.

#### 3.4.3. Impact of Cell Abundance.

The extent of Type I error inflation is strongly influenced by cell abundance. We examined performance across four cell types ranging from ~40 to ~600 average cells per sample. For the GAM, Poisson GLM, and NB GLM methods, the deviation from the nominal Type I error rate becomes progressively more severe as the number of cells per sample increases (Supplementary Figures S-18 and S-19). In the case of the Poisson and NB GLM, GAM, and DESeq2, the Wald statistic is modeled as having a $\chi_{1}^{2}$ distribution, where the standard errors are estimated from the observed information matrix, which corresponds to the Hessian of a misspecified likelihood function that treats the counts as independent. Here, treating the counts as independent leads to standard errors that are systematically underestimated, as evidenced by standard errors that decrease with increasing cell abundances while the nominal Type I error simultaneously worsens (Supplementary Figures S-20 and S-21).

#### 3.4.4. Robustness via Empirical Null Distribution Estimation.

While limma-voom does not completely control the Type I error rate, its deviations are notably less pronounced than those of the other benchmarked methods. This superior performance likely stems from its structural flexibility; by employing a Gaussian likelihood, limma-voom decouples the mean and variance, whereas Poisson and NB models are constrained by rigid, predetermined mean-variance dependencies. This decoupling allows limma-voom to more accurately approximate empirical distributions that do not strictly conform to the fixed dispersion structures inherent to Poisson and NB models.

TESSERA provides a different path toward robustness by addressing the inherent limitations of relying on a theoretical null distribution. While limma-voom and DESeq2 both share information across genes to stabilize specific model parameters such as variance or dispersion, TESSERA instead shares information across genes for the test statistics themselves to characterize the null distribution. By employing a scaled non-central $\chi_{1}^{2}$ distribution to learn the empirical null distribution directly from the data, TESSERA seeks to ensure valid inference even under model misspecification. Consequently, while limma-voom provides more flexibility than NB or Poisson-based models, it remains constrained by a theoretical null distribution, a limitation shared by DESeq2 and the cell-level Poisson and NB-based GLM or GAM models. In contrast, TESSERA maintains robust Type I error control by adapting its null distribution to the unique characteristics of the observed test statistics. This empirical approach is essential for accurate Type I error control; as demonstrated in Supplementary Figures S-22 and S-23, applying a standard chi-squared null distribution with one degree of freedom fails to maintain the nominal Type I error rate.

We compared TESSERA against fdrtool (Strimmer, 2008), a widely-used implementation of the empirical null framework that builds upon the local FDR approach established by Efron (2004). Notably, TESSERA‘s estimation procedure (Section 2.4) does not require the null distribution to be centered or symmetric, providing a more versatile framework than methods like fdrtool, which rely on such assumptions. While performance on the simulated data is virtually identical (Supplementary Figures S-22, S-23, S-24, and S-25), TESSERA generalizes to settings where skewed or shifted null distributions would otherwise violate the symmetry and centering assumptions of conventional empirical models. As in our application of TESSERA‘s thresholding procedure for the null distribution, we applied fdrtool to a single realization (the real data with 3,000 genes).

#### 3.4.5. Robustness to Spatial Covariance Structure.

Interestingly, non-spatial models show higher Type I error inflation in the Leroux data-generating setting than in the spNNGP setting. The Leroux model generates local, discrete clusters of spatial dependency (Cramb et al., 2020; Wall, 2004), whereas the spNNGP model produces global, smooth gradients characteristic of the Matérn covariance family (Datta et al., 2016). The Leroux localized structure represents a more severe departure from the independence assumption, sharply reducing the effective sample size (Griffith, 2005) and making these “pockets” of expression more likely to be misinterpreted as biological signal by non-spatial models. TESSERA is less prone to spurious discoveries as it partitions the total variance into components related to the fixed effects and to a structured spatial random effect, thereby preventing random spatial autocorrelation from being misattributed to the contrast of interest.

## 4. Real Data Analysis: Visium Kidney Tissue Samples.

We analyze a spatial transcriptomics dataset generated using the Visium platform on formalin-fixed, paraffin-embedded (FFPE) human kidney tissue samples (Abedini et al., 2024). The dataset comprises 14 samples from distinct patients, representing three conditions: three healthy controls, six patients with diabetic kidney disease (DKD), and five patients with hypertensive kidney disease (HKD). Cell type annotations were provided by the original authors, using Cell2Location (Kleshchevnikov et al., 2022) and CellTrek (Wei et al., 2022) to identify 26 distinct cell types, with varying numbers of cells per cell type–disease combination (Supplementary Figure S-26). We further filtered the dataset to retain the most highly variable genes and computed library sizes to serve as normalization factors in our analyses (see Section S1.1).

For all analyses, we applied the TESSERA method to fit a multi-sample Poisson-Leroux model and compared its results with a Poisson GLM, a negative binomial GLM, a generalized additive model, and pseudobulk approaches (DESeq2 and limma-voom).

All models were fit using a fully-crossed design between cell type and disease and include a fixed effect for sample (see Section S1.2 for details). Differential expression analyses were performed both between cell types and between conditions within an individual cell type. For the TESSERA model, we obtained p-values using our Wald test thresholding procedure (Section 2.4). For each method and analysis, we computed adjusted p-values using the Benjamini-Hochberg (BH) procedure (Benjamini and Hochberg, 1995) for control of the false discovery rate (FDR) over genes.

### 4.1. Model Fit.

**Figure 5:**
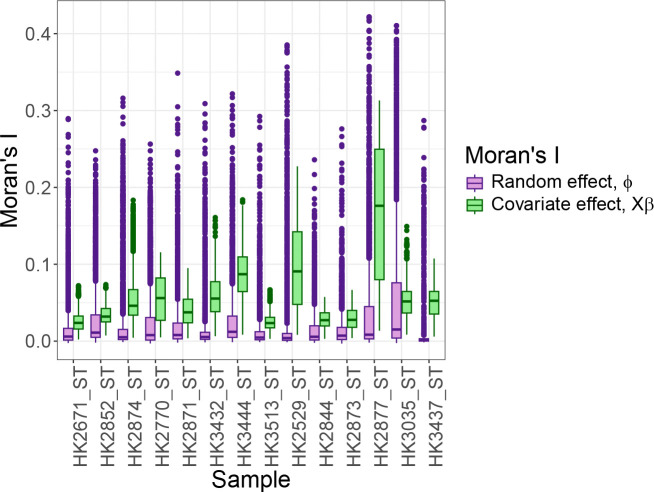
Kidney dataset: Moran’s I of spatial random effect and covariate effect. Boxplots show, per sample, the distribution across genes of Moran’s I calculated on the estimated spatial random effects $\hat{\phi }$ (purple) and covariate effects $X\hat{\beta }$ (green) (see Section 4 for details). Results are based on fitting a multi-sample Poisson-Leroux model with TESSERA to the kidney dataset (Abedini et al., 2024). The $X\hat{\beta }$ have large values of Moran’s I, demonstrating that the covariates induce significant spatial patterning, but the estimated random effects also capture additional, non-trivial spatial autocorrelation.

The results of applying TESSERA on this dataset demonstrate several important advantages of the underlying multi-sample spatial model. Firstly, in most settings, at least some cell types will be highly spatially clustered while also having quite differing gene expression levels; not including them in the model (via the design matrix in the model of Equation (3)) can result in estimates of spatial variation which are actually due to cell type distinctions (Walker et al., 2022; Yu and Luo, 2022; Gaspard-Boulinc et al., 2025; Shang, Wu and Zhou, 2025). Our model allows for the separation of cell type effects from spatial effects. The gene expression within each sample can be decomposed into estimated covariate (e.g., cell type) effects $X\hat{\beta }$ and estimated spatial random effects $\hat{\phi }=\hat{\eta }-X\hat{\beta }$, where $\hat{\eta }$ is estimated using the approximation in Equation (19) and the ECM algorithm. When we calculate the Moran’s I of $X\hat{\beta }$ compared to the Moran’s I of $\hat{\phi }$, we see high values for $X\hat{\beta }$ (Figure 5), indicating the importance of including cell type covariates to avoid spuriously categorizing cell type spatial patterning as autocorrelation. We also see that $\hat{\phi }$ retains substantial spatial autocorrelation, distinct from the notably higher signal found in the covariate component. This confirms that while the spatial distribution of cell types accounts for much of the observed spatial structure, our model successfully identifies an underlying spatial dependence.

Moreover, we find that TESSERA fit with the Poisson–Leroux model generally yields smaller residuals, as measured by the normalized mean squared error, compared with the other methods (Supplementary Figure S-27). Compared with the Poisson and NB GLM, the TESSERA residuals exhibit a substantially stronger decrease in their spatial autocorrelation, as measured by Moran’s I, compared to the original counts (Supplementary Figure S-28). Given that on synthetically generated non-spatial, over-dispersed count data the decrease in Moran’s I is comparable for TESSERA and the Poisson and NB GLM (Supplementary Figure S-29), our results here indicate that a non-spatial model does not adequately explain all the variation inherent to the data.

Beyond the need for a spatial model, we note that the estimated spatial parameters γ and $\tau^{2}$ vary widely across samples (Supplementary Figure S-30), demonstrating that there is substantial heterogeneity in the spatial correlation structure across samples necessitating separate spatial parameters per sample.

### 4.2. Spatial Modeling Recovers Localized Anatomical Structure.

To further examine the behavior of TESSERA against a non-spatial model, we focus on the gene *ACTA2*, a canonical marker for vascular smooth muscle cells (*VSMC*), in sample *HK3035_ST*. This sample contains a prominent *VSMC* cluster where *ACTA2* exhibits a notable degree of spatial structure in $\hat{\phi }$, summarized by a Moran’s I value of 0.19. This choice of gene-sample pair illustrates TESSERA‘s ability to identify a strong, spatially organized signal that is distinct from the cell type fixed effects.

**Figure 6:**
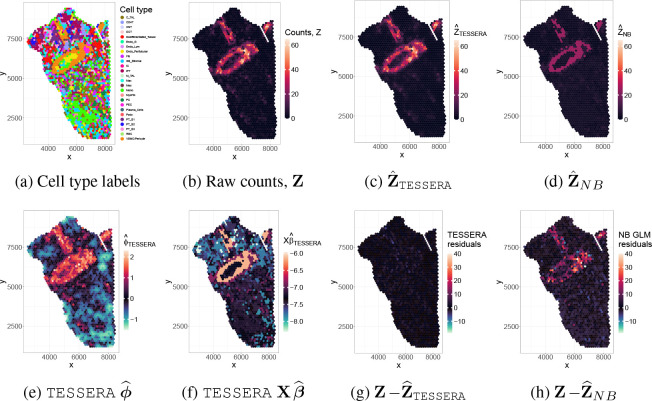
Kidney dataset: Capturing structured vascular expression. For the kidney dataset (Abedini et al., 2024), we examine the results of model fitting for both TESSERA with a Poisson-Leroux model and an NB GLM on the gene *ACTA2*. We focus on the results for sample *HK3035_ST*. **(a)** Categorical cell type annotations used as fixed effects in both models. **(b)** Observed raw counts, Z, for the vascular marker *ACTA2*. **(c)** TESSERA fitted counts, ${\overset{ˆ}{Z}}_{\text{TESSERA}}$. (d) NB GLM fitted counts, ${\overset{ˆ}{Z}}_{NB}$. **(e)** TESSERA estimated spatial random effects, $\hat{\phi }$. **(f)** TESSERA estimated covariate (cell type) effects, $X\hat{\beta }$. **(g)** TESSERA raw residuals, $Z-{\hat{Z}}_{\text{TESSERA}}$. **(h)** NB GLM raw residuals, $Z-{\hat{Z}}_{\text{NB}}$.

The TESSERA method provides a superior fit for the vascular smooth muscle marker *ACTA2* for this sample compared to a non-spatial NB GLM. Both models include fixed effects for cell types (Figure 6a) to establish a baseline for gene expression; however, while the NB GLM produces residuals that cluster heavily along a renal artery (Figure 6h), TESSERA effectively reduces this structured error through a Poisson-Leroux spatial random effect $\hat{\phi }$ (Figures 6g and 6e). The spatial patterns in the NB GLM residuals stem from the assumption of spatial invariance, which forces a single, uniform average on every spot with the same cell type label regardless of its anatomical context. Because this cell type-specific average is calculated globally, it cannot capture the high-intensity signals of major vessels, causing the NB GLM fitted values (${\hat{Z}}_{NB}$, Figure 6d) to appear fragmented and systematically underestimated compared to the raw counts (Figure 6b). Although the NB GLM uses an over-dispersion parameter to account for this remaining variance, it treats the variation as random rather than structured, leaving the model unable to resolve the biological signal along the tissue’s architecture. By incorporating both a covariate effect $X\hat{\beta }$ (Figure 6f) and Poisson-Leroux spatial random effect $\hat{\phi }$ (Figures 6e), TESSERA more effectively approximates the architecture of the kidney’s arterial network in the final fitted counts (${\hat{Z}}_{TESSERA}$, Figure 6c). Consequently, the TESSERA fitted counts are closer to the observed raw counts and are more biologically consistent with the known physical architecture of the kidney.

### 4.3. Differential Expression Between Cell Types.

Next, we examine whether applying TESSERA to identify genes that are DE between cell types yields known marker genes that are predominantly expressed in specific cell types and serve to characterize them or distinguish between them and other cell types. We use the kidney marker genes outlined in Lake et al. (2023, Supp. Table 5) mapped to the cell types present in Abedini et al. (2024) (Supplementary Figure S-31) and focus on the comparison of proximal tubule segment 1 (*PT_S1*) cells and cortical thick ascending limb (*C_TAL*) cells. The marker genes are specific to their respective designated cell type (Supplementary Figure S-32), making this a reasonable comparison.

Formally, we consider the following contrast

$$\frac{1}{3}\left(\beta_{C_{-}TAL,\;\mathrm{Control}\;}+\beta_{C_{-}TAL,DKD}+\beta_{C_{-}TAL,HKD}\right)-\frac{1}{3}\left(\beta_{PT_{-}S1,\;\mathrm{Control}\;}+\beta_{PT_{-}S1,DKD}+\beta_{PT_{-}S1,HKD}\right).$$

We test for DE between the two cell types using the Wald test of Section 2.4, where the parameters of the null distribution are estimated using all genes across all pairwise cell type contrasts (i.e., based on 3,000×262 test statistics), and begin by ranking genes according to their Wald test statistics (Supplementary Figure S-33). We can see that all methods rank the marker genes relatively similarly and with a generally low rank, a strong indication of differential expression, as we would expect.

We next create volcano plots for each method (Supplementary Figure S-34), where we see that the marker genes have higher estimated effects and lower BH adjusted p-values than non-marker genes across all methods. If we then compare the number of genes that were found to be significantly DE (at a nominal FDR level of 0.05), we find that TESSERA fit with the multi-sample Poisson-Leroux model detects similar numbers of genes as the pseudobulk methods and less than half the number of genes as found by the GLM and the GAM (Supplementary Figure S-35). The simulation results in Section 3.4 show that these non-spatial methods fail to control the Type I error, therefore implying that most of their discoveries are likely false positives; indeed, the data exhibit significant spatial autocorrelation, making non-spatial models prone to inflated statistical significance and hence potential false discoveries.

Lastly, we examine the overlap between methods in the ranking of all genes, not just marker genes, and observe high Jaccard similarity across methods for the top-ranked genes (Supplementary Figure S-36). This consistency is expected given the distinct functional roles of proximal tubule and cortical thick ascending limb cells. Due to the pronounced differences in gene expression between these cell types, the primary markers are easily captured by all methods, leading to a high degree of consensus in the resulting rankings.

### 4.4. Differential Expression Between Conditions, Within Cell Types.

We next study the more biologically interesting scenario of differential expression between conditions within a cell type, where, for cell type c, the contrasts of interest are

$$\beta_{c,\;DKD}-\beta_{c,\;Control},\;\beta_{c,\;HKD}-\beta_{c,\;Control},\;\beta_{c,\;HKD}-\beta_{c,\;DKD}.$$

We examine the number of genes found to be DE (at a nominal FDR level of 0.05) by our TESSERA method, using the procedure described in Section 2.4, where the parameters of the null distribution are estimated using the Wald statistics from all within-cell type pairwise contrasts between conditions (Control, DKD, and HKD) for all genes (i.e., 3,000 × 3 × 26 test statistics). Diagnostics of the Wald test statistics for the TESSERA method indicate that our thresholding procedure identifies a single optimal threshold that minimizes the $L^{2}$ error. Applying the resulting threshold produces a p-value distribution for sub-threshold statistics that closely follows a 𝒰(0,1) distribution (Supplementary Figure S-37), with the corresponding scale and shift parameters for these test statistics shown in Supplementary Figure S-38.

**Figure 7:**
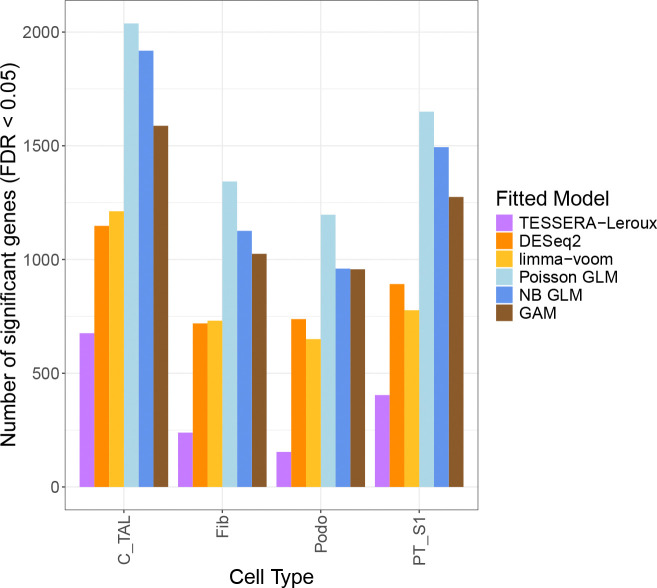
Kidney dataset: Number of differentially expressed genes for the Control vs. DKD contrast. The height of each bar indicates the number of genes found to be differentially expressed between the Control and DKD conditions in four cell types representing different structured compartments of the kidney, for the dataset of Abedini et al. (2024): Cortical thick ascending limb (*C_TAL*), fibroblasts (*Fib*), podocytes (*Podo*), and proximal tubule segment 1 (*PT_S1*). For all methods, significance is based on Benjamini-Hochberg-adjusted p-values with a nominal FDR level of 0.05. For TESSERA, p-values are derived from the Wald test procedure described in Section 2.4. Different colors correspond to the results from the different DE methods (see legend).

We compare the number of differentially expressed genes identified by TESSERA to those found by Poisson GLM, NB GLM, GAM, and pseudobulk methods limma-voom (Ritchie et al., 2015) and DESeq2 (Love, Huber and Anders, 2014). All DE methods are consistent in that many more genes are identified as DE between DKD and the other two conditions than between Control and HKD, as seen in Supplementary Figure S-39. This result is consistent with previous work and with the underlying pathology of DKD and HKD (Barrios et al., 2025). However, the number of genes found DE for a particular comparison varies considerably from method to method as seen in Figure 7 and Supplementary Figure S-40.

Specifically, in Figure 7, TESSERA identifies the fewest DE genes among the methods compared, followed by the pseudobulk approaches. Notably, the GAM and GLM methods identify more than twice as many DE genes as TESSERA, a finding consistent with the results seen in the *C_TAL* vs. *PT_S1* comparison (Supplementary Figure S-35). This suggests that accounting for spatial correlation in our model reduces our effective sample size appropriately and, taken together with the results on Type I error in Section 3.4, demonstrates that accounting for spatial correlation leads to fewer spurious discoveries. Furthermore, TESSERA exhibits higher Jaccard similarity for top-ranked genes with pseudobulk methods, which treat the sample as the experimental unit, than with GLM or GAM methods, which treat cells as independent observations (Supplementary Figure S-41).

## 5. Discussion.

In this paper, motivated by recent advances in spatial transcriptomics technology and its growing adoption, we have proposed the TESSERA method for analyzing large, multi-sample datasets. Key elements of our method are that it is based on a Poisson model that operates directly on raw counts, that it allows for distinct spatial covariance structures in each sample while estimating a single set of fixed effects across samples, and that it is based on a scalable ECM algorithmic framework. Additionally, we are able to present identifiability results for the fixed effects β as well as for the spatial covariance parameters in lattice models.

We have demonstrated the performance of TESSERA on realistically generated synthetic data. We showed that it accurately estimates the underlying fixed effects and the spatial covariance parameters of the lattice models, and that it accurately models the counts (measurements). Compared to non-spatial methods, our method leads to a substantial reduction in the spatial autocorrelation of the fitted residuals. Furthermore, TESSERA enables robust statistical inference, achieving superior or comparable power at any given Type I error rate across a broad range of effect sizes compared to simpler GLM and GAM alternatives and standard pseudobulk methods. Crucially, out of all compared methods, only ours was able to consistently control the Type I error rate regardless of the underlying spatial generative process. While non-spatial methods showed worsening Type I error control as cell counts increased and pseudobulk methods like limma and DESeq2 exhibited biased power curves, TESSERA provided valid inference across all settings. These results underscore the necessity of models that both account for spatial autocorrelation and accommodate heterogeneity in spatial structures across samples.

We applied our method to a real spatial transcriptomics dataset, generated by the authors in Abedini et al. (2024). We find that our method finds far fewer differentially expressed genes than all other methods, both for comparisons between cell types and comparisons between conditions for a given cell type. Taken together with the power and Type I error results on the synthetic data, we conclude that it is likely that a substantial portion of the genes discovered by other methods are false discoveries.

Within spatial transciptomics, we believe that the TESSERA method is an extremely useful analytic framework that avoids the pitfalls incurred by not accounting for the spatial correlation between neighboring cells. Notably, our approach provides a hitherto nonexistent extension to the multi-sample setting, while remaining competitive with or outperforming existing algorithms in the single-sample setting, thereby filling a critical gap in current methodological capabilities. Moreover, compared to GAMs or existing spatial computational frameworks such as MCMC samplers and INLA, our ECM framework enables faster model fitting and inference under the increasing computational demands of multi-sample settings with numerous experimental units and spatial locations. While we have focused on spatial transcriptomics applications, we expect that TESSERA has application beyond genomics, e.g., in urban planning, ecology, or for geospatial analyses involving islands or other disconnected structures.

## Supplementary Material

Supplement 1

## Figures and Tables

**Table 1:** Parameterization of the precision matrix for covariance models considered for TESSERA. For the lattice models, the precision matrix P depends on two typically unknown parameters, $\tau^{2}$ and γ, and a known cell-cell adjacency matrix W;D denotes the diagonal matrix with entries equal to the row-sums of W. For spNNGP, K is a kernel matrix that depends on some set of possibly unknown parameters ψ (e.g., number of neighbors, range, nugget, etc.) and the coordinates of the cells.

Model	Precision matrix P	Contraints on parameters

CAR	$(D-\gamma W)/\tau^{2}$	$\tau^{2}\in (0,\infty ),\gamma \in (-1,\;1),W=W^{⊤}$
SAR	${\left(I-\gamma D^{-1}W\right)}^{⊤}D\left(I-\gamma D^{-1}W\right)/\tau^{2}$	$\tau^{2}\in (0,\infty ),\gamma \in (-1,\;1)$
Leroux	$[(1-\gamma )I+\gamma (D-W)]/\tau^{2}$	$\tau^{2}\in (0,\infty ),\gamma \in [0,\;1),W=W^{⊤}$
spNNGP	K(ψ)	$K(\psi )=K(\psi {)}^{⊤}$
