# Supplementary material for: UNLOCKING MULTI-SAMPLE DIFFERENTIAL EXPRESSION FOR SPATIAL TRANSCRIPTOMICS DATA WITH TESSERA
Source: bioRxiv. 2026 Apr 30:2026.04.27.720955. Preprint. [Version 1] doi: 10.64898/2026.04.27.720955 (PMC13142504; doi:10.64898/2026.04.27.720955)
Supplement: Supplement 1 [file NIHPP2026.04.27.720955v1-supplement-1.pdf]

bioRxiv preprint doi: https://doi.org/10.64898/2026.04.27.720955; this version posted April 30, 2026. The copyright holder for this preprint (which was not certified by peer review) is the author/funder, who has granted bioRxiv a license to display the preprint in perpetuity. It is made available under aCC-BY 4.0 International license.

SUPPLEMENTARY MATERIAL

S1. Model Implementation. This section provides implementation details for the application of TESSERA and other methods to both simulated and real data in Sections 3 and 4, respectively. Section S1.1 outlines the data preprocessing steps applied to the spatial transcriptomics kidney dataset from Abedini et al. (2024). Section S1.2 describes the design matrix which encodes the fully-crossed effects of cell type and disease along with sample-specific fixed effects, and which is used for all methods. Section S1.3 then summarizes the computational settings, preprocessing steps, and software used for both single-sample and multi-sample analyses. Table 2 provides a complete overview of the models, input data, and key algorithmic parameters.

S1.1. *Data Preprocessing.* The dataset from Abedini et al. (2024) was preprocessed by the authors to remove low-quality spots and low-expression genes, resulting in 12,511 genes measured across 37,143 cells. To account for variation in sequencing depth across cells, we computed a library size for each cell as the sum of its counts across all genes. This quantity, $C_{i,j}$, serves as a standard proxy for sequencing depth and was included as a normalization factor in all downstream models. Following common practice in high-dimensional gene expression analyses, to reduce noise and improve power, we retained only the most variable genes. Inspection of the variance–rank curve for $\log_{10}$-transformed gene variances (Supplementary Figure S-42) revealed a clear inflection point at approximately 3,000 genes, and we therefore restricted our analyses to this subset of genes.

The spNNGP model depends on pairwise distances between cells and the lattice models on cell-cell adjacency matrices. For the spNNGP model, pairwise Euclidean distance matrices are calculated between the cell centroids, i.e., their spatial coordinates. To derive cell-cell adjacency matrices for the lattice models, we threshold the pairwise distances between cell centroids. In particular, an analysis of these pairwise distances revealed that, for the vast majority of cells, there is a large gap between the sixth-smallest and seventh-smallest distance values. This gap in distances corresponds to the hexagonal pattern in the 10x Genomics Visium platform, so that we expect all cells that are not on the edge of the tissue to have exactly six neighbors (Crowell et al., 2025; 10x Genomics, 2025b). Were we given data from an imaging platform (e.g., CosMx SMI), we would instead use the results of the cell segmentation on the tissue images and define neighboring cells as those whose minimum distance between boundaries is under some threshold or whose boundaries are in contact.

S1.2. *Model Design Matrix.* For both the real and synthetic data analyses, we use a fully-crossed design between cell type and disease for all models. The design includes no intercept and no cell type and no disease main effects, but does include a fixed effect for sample. That is, the design matrix consists of binary indicators, with one column for each possible combination of cell type and disease (e.g., *C_TAL* cells in a Control sample) and one column for each sample. Concretely, our model has the form

$$\eta_{i,j} = \sum_{\text{cell types } c} \sum_{\text{diseases } d} \beta_{c,d}\, \mathbb{I}(\text{cell } (i,j) \text{ is of cell type } c)\, \mathbb{I}(\text{cell } (i,j) \text{ comes from disease } d) + \beta_i + \phi_{i,j},$$

where $\mathbb{I}$ is the indicator function. The corresponding design matrix has full column rank and no zero rows.

An equivalent model is a properly-constrained model that includes an intercept and main effects

$$\eta_{i,j} = \mu + \sum_{\text{cell types } c} \beta_c\, \mathbb{I}(\text{cell } (i,j) \text{ is of cell type } c)$$

bioRxiv preprint doi: https://doi.org/10.64898/2026.04.27.720955; this version posted April 30, 2026. The copyright holder for this preprint (which was not certified by peer review) is the author/funder, who has granted bioRxiv a license to display the preprint in perpetuity. It is made available under aCC-BY 4.0 International license.

$$+ \sum_{\text{diseases } d} \beta_d \, \mathbb{I}(\text{cell } (i,j) \text{ comes from disease } d)$$
$$+ \sum_{\text{cell types } c} \sum_{\text{diseases } d} \beta_{c,d} \, \mathbb{I}(\text{cell } (i,j) \text{ is of cell type } c) \, \mathbb{I}(\text{cell } (i,j) \text{ comes from disease } d)$$
$$+ \beta_i + \phi_{i,j}.$$

It is important to note that, for identifiability, either some constraints must be imposed on the parameters β (e.g., sum-to-zero) or the summations must not range over all levels. That is, there must be a reference level for each of the factors. To see that the two models are equivalent, note that every cell must belong to some combination of disease and cell type and both design matrices are capable of uniquely representing all such combinations. Thus the design matrices of both the intercept-and-main-effects model and the interaction-only model are simply different linear reparameterizations of one another. That is, any group mean that can be expressed as a sum of a baseline and offsets can also be expressed directly as a standalone mean. It follows that the design matrices have the same range and that the models are equivalent.

The advantage of the intercept-free model is that the contrasts of interest, differences in cell types within a disease or differences between diseases for a given cell type, are simply differences of two coefficients, whereas in the model with main effects and an intercept, the formulation is slightly more complex.

For our analysis of the kidney dataset, the column corresponding to the Endo-Lym cell type interaction with the HKD condition is dropped, as there are no Endo-Lym cells in the HKD samples.

S1.3. *Implementation Details.* We implemented a range of models to assess both spatial and non-spatial approaches in single-sample and multi-sample settings. Alongside the TESSERA method, these include standard Poisson and negative binomial GLM, generalized additive models (GAM), pseudobulk methods (DESeq2 and limma-voom), as well as spatial models including CAR, Gaussian-SAR, Leroux, and sparse NNGP.

For the Poisson and negative binomial models, raw counts were analyzed directly. For linear and Gaussian-based models, counts were log-transformed with a 0.5 offset to handle zero values. For pseudobulk methods, counts were aggregated for each cell type-sample combination. MCMC-based methods were run with four chains, 200 burn-in iterations, 2,000 total iterations, and thinning by 2. The sparse NNGP model used $k = 15$ nearest neighbors and an exponential covariance kernel.

All analyses were performed in R (version 4.5.1). Table 2 summarizes the models and methods, software functions, input data, and key algorithmic settings for both single- and multi-sample methods.

bioRxiv preprint doi: https://doi.org/10.64898/2026.04.27.720955; this version posted April 30, 2026. The copyright holder for this preprint (which was not certified by peer review) is the author/funder, who has granted bioRxiv a license to display the preprint in perpetuity. It is made available under aCC-BY 4.0 International license.

TABLE 2
Methods evaluated and implementation specifications.

Setting	Method	R Function / Package	Data Input	Algorithmic Settings
Single- and multi-sample	TESSERA	Custom implementation	Raw counts	≥30 ECM iterations; 5 CM-steps/iteration; tolerance 10^{-3}; max 200 iterations; initialization per Sec. S3.2
	Poisson GLM	`glm` (stats)	Raw counts	`family = Poisson`
	NB GLM	`glm.nb` (MASS)	Raw counts	Default NB settings
	GAM	`bam` (mgcv)	Raw counts	Poisson family; thin-plate spline basis
	Poisson-BYM2	`INLA` (`R-INLA`)	Raw counts	Default settings; per-sample correlation structure
Multi-sample	limma-voom	`voom`, `lmFit` (limma)	Pseudobulk counts	Library-size normalization
	DESeq2	`DESeq` (DESeq2)	Pseudobulk counts	Default DESeq2 pipeline
Single-sample	Linear model	`lm` (stats)	$\log(\text{counts} + 0.5)$	Standard OLS
	Poisson-CAR (MCMC)	`brm` (brms)	Raw counts	4 chains; burn-in = 200; total iterations = 2000; thinning = 2
	Gaussian-SAR (MCMC)	`brm` (brms)	$\log(\text{counts} + 0.5)$	Same MCMC settings as above
	Poisson-Leroux (MCMC)	`S.CARleroux` (CARBayes)	Raw counts	Same MCMC settings as above
	Sparse NNGP	`BRISC_estimation` (BRISC)	$\log(\text{counts} + 0.5)$	$k = 20$ nearest neighbors; Matérn kernel

S2. Covariance Model Specification. In this section, we discuss the details of the models used by TESSERA to define the covariance matrix $\mathbf{\Sigma}_i$ of observations within a given sample i (i.e., the specification of the random effect distribution). Each $\mathbf{\Sigma}_i$ is defined by a set of parameters $\boldsymbol{\psi}_i$ which are specific to each sample, i.e., not shared across samples. For notational simplicity, in the remainder of this section, we suppress the sample index i, with the understanding that all parameters introduced should be considered sample-specific.

S2.1. *Lattice Spatial Random Effect Specification: SAR, CAR, and Leroux.* For the lattice models, there are two parameters that make up $\boldsymbol{\psi}$, $\boldsymbol{\psi} = (\tau^2, \gamma)$, and we can express the precision matrix of the random effect $\boldsymbol{\phi}$ (and thus of $\boldsymbol{\eta}$) as $\mathbf{P}(\boldsymbol{\psi}) = \mathbf{P}^*(\gamma)/\tau^2$, where $\mathbf{P}^*$ depends on a known adjacency matrix and a typically unknown attraction or correlation parameter γ, with $\gamma \in (-1, 1)$ for the SAR and CAR models and $\gamma \in [0, 1)$ for the Leroux model (Table 1). We use the same notation γ in each model to emphasize the parameter's role in defining the level of spatial attraction between neighboring observations, but it should be recognized that γ has different meanings in the different models and thus values of γ from different models cannot be directly compared.

Let $\mathbf{W} \in \{0, 1\}^{J \times J}$ denote the known measurement-site (cell-cell) adjacency matrix of the J observations (cells) within a sample. That is, $W_{j,j'} = W_{j',j} = 1$ if locations j and j' are adjacent and 0 otherwise; the diagonals are also set to zero. As a technical condition, we require $\mathbf{W}$ to have no isolated points; connecting an isolated location to its nearest location solves this problem. Moreover, for the SAR and CAR models, a fully-connected adjacency matrix avoids a non-invertible precision matrix.

Define $\mathbf{D} \in \mathbb{N}^{J \times J}$ to be the diagonal degree matrix, where $D_{j,j}$ is the number of neighbors that location j has. Since $\mathbf{W}$ has no isolated points, $\mathbf{D}$ is invertible. Furthermore, a given location likely has at most a handful of neighbors (or nearby locations deemed to be neighbors), so that $\mathbf{W}$ is mostly zero. Thus, importantly, $\mathbf{W}$ and $\mathbf{D}$ can be stored and treated as sparse matrices.

bioRxiv preprint doi: https://doi.org/10.64898/2026.04.27.720955; this version posted April 30, 2026. The copyright holder for this preprint (which was not certified by peer review) is the author/funder, who has granted bioRxiv a license to display the preprint in perpetuity. It is made available under aCC-BY 4.0 International license.

S2.1.1. *SAR Model.* Analogous to an autoregressive process in time series data analysis, the spatial autoregressive (SAR) model for ϕ_j, $j = 1, \ldots, J$, is:

$$\phi_j = \frac{\gamma}{D_{j,j}} \sum_{j'=1}^{J} W_{j,j'} \phi_{j'} + \epsilon_j, \tag{11}$$

where $\epsilon_j \sim \mathcal{N}(0, \tau^2 / D_{j,j})$ (Besag and Kooperberg, 1995; Besag, 1974). It follows that the vector $\boldsymbol{\phi} \in \mathbb{R}^J$ of the ϕ_j satisfies

$$\boldsymbol{\phi} \sim \mathcal{N}\left(\mathbf{0}, \tau^2 \left(\mathbf{I} - \gamma \mathbf{D}^{-1} \mathbf{W}\right)^{-1} \mathbf{D}^{-1} \left[\left(\mathbf{I} - \gamma \mathbf{D}^{-1} \mathbf{W}\right)^{-1}\right]^{\top}\right),$$

or that the precision matrix $\mathbf{P} \in \mathbb{R}^{J \times J}$ satisfies

$$\tau^2 \mathbf{P} = \left(\mathbf{I} - \gamma \mathbf{D}^{-1} \mathbf{W}\right)^{\top} \mathbf{D} \left(\mathbf{I} - \gamma \mathbf{D}^{-1} \mathbf{W}\right). \tag{12}$$

To see this, we note that

$$\boldsymbol{\phi} = \gamma \mathbf{D}^{-1} \mathbf{W} \boldsymbol{\phi} + \boldsymbol{\epsilon},$$

so that

$$\boldsymbol{\phi} = (\mathbf{I} - \gamma \mathbf{D}^{-1} \mathbf{W})^{-1} \boldsymbol{\epsilon}.$$

For $\mathbf{P}^{-1}$ to be a proper covariance matrix, we require $\tau^2 > 0$ and $\gamma \neq \pm 1$. Importantly, due to the structure of $\mathbf{P}$ in the SAR model, $\mathbf{W}$ is not required to be symmetric for $\mathbf{P}$ to be symmetric.

S2.1.2. *CAR Model.* The conditional autoregressive (CAR) model for ϕ_j, $j = 1, \ldots, J$, is:

$$\phi_j \mid \phi_{j' \neq j} \sim \mathcal{N}\left(\frac{\gamma}{D_{j,j}} \sum_{j'=1}^{J} W_{j,j'} \phi_{j'}, \frac{\tau^2}{D_{j,j}}\right). \tag{13}$$

We recall Brook's Lemma, wherein for a random variable $\mathbf{X} \in \mathbb{R}^n$ with two sets of values $\mathbf{x}$ and $\mathbf{y}$ and density f with conditionals f_j, we have that (Brook, 1964)

$$\frac{f(\mathbf{x})}{f(\mathbf{y})} = \prod_j \frac{f_j(x_j \mid x_1, x_2, \ldots, x_{j-1}, y_{j+1}, y_{j+2}, \ldots, y_n)}{f_j(y_j \mid x_1, x_2, \ldots, x_{j-1}, y_{j+1}, y_{j+2}, \ldots, y_n)}.$$

When applying the lemma to the CAR model, take $\mathbf{x}$ to be the vector $\boldsymbol{\phi} \in \mathbb{R}^J$ of ϕ_j's and set $\mathbf{y} = \mathbf{0} \in \mathbb{R}^J$, so that

$$\begin{aligned}
\frac{f(\boldsymbol{\phi})}{f(\mathbf{0})} &= \prod_j \frac{\exp\left(-\frac{D_{j,j}}{2\tau^2}\left(\phi_j - \gamma \sum_{j'<j} \frac{W_{j,j'}}{D_{j,j}} \phi_{j'} - \gamma \sum_{j'>j} \frac{W_{j,j'}}{D_{j,j}} 0\right)^2\right)}{\exp\left(-\frac{D_{j,j}}{2\tau^2}\left(0 - \gamma \sum_{j'<j} \frac{W_{j,j'}}{D_{j,j}} \phi_{j'} - \gamma \sum_{j'>j} \frac{W_{j,j'}}{D_{j,j}} 0\right)^2\right)} \\
&= \exp\left(-\frac{1}{2\tau^2} \sum_j D_{j,j}\left(\phi_j - \gamma \sum_{j'<j} \frac{W_{j,j'}}{D_{j,j}} \phi_{j'}\right)^2 + \frac{1}{2\tau^2} \sum_j D_{j,j}\left(\gamma \sum_{j'<j} \frac{W_{j,j'}}{D_{j,j}} \phi_{j'}\right)^2\right) \\
&= \exp\left(-\frac{1}{2\tau^2} \sum_j D_{j,j} \phi_j^2 + \frac{2}{2\tau^2} \gamma \sum_{j'<j} D_{j,j} \frac{W_{j,j'}}{D_{j,j}} \phi_{j'} \phi_j\right)
\end{aligned}$$

bioRxiv preprint doi: https://doi.org/10.64898/2026.04.27.720955; this version posted April 30, 2026. The copyright holder for this preprint (which was not certified by peer review) is the author/funder, who has granted bioRxiv a license to display the preprint in perpetuity. It is made available under aCC-BY 4.0 International license.

$$= \exp\left(-\frac{1}{2\tau^2}\boldsymbol{\phi}^\top \mathbf{D}\left(\mathbf{I}-\gamma\mathbf{D}^{-1}\mathbf{W}\right)\boldsymbol{\phi}\right)$$

$$= \exp\left(-\frac{1}{2\tau^2}\boldsymbol{\phi}^\top\left(\mathbf{D}-\gamma\mathbf{W}\right)\boldsymbol{\phi}\right).$$

Noting that $f(\mathbf{0})$ is a constant (i.e., it does not depend on $\boldsymbol{\phi}$ but only on the constants $\mathbf{W}$, $\mathbf{D}$, γ, and τ^2), we have that $f(\boldsymbol{\phi}) \propto f(\boldsymbol{\phi})/f(\mathbf{0})$. It follows that, after normalizing appropriately,

$$\boldsymbol{\phi} \sim \mathcal{N}\left(\mathbf{0}, \tau^2\left(\mathbf{D}-\gamma\mathbf{W}\right)^{-1}\right),$$

or that

$$\tau^2\mathbf{P} = \mathbf{D}-\gamma\mathbf{W}. \tag{14}$$

As in the SAR model, for $\mathbf{P}^{-1}$ to be a proper covariance matrix, we require $\tau^2 > 0$ and $\gamma \neq \pm 1$. Importantly, unlike the SAR model, we require $\mathbf{W}$ to be symmetric for $\mathbf{P}$ to be symmetric in the CAR model. We note that the CAR and SAR models are related, in that it is possible to choose a $\mathbf{W}$, γ, and τ^2 for one model so that the resulting distribution is the same as that of the other, though practically we usually consider $\mathbf{W}$ as fixed and determined by the problem (Ver Hoef, Hanks and Hooten, 2018).

S2.1.3. *Leroux Model.* The Leroux model is in the same style as the CAR model, where we have (Leroux, Lei and Breslow, 2000)

$$\phi_j \mid \phi_{j'\neq j} \sim \mathcal{N}\left(\frac{\gamma}{\gamma D_{j,j}+(1-\gamma)}\sum_{j'=1}^{J} W_{j,j'}\phi_{j'}, \frac{\tau^2}{\gamma D_{j,j}+(1-\gamma)}\right). \tag{15}$$

Essentially, relative to the CAR model, the random effect is decomposed as having both a spatially-correlated component as well as a non-spatially-correlated component, wherein $\gamma \in [0,1)$ defines the tradeoff between the two: at $\gamma = 0$, we have independence between the measurements, while as $\gamma \to 1$, we recover the intrinsic CAR model (i.e., the improper CAR model with $\gamma = 1$) (Leroux, Lei and Breslow, 2000). The matrix $\mathbf{P}$ is then

$$\tau^2\mathbf{P} = \gamma\left(\mathbf{D}-\mathbf{W}\right)+(1-\gamma)\mathbf{I}, \tag{16}$$

and

$$\boldsymbol{\phi} \sim \mathcal{N}\left(\mathbf{0}, \tau^2\left[\gamma\left(\mathbf{D}-\mathbf{W}\right)+(1-\gamma)\mathbf{I}\right]^{-1}\right).$$

As in the CAR model, for $\mathbf{P}^{-1}$ to be a proper covariance matrix, we require $\tau^2 > 0$ and γ cannot be 1. Importantly, like the CAR model, we require $\mathbf{W}$ to be symmetric for $\mathbf{P}$ to be symmetric.

Each of these three models is a natural fit for settings in which neighboring cells might interact or communicate, and cells that are not adjacent are less likely to directly communicate (Song et al., 2019). That is, in a spatial transcriptomics dataset, it is entirely natural to model a cell's expression as a function of its direct neighbors, or nearby cells, and less so to (directly) include the effects from further-away cells (as a Gaussian or Matérn covariance would entail). That this selection leads to a sparse covariance structure is a boon that will enable the scalability of the model we propose.

bioRxiv preprint doi: https://doi.org/10.64898/2026.04.27.720955; this version posted April 30, 2026. The copyright holder for this preprint (which was not certified by peer review) is the author/funder, who has granted bioRxiv a license to display the preprint in perpetuity. It is made available under aCC-BY 4.0 International license.

S2.2. *spNNGP Random Effect Specification.* For a Gaussian process model, $\mathbf{\Sigma}$ would be parameterized as $\mathbf{\Sigma}(\boldsymbol{\psi}) = \mathbf{K}(\boldsymbol{\psi})$, for some kernel matrix $\mathbf{K} \in \mathbb{R}^{J \times J}$ that is parameterized by $\boldsymbol{\psi}$. Often, the structure of $\mathbf{K}$ can be expressed as

$$\mathbf{K}(\boldsymbol{\psi}) = \mathbf{P}^{-1}(\boldsymbol{\psi}) = \tau^2 \left[\gamma \widetilde{\mathbf{K}} + (1-\gamma)\mathbf{I}\right], \tag{17}$$

where $\tau^2 > 0$ is an overall measure of scale (analogous to the lattice models) and γ trades off between the spatial and non-spatial components of the covariance (exactly analogous to the Leroux model). In this formulation, $\gamma\tau^2$ and $(1-\gamma)\tau^2$ correspond to the spatial variance (partial sill) and nugget parameters, respectively. The matrix $\widetilde{\mathbf{K}}$ captures the pairwise relationship between locations. If we denote the measurement locations (coordinates) as $\mathbf{s}_j$, for isotropic kernels like we consider herein, the (j, j') entry of $\widetilde{\mathbf{K}}$ and hence $\mathbf{K}(\boldsymbol{\psi})$ are functions of $\| \mathbf{s}_j - \mathbf{s}_{j'} \|_2$. Beyond the measures of scale for the spatial and non-spatial components of the covariance, kernel functions such as the exponential, Gaussian, and Matérn include a range parameter; the Matérn kernel also includes a smoothness parameter (Rasmussen and Williams, 2006, Chapter 4).

The problem with inference for $\mathbf{P}^{-1}$ as defined here is that it is a dense matrix with a dense inverse (precision matrix). We make use of the approximation from Vecchia (1988), wherein only the nearest k neighbors are considered when forming the precision matrix $\mathbf{P}$, where values of k of around 10 to 20 are reasonable. In particular, we make use of the findings of Datta et al. (2016), wherein we may write

$$\mathbf{P} = (\mathbf{I} - \mathbf{A})^\top \mathbf{D}^{-1} (\mathbf{I} - \mathbf{A}), \tag{18}$$

where $\mathbf{A}$ is a strictly lower-triangular matrix with at most k non-zero entries per row and $\mathbf{D}$ is diagonal—we refer the interested reader to the pseudocode in Finley et al. (2019, Section 2) for the details in forming the matrix $\mathbf{A}$. Hence, we have found a sparse approximation for the precision matrix $\mathbf{P}$ and, similar to the lattice models, enabled rapid inference of the parameter $\boldsymbol{\beta}$. We might think of this class of models as related to the lattice models in that only a few neighbors per measurement are considered directly, but with the added flexibility of allowing different kernel functions to model the spatial dependencies.

S3. Fitting the TESSERA Model. Recall that $\boldsymbol{\beta}$ denotes the vector of regression coefficients shared across samples and $\boldsymbol{\psi}_i$ denotes the parameters for the sample-specific spatial covariance matrices $\mathbf{\Sigma}_i$, $i = 1, \ldots, n$; for the lattice models, $\boldsymbol{\psi}_i$ can be partitioned into the parameters τ_i^2 and γ_i. Let $\boldsymbol{\nu} = (\boldsymbol{\beta}, \boldsymbol{\psi}_1, \ldots, \boldsymbol{\psi}_n)$ be the vector of all parameters to be estimated and $\boldsymbol{\nu}_i = (\boldsymbol{\beta}, \boldsymbol{\psi}_i)$ the subset of parameters for the likelihood of sample i.

Let $\mathbf{P}_i(\boldsymbol{\psi}_i) = \mathbf{\Sigma}_i^{-1}(\boldsymbol{\psi}_i) \in \mathbb{R}^{J_i \times J_i}$ be the precision matrix for sample i. For the lattice models, one may write $\mathbf{P}_i(\boldsymbol{\psi}_i) = \mathbf{P}_i^*(\gamma_i)/\tau_i^2$, where the different versions of $\mathbf{P}_i^*(\gamma_i)$ are provided in Table 1.

S3.1. *Approximation of the Distribution of* $\boldsymbol{\eta} \mid \mathbf{Z}, \mathbf{X}$. In both estimating and testing hypothesis for the parameters $\boldsymbol{\nu}$, we rely on a normal approximation to the conditional likelihood of $\boldsymbol{\eta}_i \mid \mathbf{Z}_i, \mathbf{X}_i$, so as to make likelihood computations feasible. In this and the following sections, we draw from the development in Cressie (2015, Section 7.5.2, p. 544–548) that is based on the work in Clayton and Kaldor (1987).

Specifically, from Clayton and Kaldor (1987), we have that the distribution of $\boldsymbol{\eta}_i$ given the observed counts $\mathbf{Z}_i$ and covariates $\mathbf{X}_i$ (for a single sample) can be approximated with a normal distribution, $\boldsymbol{\eta}_i \mid \mathbf{Z}_i, \mathbf{X}_i \mathrel{\dot\sim} \mathcal{N}(\widetilde{\boldsymbol{\eta}}_i, \widetilde{\mathbf{V}}_i)$, with

$$\mathbb{E}\left[\boldsymbol{\eta}_i \mid \mathbf{Z}_i, \mathbf{X}_i\right] \approx \widetilde{\boldsymbol{\eta}}_i(\boldsymbol{\nu}_i) = \left[\mathbf{P}_i(\boldsymbol{\psi}_i) + \Delta_i\right]^{-1} \times \left[\mathbf{P}_i(\boldsymbol{\psi}_i)\,\mathbf{X}_i\,\boldsymbol{\beta} + \Delta_i\,\boldsymbol{\zeta}_i - \frac{1}{2}\mathbf{1}\right] \tag{19}$$

bioRxiv preprint doi: https://doi.org/10.64898/2026.04.27.720955; this version posted April 30, 2026. The copyright holder for this preprint (which was not certified by peer review) is the author/funder, who has granted bioRxiv a license to display the preprint in perpetuity. It is made available under aCC-BY 4.0 International license.

and

$$\text{(20)} \qquad \mathrm{Var}\left[\boldsymbol{\eta}_i \mid \mathbf{Z}_i, \mathbf{X}_i\right] \approx \widetilde{\mathbf{V}}_i(\boldsymbol{\nu}_i) = \left[\mathbf{P}_i(\boldsymbol{\psi}_i) + \Delta_i\right]^{-1} \in \mathbb{R}^{J_i \times J_i},$$

where $\mathbf{1}$ denotes the vector of all 1, Δ_i the diagonal matrix with entries $Z_{i,j} + (1/2)$, and $\boldsymbol{\zeta}_i$ the vector of $\log((Z_{i,j} + 1/2)/C_{i,j})$.

S3.2. *The Expectation-Conditional-Maximization Algorithm.* For the $(k+1)$st iteration of the expectation-conditional-maximization (ECM) algorithm, we perform the following two steps.

S3.2.1. *E-Step.* Compute the conditional expected value of the joint log-likelihood $L(\boldsymbol{\nu}; \mathbf{Z}, \boldsymbol{\eta})$ of the observed counts $\mathbf{Z}$ and the unobserved $\boldsymbol{\eta}$, given the observed counts $\mathbf{Z}$ and covariates $\mathbf{X}$

$$Q(\boldsymbol{\nu} \mid \widehat{\boldsymbol{\nu}}^{(k)}) = \mathbb{E}[L(\boldsymbol{\nu}; \mathbf{Z}, \boldsymbol{\eta}) \mid \mathbf{Z}, \mathbf{X}, \widehat{\boldsymbol{\nu}}^{(k)}].$$

For the lattice models, the joint log-likelihood (up to a constant in $\boldsymbol{\nu}$) is given by

$$L(\boldsymbol{\nu}; \mathbf{Z}, \boldsymbol{\eta}) = \textstyle\sum_{i=1}^n \left\{ -\frac{J_i}{2} \log \tau_i^2 + \frac{1}{2} \log\det \mathbf{P}_i^*(\gamma_i) - \frac{1}{2\tau_i^2} (\boldsymbol{\eta}_i - \mathbf{X}_i \boldsymbol{\beta})^\top \mathbf{P}_i^*(\gamma_i) (\boldsymbol{\eta}_i - \mathbf{X}_i \boldsymbol{\beta}) \right\},$$

and for the spNNGP model,

$$L(\boldsymbol{\nu}; \mathbf{Z}, \boldsymbol{\eta}) = \sum_{i=1}^n \left\{ \frac{1}{2} \log\det \mathbf{P}_i(\boldsymbol{\psi}_i) - \frac{1}{2} (\boldsymbol{\eta}_i - \mathbf{X}_i \boldsymbol{\beta})^\top \mathbf{P}_i(\boldsymbol{\psi}_i) (\boldsymbol{\eta}_i - \mathbf{X}_i \boldsymbol{\beta}) \right\}.$$

Note that finding $Q(\boldsymbol{\nu} \mid \widehat{\boldsymbol{\nu}}^{(k)})$ is not equivalent to simply replacing $\boldsymbol{\eta}_i$ with $\mathbb{E}[\boldsymbol{\eta}_i \mid \mathbf{Z}_i, \mathbf{X}_i]$ in the appropriate likelihood equations above, as the expected value of a quadratic form involves also the covariance matrix (cf. Equation (38)), here $\mathrm{Var}[\boldsymbol{\eta}_i \mid \mathbf{Z}_i, \mathbf{X}_i]$. The calculation of the conditional means and covariance matrices of $\boldsymbol{\eta}_i \mid \mathbf{Z}_i, \mathbf{X}_i$ is intractable. Making use of the normal approximation of the distribution of $\boldsymbol{\eta}_i \mid \mathbf{Z}_i, \mathbf{X}_i$ given in Section S3.1, with $\widetilde{\boldsymbol{\eta}}_i(\boldsymbol{\nu}_i)$ and $\widetilde{\mathbf{V}}_i(\boldsymbol{\nu}_i)$ defined in Equations (19) and (20), respectively, we estimate the function $Q(\cdot)$ with $\widetilde{Q}(\cdot)$ defined as follows. For the lattice models,

$$\begin{aligned}\widetilde{Q}(\boldsymbol{\nu} \mid \widehat{\boldsymbol{\nu}}^{(k)}) = \sum_{i=1}^n \Bigg\{ & -\frac{J_i}{2} \log \tau_i^2 + \frac{1}{2} \log\det \mathbf{P}_i^*(\gamma_i) \\ & - \frac{1}{2\tau_i^2} \left(\widetilde{\boldsymbol{\eta}}_i(\widehat{\boldsymbol{\nu}}_i^{(k)}) - \mathbf{X}_i \boldsymbol{\beta} \right)^\top \mathbf{P}_i^*(\gamma_i) \left(\widetilde{\boldsymbol{\eta}}_i(\widehat{\boldsymbol{\nu}}_i^{(k)}) - \mathbf{X}_i \boldsymbol{\beta} \right) \\ & - \frac{1}{2\tau_i^2} \mathrm{Tr}\left[\mathbf{P}_i^*(\gamma_i) \widetilde{\mathbf{V}}_i(\widehat{\boldsymbol{\nu}}_i^{(k)}) \right] \Bigg\},\end{aligned}$$

and for the spNNGP

$$\begin{aligned}\widetilde{Q}(\boldsymbol{\nu} \mid \widehat{\boldsymbol{\nu}}^{(k)}) = \sum_{i=1}^n \Bigg\{ & \frac{1}{2} \log\det \mathbf{P}_i(\boldsymbol{\psi}_i) \\ & - \frac{1}{2} \left(\widetilde{\boldsymbol{\eta}}_i(\widehat{\boldsymbol{\nu}}_i^{(k)}) - \mathbf{X}_i \boldsymbol{\beta} \right)^\top \mathbf{P}_i(\boldsymbol{\psi}_i) \left(\widetilde{\boldsymbol{\eta}}_i(\widehat{\boldsymbol{\nu}}_i^{(k)}) - \mathbf{X}_i \boldsymbol{\beta} \right) \\ & - \frac{1}{2} \mathrm{Tr}\left[\mathbf{P}_i(\boldsymbol{\psi}_i) \widetilde{\mathbf{V}}_i(\widehat{\boldsymbol{\nu}}_i^{(k)}) \right] \Bigg\}.\end{aligned}$$

bioRxiv preprint doi: https://doi.org/10.64898/2026.04.27.720955; this version posted April 30, 2026. The copyright holder for this preprint (which was not certified by peer review) is the author/funder, who has granted bioRxiv a license to display the preprint in perpetuity. It is made available under aCC-BY 4.0 International license.

S3.2.2. *CM-Step.* In a traditional M-step, we would compute $\widehat{\boldsymbol{\nu}}^{(k+1)} = \operatorname{argmax}_{\boldsymbol{\nu}} Q(\boldsymbol{\nu} \mid \widehat{\boldsymbol{\nu}}^{(k)})$ (or $\widetilde{Q}(\boldsymbol{\nu} \mid \widehat{\boldsymbol{\nu}}^{(k)})$ for our E-step approximation). However, as closed form solutions for a joint maximization in all parameters are not available and numerical approximations are computationally intractable, we instead perform a conditional maximization step, where we alternate between maximizing $\widetilde{Q}(\boldsymbol{\nu} \mid \widehat{\boldsymbol{\nu}}^{(k)})$ with respect to the sample-specific covariance parameters $\boldsymbol{\psi}_i$ and the shared regression parameter $\boldsymbol{\beta}$.

Note that $\widetilde{Q}(\boldsymbol{\nu} \mid \widehat{\boldsymbol{\nu}}^{(k)})$ depends on $\widehat{\boldsymbol{\nu}}^{(k)}$ only via the conditional means $\widetilde{\boldsymbol{\eta}}_i(\widehat{\boldsymbol{\nu}}_i^{(k)})$ and covariances $\widetilde{\mathbf{V}}_i(\widehat{\boldsymbol{\nu}}_i^{(k)})$. These are constants in the CM-step and are only updated after completion of the CM-step, when $\widehat{\boldsymbol{\nu}}^{(k+1)}$ is determined and the next E-Step is performed. Thus, for simplicity in describing the CM-step, we will use the short-hand notation $\widehat{\boldsymbol{\eta}}_i$ and $\widehat{\mathbf{V}}_i$ for $\widetilde{\boldsymbol{\eta}}_i(\widehat{\boldsymbol{\nu}}_i^{(k)})$ and $\widetilde{\mathbf{V}}_i(\widehat{\boldsymbol{\nu}}_i^{(k)})$, respectively, and write

$$\begin{aligned}
\widetilde{Q}(\boldsymbol{\nu} \mid \widehat{\boldsymbol{\nu}}^{(k)}) &= \widetilde{Q}(\boldsymbol{\nu} \mid \widehat{\boldsymbol{\eta}}, \widehat{\mathbf{V}}) \\
&= \widetilde{Q}(\boldsymbol{\beta}, \boldsymbol{\psi}_1, \ldots, \boldsymbol{\psi}_n \mid \widehat{\boldsymbol{\eta}}, \widehat{\mathbf{V}}) \\
&= \sum_{i=1}^{n} \widetilde{Q}_i(\boldsymbol{\beta}, \boldsymbol{\psi}_i \mid \widehat{\boldsymbol{\eta}}_i, \widehat{\mathbf{V}}_i),
\end{aligned}$$

i.e., the sum of the approximate expected conditional log-likelihoods $\widetilde{Q}_i(\boldsymbol{\beta}, \boldsymbol{\psi}_i \mid \widehat{\boldsymbol{\eta}}_i, \widehat{\mathbf{V}}_i)$ of each sample i. Thus, maximizing $\widetilde{Q}(\boldsymbol{\nu} \mid \widehat{\boldsymbol{\eta}}, \widehat{\mathbf{V}})$ with respect to only the $\boldsymbol{\psi}_1, \ldots, \boldsymbol{\psi}_n$ is equivalent to individually maximizing each $\widetilde{Q}_i(\boldsymbol{\beta}, \boldsymbol{\psi}_i \mid \widehat{\boldsymbol{\eta}}_i, \widehat{\mathbf{V}}_i)$ with respect to $\boldsymbol{\psi}_i$.

The $(k'+1)$st iteration of the CM-step, nested within an iteration k of the ECM algorithm, is then as follows.

1. For each sample i, maximize $\widetilde{Q}_i(\widehat{\boldsymbol{\beta}}^{(k')}, \boldsymbol{\psi}_i | \widehat{\boldsymbol{\eta}}_i, \widehat{\mathbf{V}}_i)$ with respect to $\boldsymbol{\psi}_i$, resulting in an update

$$\widehat{\boldsymbol{\psi}}_i^{(k'+1)} = \widehat{\boldsymbol{\psi}}_i(\widehat{\boldsymbol{\beta}}^{(k')}),$$

   where the notation $\widehat{\boldsymbol{\psi}}_i(\widehat{\boldsymbol{\beta}}^{(k')})$ indicates the dependence on the prior CM iteration's estimate of $\boldsymbol{\beta}$. The maximization in $\boldsymbol{\psi}$ is performed one element at a time, holding the others constant, e.g., in τ^2 holding γ constant, and then in γ holding τ^2 constant, for the lattice models. We derive $\widehat{\boldsymbol{\psi}}_i(\boldsymbol{\beta})$ for each covariance model in Section S3.3 below. Note that there are further approximations needed for the maximization of $\widetilde{Q}_i$ for the spNNGP covariance model, discussed in Section S3.3.6.
2. Maximize $\widetilde{Q}(\boldsymbol{\beta}, \boldsymbol{\psi}_1^{(k'+1)}, \ldots, \boldsymbol{\psi}_n^{(k'+1)} \mid \widehat{\boldsymbol{\eta}}, \widehat{\mathbf{V}})$ with respect to $\boldsymbol{\beta}$, resulting in an update

$$\widehat{\boldsymbol{\beta}}^{(k'+1)} = \widehat{\boldsymbol{\beta}}(\widehat{\boldsymbol{\psi}}_1^{(k'+1)}, \ldots, \widehat{\boldsymbol{\psi}}_n^{(k'+1))}).$$

   $\widehat{\boldsymbol{\beta}}(\boldsymbol{\psi}_1, \ldots, \boldsymbol{\psi}_n)$ is derived in Section S3.3, Equation (25), and is the same for all covariance models.

These two steps are iterated until the expected log-likelihood $\widetilde{Q}$ stabilizes, at which point $\widehat{\boldsymbol{\nu}}^{(k+1)}$ (corresponding to the $(k+1)$st step of the overall ECM algorithm) is set using the final values,

$$\widehat{\boldsymbol{\nu}}^{(k+1)} = (\widehat{\boldsymbol{\beta}}^{(*)}, \widehat{\boldsymbol{\psi}}_1^{(*)}, \ldots, \widehat{\boldsymbol{\psi}}_n^{(*)}),$$

and the algorithm returns to the E-step.

bioRxiv preprint doi: https://doi.org/10.64898/2026.04.27.720955; this version posted April 30, 2026. The copyright holder for this preprint (which was not certified by peer review) is the author/funder, who has granted bioRxiv a license to display the preprint in perpetuity. It is made available under aCC-BY 4.0 International license.

S3.2.3. *Initialization.* For $k = 0$, we initialize $\boldsymbol{\nu}$ with simple estimators. For $\boldsymbol{\beta}$, we use a Poisson GLM. For the spatial parameters $\boldsymbol{\psi}$ in a spNNGP model, we use the `BRISC` method applied to the log-transformed counts. In the lattice models, we initialize τ^2 with the variance of the log-transformed counts and γ with the Moran's I of the counts in each sample. There is no initialization needed for the CM-step; the updates are either closed form (all parameters in the lattice-based covariance models) or rely on `BRISC` for the covariance parameters of the spNNGP covariance model.

S3.2.4. *Variance Estimation.* In addition to the detailed derivations for the CM-step updates, the Hessians for each parameter are given in Section S3.3. The Hessian derivations are used to verify that the estimated parameters actually correspond to local maxima. As the Hessian is equal to the negative of the observed information, it yields an estimate of the variance of the estimators. Due to the lack of identifiability in the spNNGP model (discussed in Section S4), as well as the slightly different procedure for maximizing $\widetilde{Q}_i$ with respect to $\boldsymbol{\psi}_i$, we do not provide Hessian matrices for the spatial parameters in this model.

S3.2.5. *Convergence to Global Optima.* As noted, the `TESSERA` algorithm relies on an approximation of the expected log-likelihood in the E-Step. While this approximation is generally good (and improves for larger counts), it is still an approximation and a potential source of error (Clayton and Kaldor, 1987). Moreover, the ECM algorithm, like the EM algorithm, can only guarantee convergence to a local optimum of the log-likelihood, as opposed to a global optimum (Meng and Rubin, 1993).

However, we may offer the following weaker guarantee. As detailed above, each of the CM-steps optimizes in a single parameter while holding the others fixed (where we treat $\boldsymbol{\beta}$ as a single parameter in this discussion). As long as the precision matrices $\mathbf{P}_i$ are invertible, the single-parameter Hessians of the approximated expected log-likelihood $\widetilde{Q}$ are all negative definite, so that each CM-step update has a unique solution. Hence, the sequence of CM-steps does not decrease $\widetilde{Q}$.

S3.2.6. *Prediction.* Given estimated parameter values for $\boldsymbol{\nu}$, we can perform prediction of the counts. Using the fact that the estimator that minimizes risk for the squared error loss is the conditional (posterior) mean, we may estimate $\theta_{i,j}$ with

$$\mathbb{E}\left[\theta_{i,j} \mid \mathbf{Z}_i, \mathbf{X}_i\right] = \mathbb{E}\left[\exp\left(\eta_{i,j}\right) \mid \mathbf{Z}_i, \mathbf{X}_i\right].$$

Using the approximate normality of $\eta_{i,j} \mid \mathbf{Z}_i, \mathbf{X}_i$, we have that $e^{\eta_{i,j}} \mid \mathbf{Z}_i, \mathbf{X}_i$ follows a log-normal distribution, giving us

$$\widehat{\theta}_{i,j} = \exp\left(\widehat{\eta}_{i,j} + \frac{1}{2}[\widehat{\mathbf{V}}_i]_{j,j}\right), \tag{21}$$

where $[\widehat{\mathbf{V}}_i]_{j,j}$ denotes the diagonal elements of the matrix $\widehat{\mathbf{V}}_i$ and $\widehat{\boldsymbol{\eta}}_i$ and $\widehat{\mathbf{V}}_i$ are obtained by plugging in $\widehat{\boldsymbol{\nu}}$ in Equations (19) and (20), respectively. It follows that the fitted values of the counts $Z_{i,j}$ are given by

$$\widehat{Z}_{i,j} = \max\left\{0, \widehat{\theta}_{i,j} C_{i,j} - \frac{1}{2}\right\}. \tag{22}$$

S3.3. *Derivation of `TESSERA` CM-step Updates.* In the CM-step, we seek to maximize

$$\begin{aligned} \widetilde{Q}(\boldsymbol{\nu} \mid \widehat{\boldsymbol{\nu}}) = \sum_{i=1}^{n} \Bigg\{ & \frac{1}{2} \log\det \mathbf{P}_i(\boldsymbol{\psi}_i) \\ & - \frac{1}{2}\left(\widehat{\boldsymbol{\eta}}_i - \mathbf{X}_i\boldsymbol{\beta}\right)^\top \mathbf{P}_i(\boldsymbol{\psi}_i)\left(\widehat{\boldsymbol{\eta}}_i - \mathbf{X}_i\boldsymbol{\beta}\right) - \frac{1}{2}\operatorname{Tr}\left[\mathbf{P}_i(\boldsymbol{\psi}_i)\widehat{\mathbf{V}}_i\right] \Bigg\} \end{aligned} \tag{23}$$

bioRxiv preprint doi: https://doi.org/10.64898/2026.04.27.720955; this version posted April 30, 2026. The copyright holder for this preprint (which was not certified by peer review) is the author/funder, who has granted bioRxiv a license to display the preprint in perpetuity. It is made available under aCC-BY 4.0 International license.

with respect to $\boldsymbol{\nu} = (\boldsymbol{\beta}, \boldsymbol{\psi}_1, \ldots, \boldsymbol{\psi}_n)$, by alternating maximization steps over the sample-specific covariance parameters $\boldsymbol{\psi}_i$ and the common regression parameter $\boldsymbol{\beta}$, where $\widehat{\boldsymbol{\nu}}$ denote the previous iteration's estimated parameters and $\widehat{\boldsymbol{\eta}}_i$ and $\widehat{\mathbf{V}}_i$ the corresponding estimated conditional means and covariances for $\boldsymbol{\eta}_i$ computed according to Equations (19) and (20), respectively.

We first present the CM-step update for $\boldsymbol{\beta}$, which applies to all covariance models. For lattice models, $\mathbf{P}_i(\boldsymbol{\psi}_i) = (1/\tau_i^2)\,\mathbf{P}_i^*(\gamma_i)$ and we present below the CM-step updates for τ^2 and γ. The update for τ^2 is the same for all lattice models, whereas the update for γ is different for each of the CAR, SAR, and Leroux parameterizations of $\mathbf{P}^*$.

S3.3.1. *All Covariance Models: $\boldsymbol{\beta}$.* Assume that we have a precision matrix $\mathbf{P}_i(\boldsymbol{\psi}_i)$ for sample i. Assume further that all spatial parameters $\boldsymbol{\psi}_i$ are held fixed and, for notational simplicity, write $\mathbf{P}_i$ instead of $\mathbf{P}_i(\boldsymbol{\psi}_i)$. Differentiating $\widetilde{Q}$ from Equation (23) with respect to $\boldsymbol{\beta}$ yields

$$\nabla_{\boldsymbol{\beta}} \widetilde{Q}(\boldsymbol{\nu} \,|\, \widehat{\boldsymbol{\nu}}) = \sum_{i=1}^{n} \left[\mathbf{X}_i^\top \mathbf{P}_i \widehat{\boldsymbol{\eta}}_i - \mathbf{X}_i^\top \mathbf{P}_i \mathbf{X}_i \boldsymbol{\beta} \right] \in \mathbb{R}^p .$$

If we define

$$\mathbf{B} = \sum_{i=1}^{n} \mathbf{X}_i^\top \mathbf{P}_i \mathbf{X}_i \in \mathbb{R}^{p \times p}, \tag{24}$$

setting the gradient equal to zero yields that, for fixed $\boldsymbol{\psi}_i$, $\widetilde{Q}$ is maximized by

$$\widehat{\boldsymbol{\beta}}(\boldsymbol{\psi}_1, \ldots, \boldsymbol{\psi}_n) = \mathbf{B}^{-1} \left[\sum_{i=1}^{n} \mathbf{X}_i^\top \mathbf{P}_i \widehat{\boldsymbol{\eta}}_i \right] = \left[\sum_{i=1}^{n} \mathbf{X}_i^\top \mathbf{P}_i \mathbf{X}_i \right]^{-1} \left[\sum_{i=1}^{n} \mathbf{X}_i^\top \mathbf{P}_i \widehat{\boldsymbol{\eta}}_i \right]. \tag{25}$$

We note that the form of this estimator is that of a generalized least squares estimator. Defining a block-diagonal matrix of the $\mathbf{P}_i$ and stacking the rows of $\mathbf{X}_i$ accordingly yields the conclusion. Differentiating $\widetilde{Q}$ again yields the negative of the observed information equal to the matrix $-\mathbf{B}$. Note that $\mathbf{B}$ depends on the spatial covariance parameters $\boldsymbol{\psi}_i$ via the $\mathbf{P}_i$. Assuming that the $\mathbf{P}_i$ have full rank (i.e., are positive-definite and proper precision matrices) and that the $\mathbf{X}_i$ have full column rank, $\mathbf{X}_i^\top \mathbf{P}_i \mathbf{X}_i$ will have full rank and be positive-definite. Since $\mathbf{B}$ is the sum of positive-definite matrices, it is positive-definite, so that $-\mathbf{B}$ will be negative-definite and $\widehat{\boldsymbol{\beta}}$ is a maximizer of $\widetilde{Q}$.

S3.3.2. *All Lattice Models: τ^2.* We drop the sample index i for convenience, as differentiating in τ_i^2 will only involve the terms with index i. Assume that $\boldsymbol{\beta}$ and γ are fixed; then $\mathbf{P}^*(\gamma)$ is also fixed and denoted simply by $\mathbf{P}^*$. The gradient of $\widetilde{Q}$ with respect to τ^2 is

$$\nabla_{\tau^2} \widetilde{Q}(\boldsymbol{\nu} \,|\, \widehat{\boldsymbol{\nu}}) = -\frac{J}{2\tau^2} + \frac{1}{2(\tau^2)^2} \left[(\widehat{\boldsymbol{\eta}} - \mathbf{X}\boldsymbol{\beta})^\top \mathbf{P}^* (\widehat{\boldsymbol{\eta}} - \mathbf{X}\boldsymbol{\beta}) + \mathrm{Tr}[\mathbf{P}^* \widehat{\mathbf{V}}] \right].$$

Setting the gradient equal to zero, it follows that the estimator for τ^2 is

$$\widehat{\tau^2}(\boldsymbol{\beta}, \gamma) = \frac{1}{J} \left[(\widehat{\boldsymbol{\eta}} - \mathbf{X}\boldsymbol{\beta})^\top \mathbf{P}^* (\widehat{\boldsymbol{\eta}} - \mathbf{X}\boldsymbol{\beta}) + \mathrm{Tr}[\mathbf{P}^* \widehat{\mathbf{V}}] \right]. \tag{26}$$

Differentiating again yields the negative of the observed information

$$\nabla^2_{\tau^2} \widetilde{Q}(\boldsymbol{\nu} \,|\, \widehat{\boldsymbol{\nu}}) = \frac{J}{2} \frac{1}{(\tau^2)^2} - \frac{1}{(\tau^2)^3} \left[(\widehat{\boldsymbol{\eta}} - \mathbf{X}\boldsymbol{\beta})^\top \mathbf{P}^* (\widehat{\boldsymbol{\eta}} - \mathbf{X}\boldsymbol{\beta}) + \mathrm{Tr}[\mathbf{P}^* \widehat{\mathbf{V}}] \right].$$

bioRxiv preprint doi: https://doi.org/10.64898/2026.04.27.720955; this version posted April 30, 2026. The copyright holder for this preprint (which was not certified by peer review) is the author/funder, who has granted bioRxiv a license to display the preprint in perpetuity. It is made available under aCC-BY 4.0 International license.

We note that if

$$0 < \frac{J}{2}\frac{1}{(\tau^2)^2} < \frac{1}{(\tau^2)^3}\left[(\widehat{\boldsymbol{\eta}} - \mathbf{X}\boldsymbol{\beta})^\top \mathbf{P}^*(\widehat{\boldsymbol{\eta}} - \mathbf{X}\boldsymbol{\beta}) + \mathrm{Tr}[\mathbf{P}^*\widehat{\mathbf{V}}]\right],$$

then the Hessian is strictly negative. Simplifying the above, we see that we need

$$0 < \tau^2 < \frac{2}{J}\left[(\widehat{\boldsymbol{\eta}} - \mathbf{X}\boldsymbol{\beta})^\top \mathbf{P}^*(\widehat{\boldsymbol{\eta}} - \mathbf{X}\boldsymbol{\beta}) + \mathrm{Tr}[\mathbf{P}^*\widehat{\mathbf{V}}]\right] = 2\widehat{\tau^2},$$

so that the Hessian is always negative at $\widehat{\tau^2}$.

S3.3.3. *γ in the CAR Model.* In what follows, we omit the sample index i for convenience. Assume that $\boldsymbol{\beta}$ and τ^2 are fixed. In the CAR model, we have that $\mathbf{P}^* = (\mathbf{D} - \gamma\,\mathbf{W})$, so that the relevant portion of $\widetilde{Q}(\boldsymbol{\nu} \mid \widehat{\boldsymbol{\nu}})$ is

$$\frac{1}{2}\log\det[\mathbf{D} - \gamma\,\mathbf{W}] + \gamma\left[\frac{1}{2\tau^2}(\widehat{\boldsymbol{\eta}} - \mathbf{X}\boldsymbol{\beta})^\top \mathbf{W}(\widehat{\boldsymbol{\eta}} - \mathbf{X}\boldsymbol{\beta})\right] + \gamma\frac{1}{2\tau^2}\mathrm{Tr}[\mathbf{W}\widehat{\mathbf{V}}].$$

Differentiating the first term with respect to γ yields

$$-\frac{1}{2}\mathrm{Tr}\left[(\mathbf{D} - \gamma\,\mathbf{W})^{-1}\mathbf{W}\right]$$

and then differentiating the second and third terms yields

$$\frac{1}{2\tau^2}\left((\widehat{\boldsymbol{\eta}} - \mathbf{X}\boldsymbol{\beta})^\top \mathbf{W}(\widehat{\boldsymbol{\eta}} - \mathbf{X}\boldsymbol{\beta}) + \mathrm{Tr}[\mathbf{W}\widehat{\mathbf{V}}]\right),$$

for an overall gradient of

$$\nabla_\gamma \widetilde{Q}(\boldsymbol{\nu} \mid \widehat{\boldsymbol{\nu}}) = -\frac{1}{2}\mathrm{Tr}[(\mathbf{D} - \gamma\,\mathbf{W})^{-1}\mathbf{W}] + \frac{1}{2\tau^2}\left[(\widehat{\boldsymbol{\eta}} - \mathbf{X}\boldsymbol{\beta})^\top \mathbf{W}(\widehat{\boldsymbol{\eta}} - \mathbf{X}\boldsymbol{\beta}) + \mathrm{Tr}[\mathbf{W}\widehat{\mathbf{V}}]\right].$$

Using the invertibility of $\mathbf{D}$ and $\mathbf{W}$, we may write

$$\mathbf{D} - \gamma\,\mathbf{W} = \mathbf{D}\left(\mathbf{I} - \gamma\,\mathbf{D}^{-1}\mathbf{W}\right)$$

or

$$(\mathbf{D} - \gamma\,\mathbf{W})^{-1} = \left(\mathbf{I} - \gamma\,\mathbf{D}^{-1}\mathbf{W}\right)^{-1}\mathbf{D}^{-1}.$$

If we let $\mathbf{A} = \mathbf{D}^{-1}\mathbf{W}$, we may write the first term of the gradient as

$$-\frac{1}{2}\mathrm{Tr}[(\mathbf{I} - \gamma\,\mathbf{A})^{-1}\mathbf{A}].$$

Assuming that $\mathbf{A}$ has an eigendecomposition $\mathbf{A} = \mathbf{U}\,\boldsymbol{\Lambda}\,\mathbf{U}^{-1}$, where $\boldsymbol{\Lambda}$ has diagonal entries λ_j, $j = 1, \ldots, J$, we may write

$$\begin{aligned}
(\mathbf{I} - \gamma\,\mathbf{A})^{-1}\mathbf{A} &= \left[\mathbf{U}\,\mathbf{I}\,\mathbf{U}^{-1} - \mathbf{U}(\gamma\,\boldsymbol{\Lambda})\,\mathbf{U}^{-1}\right]^{-1}\mathbf{U}\,\boldsymbol{\Lambda}\,\mathbf{U}^{-1} \\
&= \mathbf{U}\,(\mathbf{I} - \gamma\,\boldsymbol{\Lambda})^{-1}\,\mathbf{U}^{-1}\,\mathbf{U}\,\boldsymbol{\Lambda}\,\mathbf{U}^{-1} \\
&= \mathbf{U}\,(\mathbf{I} - \gamma\,\boldsymbol{\Lambda})^{-1}\,\boldsymbol{\Lambda}\,\mathbf{U}^{-1}.
\end{aligned}$$

Taking the trace yields

$$\begin{aligned}
\mathrm{Tr}[(\mathbf{I} - \gamma\,\mathbf{A})^{-1}\mathbf{A}] &= \mathrm{Tr}[\mathbf{U}\,(\mathbf{I} - \gamma\,\boldsymbol{\Lambda})^{-1}\,\boldsymbol{\Lambda}\,\mathbf{U}^{-1}] \\
&= \mathrm{Tr}[\mathbf{U}^{-1}\,\mathbf{U}\,(\mathbf{I} - \gamma\,\boldsymbol{\Lambda})^{-1}\,\boldsymbol{\Lambda}] \\
&= \mathrm{Tr}[(\mathbf{I} - \gamma\,\boldsymbol{\Lambda})^{-1}\,\boldsymbol{\Lambda}] \\
&= \sum_{j=1}^{J}\frac{\lambda_j}{1 - \gamma\lambda_j}.
\end{aligned}$$

bioRxiv preprint doi: https://doi.org/10.64898/2026.04.27.720955; this version posted April 30, 2026. The copyright holder for this preprint (which was not certified by peer review) is the author/funder, who has granted bioRxiv a license to display the preprint in perpetuity. It is made available under aCC-BY 4.0 International license.

It follows that the gradient in γ is

$$\nabla_\gamma \widetilde{Q}(\boldsymbol{\nu} \mid \widehat{\boldsymbol{\nu}}) = -\frac{1}{2}\sum_{j=1}^{J} \frac{\lambda_j}{1-\gamma\lambda_j} + \frac{1}{2\tau^2}\left[(\widehat{\boldsymbol{\eta}} - \mathbf{X}\boldsymbol{\beta})^\top \mathbf{W}(\widehat{\boldsymbol{\eta}} - \mathbf{X}\boldsymbol{\beta}) + \mathrm{Tr}[\mathbf{W}\,\widehat{\mathbf{V}}]\right]. \tag{27}$$

We may set the gradient equal to zero and numerically look for a root in $[-1, 1]$ to find the $\widehat{\gamma}(\boldsymbol{\beta}, \tau^2)$ which maximizes $\widetilde{Q}$ for fixed $\boldsymbol{\beta}$ and τ^2. Pre-computing $\mathbf{A}$ and its eigenvalues for each sample leads to an extremely rapid evaluation of the gradient, making the optimization more efficient.

Differentiating again and negating yields the observed information:

$$-\nabla^2_\gamma \widetilde{Q}(\boldsymbol{\nu} \mid \widehat{\boldsymbol{\nu}}) = \frac{1}{2}\sum_{j=1}^{J}\left(\frac{\lambda_j}{1-\gamma\lambda_j}\right)^2.$$

Note that the Hessian is strictly negative, as at least one of the λ_j is non-zero.

S3.3.4. *γ in the SAR Model.* In what follows, we omit the sample index i for convenience. Assume that $\boldsymbol{\beta}$ and τ^2 are fixed. Here, the relevant part of $\widetilde{Q}(\boldsymbol{\nu} \mid \widehat{\boldsymbol{\nu}})$ is

$$\frac{1}{2}\log\det \mathbf{P}^* - \left[\frac{1}{2\tau^2}(\widehat{\boldsymbol{\eta}} - \mathbf{X}\boldsymbol{\beta})^\top \mathbf{P}^*(\widehat{\boldsymbol{\eta}} - \mathbf{X}\boldsymbol{\beta})\right] - \frac{1}{2\tau^2}\mathrm{Tr}[\mathbf{P}^*\,\widehat{\mathbf{V}}].$$

In the SAR model, we have that

$$\mathbf{P}^* = \left(\mathbf{I} - \gamma\,\mathbf{D}^{-1}\mathbf{W}\right)^\top \mathbf{D}\left(\mathbf{I} - \gamma\,\mathbf{D}^{-1}\mathbf{W}\right).$$

Reusing the definition of $\mathbf{A} = \mathbf{D}^{-1}\mathbf{W}$, we may expand $\mathbf{P}^*$ as

$$\begin{aligned}\mathbf{P}^* &= (\mathbf{I} - \gamma\,\mathbf{A})^\top \mathbf{D}\,(\mathbf{I} - \gamma\,\mathbf{A}) \\ &= (\mathbf{D} - \gamma\,\mathbf{A}^\top\mathbf{D})(\mathbf{I} - \gamma\,\mathbf{A}) \\ &= \mathbf{D} - \gamma(\mathbf{A}^\top\mathbf{D} + \mathbf{D}\,\mathbf{A}) + \gamma^2\,\mathbf{A}^\top\mathbf{D}\,\mathbf{A}.\end{aligned}$$

Note that since $\mathbf{A} = \mathbf{D}^{-1}\mathbf{W}$ and $(\mathbf{D}\,\mathbf{A})^\top = \mathbf{A}^\top\mathbf{D}$, we may write $\mathbf{D}\,\mathbf{A} = \mathbf{D}\,\mathbf{D}^{-1}\mathbf{W} = \mathbf{W}$, so that $\mathbf{A}^\top\mathbf{D} + \mathbf{D}\,\mathbf{A} = 2\,\mathbf{W}$. Similarly,

$$\mathbf{A}^\top\mathbf{D}\,\mathbf{A} = \mathbf{A}^\top\mathbf{W} = (\mathbf{D}^{-1}\mathbf{W})^\top\mathbf{W} = \mathbf{W}^\top\mathbf{D}^{-1}\mathbf{W} = \mathbf{W}\,\mathbf{A}.$$

If we define

$$\zeta_1 = (\widehat{\boldsymbol{\eta}} - \mathbf{X}\boldsymbol{\beta})^\top(\mathbf{D}\,\mathbf{A} + \mathbf{A}^\top\mathbf{D})(\widehat{\boldsymbol{\eta}} - \mathbf{X}\boldsymbol{\beta}) = 2(\widehat{\boldsymbol{\eta}} - \mathbf{X}\boldsymbol{\beta})^\top\mathbf{W}(\widehat{\boldsymbol{\eta}} - \mathbf{X}\boldsymbol{\beta})$$

and

$$\zeta_2 = (\widehat{\boldsymbol{\eta}} - \mathbf{X}\boldsymbol{\beta})^\top(\mathbf{A}^\top\mathbf{D}\,\mathbf{A})(\widehat{\boldsymbol{\eta}} - \mathbf{X}\boldsymbol{\beta}) = (\widehat{\boldsymbol{\eta}} - \mathbf{X}\boldsymbol{\beta})^\top\mathbf{W}\,\mathbf{A}(\widehat{\boldsymbol{\eta}} - \mathbf{X}\boldsymbol{\beta}),$$

we may write the relevant part of the second term of $\widetilde{Q}$ as

$$-\frac{1}{2\tau^2}\left(-\zeta_1\gamma + \zeta_2\gamma^2\right).$$

Differentiating the above with respect to γ yields

$$-\frac{1}{2\tau^2}\left(-\zeta_1 + 2\zeta_2\gamma\right).$$

By a similar series of derivations, we may write the relevant part of the third term of $\widetilde{Q}$ as

$$-\frac{1}{2\tau^2}\left(-\gamma\,\mathrm{Tr}[2\,\mathbf{W}\,\widehat{\mathbf{V}}] + \gamma^2\,\mathrm{Tr}[\mathbf{W}\,\mathbf{A}\,\widehat{\mathbf{V}}]\right).$$

bioRxiv preprint doi: https://doi.org/10.64898/2026.04.27.720955; this version posted April 30, 2026. The copyright holder for this preprint (which was not certified by peer review) is the author/funder, who has granted bioRxiv a license to display the preprint in perpetuity. It is made available under aCC-BY 4.0 International license.

Differentiating in γ yields

$$-\frac{1}{\tau^2}\left(-\operatorname{Tr}[\mathbf{W}\,\widehat{\mathbf{V}}] + \gamma\operatorname{Tr}[\mathbf{W}\,\mathbf{A}\,\widehat{\mathbf{V}}]\right).$$

For the first term of $\widetilde{Q}$, note that

$$\det \mathbf{P}^* = \det[\mathbf{I} - \gamma\,\mathbf{D}^{-1}\,\mathbf{W}]^2 \times \det \mathbf{D},$$

so that

$$\log\det \mathbf{P}^* = 2\log\det[\mathbf{I} - \gamma\,\mathbf{D}^{-1}\,\mathbf{W}] + \log\det \mathbf{D}\,.$$

Differentiating this in γ, using Equation (39), yields

$$-2\operatorname{Tr}[(\mathbf{I} - \gamma\,\mathbf{A})^{-1}\,\mathbf{A}] = -2\sum_{j=1}^{J}\frac{\lambda_j}{1-\gamma\lambda_j}.$$

It follows that the overall gradient is
(28)

$$\nabla_\gamma\widetilde{Q}(\boldsymbol{\nu}\mid\widehat{\boldsymbol{\nu}}) = -\sum_{j=1}^{J}\frac{\lambda_j}{1-\gamma\lambda_j} - \frac{1}{2\tau^2}(-\zeta_1 + 2\zeta_2\gamma) - \frac{1}{\tau^2}\left(-\operatorname{Tr}[\mathbf{W}\,\widehat{\mathbf{V}}] + \gamma\operatorname{Tr}[\mathbf{W}\,\mathbf{A}\,\widehat{\mathbf{V}}]\right).$$

We may set the gradient equal to zero and numerically look for a root in $[-1,1]$ to find the $\widehat{\gamma}(\boldsymbol{\beta},\tau^2)$ which maximizes $\widetilde{Q}$ for fixed $\boldsymbol{\beta}$ and τ^2. Note that the overall form of the gradient is very similar to that in the CAR model.

If we differentiate again in γ and negate the result, we find an observed information of

$$-\nabla^2_\gamma\widetilde{Q}(\boldsymbol{\nu}\mid\widehat{\boldsymbol{\nu}}) = \sum_{j=1}^{J}\left(\frac{\lambda_j}{1-\gamma\lambda_j}\right)^2 + \frac{1}{\tau^2}\left(\zeta_2 + \operatorname{Tr}[\mathbf{W}\,\mathbf{A}\,\widehat{\mathbf{V}}]\right).$$

We note that the first term of the Hessian is strictly negative, $\zeta_2 \geq 0$, and $\widehat{\mathbf{V}}$ and $\mathbf{W}$ are positive-semidefinite; however, we cannot conclude that the Hessian is strictly negative unless the product $\mathbf{W}\,\mathbf{A}\,\widehat{\mathbf{V}}$ has a postive trace.

S3.3.5. *γ in the Leroux Model.* In what follows, we omit the sample index i for convenience. Assume that $\boldsymbol{\beta}$ and τ^2 are fixed. In the Leroux model, we have that

$$\mathbf{P}^* = \gamma(\mathbf{D} - \mathbf{W}) + (1-\gamma)\,\mathbf{I} = \gamma(\mathbf{D} - \mathbf{W} - \mathbf{I}) + \mathbf{I},$$

so that the relevant part of $\widetilde{Q}(\boldsymbol{\nu}\mid\widehat{\boldsymbol{\nu}})$ is

$$\frac{1}{2}\log\det\mathbf{P}^* - \left[\frac{1}{2\tau^2}(\widehat{\boldsymbol{\eta}} - \mathbf{X}\boldsymbol{\beta})^\top\,\mathbf{P}^*(\widehat{\boldsymbol{\eta}} - \mathbf{X}\boldsymbol{\beta})\right] - \frac{1}{2\tau^2}\operatorname{Tr}[\mathbf{P}^*\,\widehat{\mathbf{V}}].$$

Considering the first (log-determinant) term, we may write

$$\frac{1}{2}\log\det\mathbf{P}^* = \frac{1}{2}\log\det\left[\mathbf{I} + \gamma(\mathbf{D} - \mathbf{W} - \mathbf{I})\right].$$

Differentiating in γ yields a gradient (of this term) of

$$\frac{1}{2}\operatorname{Tr}\left[(\mathbf{I} + \gamma\,(\mathbf{D} - \mathbf{W} - \mathbf{I}))^{-1}\,(\mathbf{D} - \mathbf{W} - \mathbf{I})\right] = \frac{1}{2}\operatorname{Tr}\left[(\mathbf{P}^*)^{-1}(\mathbf{D} - \mathbf{W} - \mathbf{I})\right].$$

bioRxiv preprint doi: https://doi.org/10.64898/2026.04.27.720955; this version posted April 30, 2026. The copyright holder for this preprint (which was not certified by peer review) is the author/funder, who has granted bioRxiv a license to display the preprint in perpetuity. It is made available under aCC-BY 4.0 International license.

If we write the eigendecomposition of $\mathbf{D}-\mathbf{W}=\mathbf{U}\,\boldsymbol{\Lambda}\,\mathbf{U}^{-1}$, where $\boldsymbol{\Lambda}$ has diagonal entries λ_j, for $j=1,\ldots,J$, we have that

$$\begin{aligned}
\mathbf{D}-\mathbf{W} &= \mathbf{U}\,\boldsymbol{\Lambda}\,\mathbf{U}^{-1}\\
\mathbf{D}-\mathbf{W}-\mathbf{I} &= \mathbf{U}\,(\boldsymbol{\Lambda}-\mathbf{I})\,\mathbf{U}^{-1}\\
\gamma(\mathbf{D}-\mathbf{W}-\mathbf{I}) &= \mathbf{U}\,[\gamma\,(\boldsymbol{\Lambda}-\mathbf{I})]\,\mathbf{U}^{-1}\\
\mathbf{P}^* = \gamma(\mathbf{D}-\mathbf{W}-\mathbf{I})+\mathbf{I} &= \mathbf{U}\,[\mathbf{I}+\gamma\,(\boldsymbol{\Lambda}-\mathbf{I})]\,\mathbf{U}^{-1}\\
(\mathbf{P}^*)^{-1} = [\gamma\,(\mathbf{D}-\mathbf{W}-\mathbf{I})+\mathbf{I}]^{-1} &= \mathbf{U}\,[\mathbf{I}+\gamma\,(\boldsymbol{\Lambda}-\mathbf{I})]^{-1}\,\mathbf{U}^{-1}.
\end{aligned}$$

Then, we have that

$$\begin{aligned}
&\frac{1}{2}\,\mathrm{Tr}\left[(\mathbf{I}+\gamma(\mathbf{D}-\mathbf{W}-\mathbf{I}))^{-1}\,(\mathbf{D}-\mathbf{W}-\mathbf{I})\right]\\
&=\frac{1}{2}\,\mathrm{Tr}\left[\mathbf{U}\,(\mathbf{I}+\gamma\,(\boldsymbol{\Lambda}-\mathbf{I}))^{-1}\,\mathbf{U}^{-1}\cdot\mathbf{U}\,(\boldsymbol{\Lambda}-\mathbf{I})\,\mathbf{U}^{-1}\right]\\
&=\frac{1}{2}\,\mathrm{Tr}\left[\mathbf{U}^{-1}\,\mathbf{U}(\mathbf{I}+\gamma\,(\boldsymbol{\Lambda}-\mathbf{I}))^{-1}\,(\boldsymbol{\Lambda}-\mathbf{I})\right]\\
&=\frac{1}{2}\,\mathrm{Tr}\left[(\mathbf{I}+\gamma\,(\boldsymbol{\Lambda}-\mathbf{I}))^{-1}\,(\boldsymbol{\Lambda}-\mathbf{I})\right]\\
&=\frac{1}{2}\sum_{j=1}^{J}\frac{(\lambda_j-1)}{(\lambda_j-1)\gamma+1}.
\end{aligned}$$

The second term of $\widetilde{Q}$ can be written as

$$-\frac{1}{2\tau^2}(\widehat{\boldsymbol{\eta}}-\mathbf{X}\boldsymbol{\beta})^\top\,[\mathbf{I}+\gamma(\mathbf{D}-\mathbf{W}-\mathbf{I})]\,(\widehat{\boldsymbol{\eta}}-\mathbf{X}\boldsymbol{\beta}).$$

Isolating the part that contains γ yields

$$-\frac{\gamma}{2\tau^2}(\widehat{\boldsymbol{\eta}}-\mathbf{X}\boldsymbol{\beta})^\top\,[\mathbf{D}-\mathbf{W}-\mathbf{I}]\,(\widehat{\boldsymbol{\eta}}-\mathbf{X}\boldsymbol{\beta}),$$

so that the gradient of this term is simply

$$-\frac{1}{2\tau^2}(\widehat{\boldsymbol{\eta}}-\mathbf{X}\boldsymbol{\beta})^\top\,[\mathbf{D}-\mathbf{W}-\mathbf{I}]\,(\widehat{\boldsymbol{\eta}}-\mathbf{X}\boldsymbol{\beta}).$$

The relevant part of the final trace term is

$$-\frac{1}{2\tau^2}\,\mathrm{Tr}[\gamma(\mathbf{D}-\mathbf{W}-\mathbf{I})\widehat{\mathbf{V}}],$$

where differentiating yields

$$-\frac{1}{2\tau^2}\,\mathrm{Tr}[(\mathbf{D}-\mathbf{W}-\mathbf{I})\widehat{\mathbf{V}}].$$

The overall gradient is then

$$\nabla_\gamma\widetilde{Q}(\boldsymbol{\nu}\mid\widehat{\boldsymbol{\nu}}) = \frac{1}{2}\sum_{j=1}^{J}\frac{(\lambda_j-1)}{(\lambda_j-1)\gamma+1} \tag{29}$$

$$-\frac{1}{2\tau^2}(\widehat{\boldsymbol{\eta}}-\mathbf{X}\boldsymbol{\beta})^\top\,[\mathbf{D}-\mathbf{W}-\mathbf{I}]\,(\widehat{\boldsymbol{\eta}}-\mathbf{X}\boldsymbol{\beta})-\frac{1}{2\tau^2}\,\mathrm{Tr}[(\mathbf{D}-\mathbf{W}-\mathbf{I})\widehat{\mathbf{V}}]. \tag{30}$$

bioRxiv preprint doi: https://doi.org/10.64898/2026.04.27.720955; this version posted April 30, 2026. The copyright holder for this preprint (which was not certified by peer review) is the author/funder, who has granted bioRxiv a license to display the preprint in perpetuity. It is made available under aCC-BY 4.0 International license.

Again, we may set the gradient equal to zero and numerically look for a root in $[0,1]$ to find the $\widehat{\gamma}(\boldsymbol{\beta},\tau^2)$ which maximizes $\widetilde{Q}$ for fixed $\boldsymbol{\beta}$ and τ^2. Much like in the CAR and SAR cases, precomputing the eigenvalues of $\mathbf{D}-\mathbf{W}$ dramatically improves the ability to quickly fit these models.

Differentiating again yields a Hessian (negative of the observed information) of

$$\nabla_\gamma^2 \widetilde{Q}(\boldsymbol{\nu} \mid \widehat{\boldsymbol{\nu}}) = -\sum_{j=1}^{J} \frac{(\lambda_j - 1)^2}{((\lambda_j - 1)\gamma + 1)^2},$$

which is clearly negative.

S3.3.6. *Spatial Correlation Parameters $\boldsymbol{\psi}$ in the spNNGP Model.* Estimation of the parameters $\boldsymbol{\psi}_i$ depends on the kernel function chosen, and performing a proper maximum likelihood optimization procedure has cubic computational complexity as a function of the number of cells in each sample (Saha and Datta, 2018). Instead, we use the `BRISC` algorithm applied to $\widehat{\boldsymbol{\phi}}_i = \widehat{\boldsymbol{\eta}}_i - \mathbf{X}_i\widehat{\boldsymbol{\beta}}$. This procedure has quadratic time complexity and is much more scalable, in addition to being robust and accurate. Given the parameter estimates, we follow Datta et al. (2016) in forming the precision matrix $\mathbf{P}_i$ and the estimation of $\boldsymbol{\beta}$ proceeds as in the lattice models.

S4. Identifiability of Parameters. In this section, we show that under relatively mild conditions, the fixed effects $\boldsymbol{\beta}$ are identifiable in all models we consider. Moreover, we show that the spatial parameters γ and τ^2 are identifiable in the lattice models. Due to the independence of the $\boldsymbol{\eta}$ across samples, it is sufficient to consider a single sample; we hence drop the sample index i where possible (though we include a verification that this single-sample focus is valid in Corollary 2). Moreover, as the counts $\mathbf{Z}$ only depend on the parameters $\boldsymbol{\nu}$ via $\boldsymbol{\eta}$, we need not consider the joint distribution of $\mathbf{Z}$ and $\boldsymbol{\eta}$. That is, it is sufficient to consider the likelihood of $\boldsymbol{\eta} \in \mathbb{R}^J$ conditional on the covariates $\mathbf{X}$. To prove identifiability, we show that if the difference in the log-likelihoods of $\boldsymbol{\eta}$ with two different parameter sets is zero, both sets of parameters must be the same. We first prove that the fixed effects $\boldsymbol{\beta}$ and the precision matrices $\mathbf{P} = \boldsymbol{\Sigma}^{-1}$ are identifiable in Theorem 1. We then prove in Theorem 3 that the spatial parameters γ and τ^2 are identifiable for each of the CAR, SAR, and Leroux models. We discuss the (lack of) identifiablilty of the spatial parameters in a spNNGP model at the end of this section.

THEOREM 1. *Consider the single-sample version of the* `TESSERA` *model of Equation (3), where $\boldsymbol{\eta} \mid \mathbf{X} \sim \mathcal{N}(\mathbf{X}\boldsymbol{\beta}, \boldsymbol{\Sigma})$. If we assume that*

1. *the covariate matrix $\mathbf{X} \in \mathbb{R}^{J\times p}$ has full column rank (so that $p \le J$),*
2. *the spatial parameters are such that the precision matrix $\mathbf{P} = \boldsymbol{\Sigma}^{-1} \in \mathbb{R}^{J\times J}$ of $\boldsymbol{\eta}$ is invertible,*
3. *the number of measurements J is at least 2,*

then we have that the fixed effects $\boldsymbol{\beta} \in \mathbb{R}^p$ and the precision matrix $\mathbf{P} \in \mathbb{R}^{J\times J}$ are identifiable.

Before proceeding, we return to the multi-sample setting.

COROLLARY 2. *Consider the multi-sample* `TESSERA` *model of Equation (3), where $\boldsymbol{\eta}_i \mid \mathbf{X}_i \sim \mathcal{N}(\mathbf{X}_i\boldsymbol{\beta}, \boldsymbol{\Sigma}_i)$ and the $\boldsymbol{\eta}_i$ are independent across samples. If each precision matrix $\mathbf{P}_i$ is invertible, there are at least two measurements across all samples ($\sum_i J_i \ge 2$), and the stacked covariate matrix $\widetilde{\mathbf{X}} \in \mathbb{R}^{\sum_i J_i \times p}$ of the $\mathbf{X}_i$ has full column rank, then $\boldsymbol{\beta}$ and the $\mathbf{P}_i$ are all simultaneously identifiable.*

bioRxiv preprint doi: https://doi.org/10.64898/2026.04.27.720955; this version posted April 30, 2026. The copyright holder for this preprint (which was not certified by peer review) is the author/funder, who has granted bioRxiv a license to display the preprint in perpetuity. It is made available under aCC-BY 4.0 International license.

PROOF. If we let $\widetilde{\boldsymbol{\eta}} \in \mathbb{R}^{\sum_i J_i}$ be the stacked vector of the $\boldsymbol{\eta}_i$ and $\widetilde{\mathbf{P}} \in \mathbb{R}^{\sum_i J_i \times \sum_i J_i}$ be the block-diagonal matrix of the $\mathbf{P}_i$, then $\widetilde{\boldsymbol{\eta}} \mid \widetilde{\mathbf{X}} \sim \mathcal{N}\left(\widetilde{\mathbf{X}}\boldsymbol{\beta}, \widetilde{\mathbf{P}}^{-1}\right)$. We may then apply Theorem 1. That is, $\widetilde{\mathbf{P}}$ is invertible if all of the $\mathbf{P}_i$ are, and, by assumption, $\widetilde{\mathbf{X}}$ has full column rank. We may repeat our analysis with $\widetilde{\boldsymbol{\eta}}$, $\widetilde{\mathbf{X}}$, and $\widetilde{\mathbf{P}}$ replacing $\boldsymbol{\eta}$, $\mathbf{X}$, and $\mathbf{P}$. We conclude that $\boldsymbol{\beta}$ and $\widetilde{\mathbf{P}}$ are identifiable. As $\widetilde{\mathbf{P}}$ is block-diagonal with blocks $\mathbf{P}_i$, it follows that the $\mathbf{P}_i$ are identifiable. □

We now consider the lattice models. In Theorem 1, we have shown that there must be a unique precision matrix, so it remains to show that only one set of parameters could generate a given $\mathbf{P}$.

THEOREM 3. *In addition to the assumptions in Theorem 1, assume that:*

1. *(CAR, SAR) The fixed and known adjacency matrix* $\mathbf{W}$ *(and hence the degree matrix* $\mathbf{D}$*) has no rows or columns that are identically zero.*
2. *(Leroux) The fixed and known adjacency matrix* $\mathbf{W}$ *has at least one non-zero entry.*
3. *The value of* τ^2 *is strictly greater than zero.*
4. *The value of* γ *is such that the precision matrix* $\mathbf{P}$ *is invertible and positive-definite.*

Then, we have that the parameters γ *and* τ^2 *in the CAR, SAR, and Leroux models are identifiable.*

The first condition is necessary for the precision matrix $\mathbf{P}$ to be invertible in the CAR and SAR models. The second condition is a somewhat trivial technical condition that allows the Leroux model to have spatial correlation (otherwise the covariance would be diagonal and totally non-spatial). The third and fourth conditions apply to all three lattice models and ensure that the distribution of $\boldsymbol{\eta}$ is proper (i.e., integrates to 1).

S4.1. *Conclusions for the Lattice Models.* From Theorems 1 and 3 and Corollary 2, we conclude that the parameters $(\boldsymbol{\beta}, \tau_i^2, \gamma_i)$ are identifiable in the lattice models under the set of mild assumptions specified above. Numerically, we see in our simulations that all spatial covariance parameters indeed appear to be identifiable and that $\boldsymbol{\beta}$ is identifiable in all models.

S4.2. *The spNNGP Model.* For the spNNGP model, we draw upon the Gaussian process literature and note that the kernel parameters $\boldsymbol{\psi}_i$ are generally not identifiable (Arendt, Apley and Chen, 2012; Brynjarsdóttir and O'Hagan, 2014), though recent work has discovered some conditions under which identification is possible (Plumlee and Joseph, 2018; Kim and Lee, 2021; Tang, Zhang and Banerjee, 2021; Chen et al., 2023). However, Theorem 1 still applies, and thus $\boldsymbol{\beta}$ is still identifiable in this setting.

S5. Identifiability Proofs.

S5.1. *Proof of Theorem 1.*

PROOF. We consider two sets of parameter values for the regression coefficient $\boldsymbol{\beta}$ and the precision matrix $\mathbf{P}$, namely $\boldsymbol{\beta}_1$ and $\boldsymbol{\beta}_2 \in \mathbb{R}^J$ and $\mathbf{P}_1$ and $\mathbf{P}_2 \in \mathbb{R}^{J\times J}$. The log-likelihood of $\boldsymbol{\eta}$ given $\mathbf{X}$ is given by

$$\frac{-J}{2}\log(2\pi) + \frac{1}{2}\log\det\mathbf{P} - \frac{1}{2}(\boldsymbol{\eta} - \mathbf{X}\boldsymbol{\beta})^\top \mathbf{P}(\boldsymbol{\eta} - \mathbf{X}\boldsymbol{\beta}). \tag{31}$$

bioRxiv preprint doi: https://doi.org/10.64898/2026.04.27.720955; this version posted April 30, 2026. The copyright holder for this preprint (which was not certified by peer review) is the author/funder, who has granted bioRxiv a license to display the preprint in perpetuity. It is made available under aCC-BY 4.0 International license.

If we take the difference between the log-likelihoods at the two sets of parameter values and set it to zero, we find (after dropping a common factor of $1/2$):

$$\begin{aligned} 0 = &\boldsymbol{\eta}^\top (\mathbf{P}_2 - \mathbf{P}_1)\,\boldsymbol{\eta} \\ &- \boldsymbol{\eta}^\top [2\,(\mathbf{P}_2\,\mathbf{X}\,\boldsymbol{\beta}_2 - \mathbf{P}_1\,\mathbf{X}\,\boldsymbol{\beta}_1)] \\ &+ \left(-\boldsymbol{\beta}_1^\top \mathbf{X}^\top \mathbf{P}_1\,\mathbf{X}\,\boldsymbol{\beta}_1 + \boldsymbol{\beta}_2^\top \mathbf{X}^\top \mathbf{P}_2\,\mathbf{X}\,\boldsymbol{\beta}_2\right) + \log \frac{\det \mathbf{P}_1}{\det \mathbf{P}_2}. \end{aligned} \tag{32}$$

Importantly, Equation (32) must hold for all $\boldsymbol{\eta}$ and has the same form as Equation (42) in Lemma 5. We may apply the Lemma and conclude that:

$$\mathbf{P}_2 - \mathbf{P}_1 = 0, \tag{33a}$$

$$\mathbf{P}_1\,\mathbf{X}\,\boldsymbol{\beta}_1 - \mathbf{P}_2\,\mathbf{X}\,\boldsymbol{\beta}_2 = \mathbf{0}, \tag{33b}$$

and,

$$\left(-\boldsymbol{\beta}_1^\top \mathbf{X}^\top \mathbf{P}_1\,\mathbf{X}\,\boldsymbol{\beta}_1 + \boldsymbol{\beta}_2^\top \mathbf{X}^\top \mathbf{P}_2\,\mathbf{X}\,\boldsymbol{\beta}_2\right) + \log \frac{\det \mathbf{P}_1}{\det \mathbf{P}_2} = 0. \tag{33c}$$

Via Equations (33a) and (33b), we conclude that $\mathbf{P}_1 = \mathbf{P}_2$, so that

$$\mathbf{P}_1\,\mathbf{X}\,\boldsymbol{\beta}_1 - \mathbf{P}_2\,\mathbf{X}\,\boldsymbol{\beta}_2 = \mathbf{P}_1\,\mathbf{X}(\boldsymbol{\beta}_1 - \boldsymbol{\beta}_2) = \mathbf{0}\,.$$

By assumption, $\mathbf{P}_1$ and $\mathbf{X}$ have full column rank, so that the nullspace of $\mathbf{P}_1\,\mathbf{X}$ is only the zero vector. It follows that $\boldsymbol{\beta}_1 - \boldsymbol{\beta}_2 = \mathbf{0}$. □

S5.2. *Proof of Theorem 3.* We separate the proof into three parts, corresponding to each of the three lattice models. For each part, we consider two sets of parameters: γ_1 and γ_2, and τ_1^2 and τ_2^2. We consider the explicit forms of the precision matrices $\mathbf{P}_1 = \mathbf{P}_2$ and argue that the parameters must be identical.

S5.2.1. *CAR.*

PROOF. For a CAR model, we may write

$$\mathbf{P}_1 = \frac{1}{\tau_1^2}\,(\mathbf{D} - \gamma_1\,\mathbf{W})\,,$$

and similarly for $\mathbf{P}_2$. As $\mathbf{W}$ has no zero rows or columns, there exists a non-zero off-diagonal entry of $\mathbf{W}$, denoted $W_{j,j'}$. The corresponding entry of $\mathbf{P}_1$ has the form $-(\gamma_1/\tau_1^2)W_{j,j'}$ and that for $\mathbf{P}_2$ has the form $-(\gamma_2/\tau_2^2)W_{j,j'}$ For these two values to be equal, we must have $\gamma_1/\tau_1^2 = \gamma_2/\tau_2^2$. Now, we consider and equate a given diagonal entry of both precision matrices:

$$\frac{1}{\tau_1^2} D_{j,j} = \frac{1}{\tau_2^2} D_{j,j}.$$

Since by assumption $D_{j,j}$ is strictly positive, the only way for this statement to hold is if $\tau_1^2 = \tau_2^2$. Finally, if $\tau_1^2 = \tau_2^2$ and $\gamma_1/\tau_1^2 = \gamma_2/\tau_2^2$, we can conclude that $\gamma_1 = \gamma_2$. □

bioRxiv preprint doi: https://doi.org/10.64898/2026.04.27.720955; this version posted April 30, 2026. The copyright holder for this preprint (which was not certified by peer review) is the author/funder, who has granted bioRxiv a license to display the preprint in perpetuity. It is made available under aCC-BY 4.0 International license.

S5.2.2. *Leroux.*

PROOF. For a Leroux model, we may write

$$\mathbf{P}_1 = \frac{1}{\tau_1^2}\left[\gamma_1(\mathbf{D}-\mathbf{W}) + (1-\gamma_1)\,\mathbf{I}\right],$$

and similarly for $\mathbf{P}_2$. By assumption, there is at least one non-zero off-diagonal element of $\mathbf{W}$, denoted $W_{j,j'}$. The corresponding entry of $\mathbf{P}_1$ has the form $-(\gamma_1/\tau_1^2)W_{j,j'}$ and that for $\mathbf{P}_2$ has the form $-(\gamma_2/\tau_2^2)W_{j,j'}$ For these two values to be equal, we must have $\gamma_1/\tau_1^2 = \gamma_2/\tau_2^2$. Now, considering and equating the diagonal entries of $\mathbf{P}_1$ and $\mathbf{P}_2$ yields

$$\frac{\gamma_1}{\tau_1^2}D_{j,j} + \frac{1-\gamma_1}{\tau_1^2} = \frac{\gamma_2}{\tau_2^2}D_{j,j} + \frac{1-\gamma_2}{\tau_2^2}.$$

Simplifying and using the fact that $\gamma_1/\tau_1^2 = \gamma_2/\tau_2^2$ yields that $\tau_1^2 = \tau_2^2$. Finally, if $\tau_1^2 = \tau_2^2$ and $\gamma_1/\tau_1^2 = \gamma_2/\tau_2^2$, we can conclude that $\gamma_1 = \gamma_2$. □

S5.2.3. *SAR.*

PROOF. For a SAR model, we may write

$$\mathbf{P}_1 = \frac{1}{\tau_1^2}\left[\mathbf{I} - \gamma_1\,\mathbf{D}^{-1}\,\mathbf{W}\right]^\top \mathbf{D}\left[\mathbf{I} - \gamma_1\,\mathbf{D}^{-1}\,\mathbf{W}\right],$$

and similarly for $\mathbf{P}_2$. Expanding this definition and simplifying, we may write

$$\mathbf{P}_1 = \frac{1}{\tau_1^2}\mathbf{D} - 2\frac{\gamma_1}{\tau_1^2}\mathbf{W} + \frac{\gamma_1^2}{\tau_1^2}\mathbf{W}\,\mathbf{D}^{-1}\,\mathbf{W}.$$

For $\mathbf{P}_1 - \mathbf{P}_2 = 0$ to hold, we require

$$0 = \left(\frac{1}{\tau_1^2} - \frac{1}{\tau_2^2}\right)\mathbf{D} + 2\left(\frac{\gamma_2}{\tau_2^2} - \frac{\gamma_1}{\tau_1^2}\right)\mathbf{W} + \left(\frac{\gamma_1^2}{\tau_1^2} - \frac{\gamma_2^2}{\tau_2^2}\right)\mathbf{W}\,\mathbf{D}^{-1}\,\mathbf{W}.$$

Equivalently, defining $\mathbf{A} = \mathbf{D}^{-1}\,\mathbf{W}$ and multiplying from the left with $\mathbf{D}^{-1}$ yields

$$0 = \left(\frac{1}{\tau_1^2} - \frac{1}{\tau_2^2}\right)\mathbf{I} + 2\left(\frac{\gamma_2}{\tau_2^2} - \frac{\gamma_1}{\tau_1^2}\right)\mathbf{A} + \left(\frac{\gamma_1^2}{\tau_1^2} - \frac{\gamma_2^2}{\tau_2^2}\right)\mathbf{A}^2. \tag{34}$$

Equation (34) says that a polynomial of degree two annihilates $\mathbf{A}$. Immediately, we see that if $\mathbf{A}$ has more than two distinct eigenvalues, all coefficients of this polynomial must be zero, as then a polynomial of degree two cannot annihilate $\mathbf{A}$ since all of the eigenvalues of $\mathbf{A}$ must be roots of any annihilating polynomial of $\mathbf{A}$ (see Hoffmann and Kunze (1971, Sec. 6.3) for the mathematical background). We would then conclude that every coefficient of Equation (34) is zero, so that $\tau_1^2 = \tau_2^2$ and $\gamma_1 = \gamma_2$.

If $\mathbf{A}$ has two or fewer distinct eigenvalues, the analysis is more complicated. Since $\mathbf{A}$ is a stochastic matrix (the rows sum to 1 and the entries are non-negative), we know the eigenvalues to be less than or equal to 1 in magnitude by the Gershgorin Circle Theorem—see Landau and Odlyzko (1981) for a discussion of the eigenvalues of matrices like $\mathbf{A}$. Moreover, since $\mathbf{A}$ is stochastic, the leading eigenvalue is 1—consider that the vector of all 1s is an eigenvector with eigenvalue 1. We consider three cases for the eigenvalues of $\mathbf{A}$: two distinct eigenvalues that have different magnitudes (1 and $|\lambda| \neq 1$), two distinct eigenvalues that have the same magnitude (necessarily ± 1), and a single distinct eigenvalue (necessarily 1). In all three cases, we will assume that Equation (34) is not identically zero and derive a contradiction.

bioRxiv preprint doi: https://doi.org/10.64898/2026.04.27.720955; this version posted April 30, 2026. The copyright holder for this preprint (which was not certified by peer review) is the author/funder, who has granted bioRxiv a license to display the preprint in perpetuity. It is made available under aCC-BY 4.0 International license.

For the first case, assume that $\mathbf{A}$ has two distinct eigenvalues that have different magnitudes. To achieve this, we require both $\frac{\gamma_1^2}{\tau_1^2} \neq \frac{\gamma_2^2}{\tau_2^2}$ and $\frac{\gamma_1}{\tau_1^2} \neq \frac{\gamma_2}{\tau_2^2}$ so that the linear and quadratic terms in Equation (34) are non-zero. There are then two possibilities for the eigenvalues:

$$\frac{\left(\gamma_1\tau_2^2 - \gamma_2\tau_1^2\right) \pm \sqrt{\tau_1^2\tau_2^2}\,|\gamma_1 - \gamma_2|}{\gamma_1^2\tau_2^2 - \gamma_2^2\tau_1^2}, \tag{35}$$

where we have applied the quadratic formula to Equation (34). The largest of the two eigenvalues, corresponding to the addition, must be 1. Setting Equation (35) to 1 and solving yields the solution

$$\tau_2^2 = \frac{(1-\gamma_2)^2}{(1-\gamma_1)^2}\tau_1^2. \tag{36}$$

This solution holds for $0 < \gamma_1 < \gamma_2 < 1$ and for $-1 < \gamma_1 \leq 0$ with $\gamma_2 \neq \frac{\gamma_1}{2\gamma_1 - 1}$. As long as $\gamma_1 \neq \gamma_2$ or $\gamma_2 \neq \frac{\gamma_1}{2\gamma_1-1}$ for $\gamma_1 \neq 1/2$, the constraint that $\frac{\gamma_1^2}{\tau_1^2} \neq \frac{\gamma_2^2}{\tau_2^2}$ holds. We see that with this choice of τ_2^2, the other root has value

$$\frac{2 - \gamma_1 - \gamma_2}{\gamma_1 + \gamma_2 - 2\gamma_1\gamma_2},$$

where we have substituted Equation (36) into (35) and taken the negative root. For some eigenvalue $\lambda \neq \pm 1$ of $\mathbf{A}$, we can solve for γ_2 in terms of γ_1 and λ, where we find

$$\gamma_2 = \frac{\gamma_1(1+\lambda) - 2}{(2\gamma - 1)\lambda - 1}.$$

However, γ_2 must be less than 1, which only holds for $-1 < \gamma_1 < 0$ and $-1 < \lambda < \frac{1}{2\gamma_1 - 1}$. But γ_2 must also be greater than -1, which only holds either for $-1 < \gamma_1 < 0$ and $\frac{1}{2\gamma_1-1} < \lambda < 1$ or for $0 < \gamma_1 < 1$ and $|\lambda| < 1$. That is, we cannot simultaneously bound γ_2 from above and below. We conclude that there is no pair of distinct parameters that lead to a valid solution, or, that the SAR model is identifiable in this setting.

For the second case, assume that $\mathbf{A}$ has two distinct eigenvalues with the same magnitude. Since $\mathbf{A}$ is a stochastic matrix, these values must be ± 1. Looking at the quadratic formula, we require $\frac{\gamma_1^2}{\tau_1^2} \neq \frac{\gamma_2^2}{\tau_2^2}$ but that $\frac{\gamma_1}{\tau_1^2} = \frac{\gamma_2}{\tau_2^2}$ to have roots of identical magnitude and opposite sign. We would then conclude that the non-zero eigenvalues of $\mathbf{A}$ must be

$$\pm\sqrt{\frac{\tau_2^2 - \tau_1^2}{\gamma_2^2\tau_1^2 - \gamma_1^2\tau_2^2}}. \tag{37}$$

We find that setting

$$\tau_2^2 = \frac{(1-\gamma_2^2)\tau_1^2}{1-\gamma_1^2}$$

leads to an eigenvalue of 1. To satisfy the constraint $\frac{\gamma_1}{\tau_1^2} = \frac{\gamma_2}{\tau_2^2}$, we find that we would need $\gamma_1 = \gamma_2$, but then $\tau_2^2 = \tau_1^2$—a contradiction to our assumption. Hence, if $\mathbf{A}$ has eigenvalues ± 1, the SAR model is identifiable.

For the third case, assume that $\mathbf{A}$ has only a single distinct eigenvalue. As $\mathbf{A}$ is a stochastic matrix, this eigenvalue is 1. If we assume that the quadratic term in the polynomial in $\mathbf{A}$ is identically zero, or, that $\frac{\gamma_1^2}{\tau_1^2} = \frac{\gamma_2^2}{\tau_2^2}$ but that $\frac{\gamma_1}{\tau_1^2} \neq \frac{\gamma_2}{\tau_2^2}$, we would find that a linear polynomial annihilates $\mathbf{A}$. That is, $\mathbf{A}$ would be diagonalizable (as the minimal polynomial has no repeated roots) and have eigenvalues equal to 1. However, the only such matrix is the identity matrix: $\mathbf{W}$

bioRxiv preprint doi: https://doi.org/10.64898/2026.04.27.720955; this version posted April 30, 2026. The copyright holder for this preprint (which was not certified by peer review) is the author/funder, who has granted bioRxiv a license to display the preprint in perpetuity. It is made available under aCC-BY 4.0 International license.

and hence $\mathbf{A}$ have zeros along the diagonal, so we cannot have a linear polynomial annihilate $\mathbf{A}$. So, we then must assume that Equation (34) is proportional to $(\mathbf{A}-\mathbf{I})^2$. If this condition holds, the quadratic and constant coefficients must be equal and the quadratic coefficient must be $-1/2$ times the linear coefficient:

$$\frac{\gamma_1^2}{\tau_1^2}-\frac{\gamma_2^2}{\tau_2^2}=\frac{1}{\tau_1^2}-\frac{1}{\tau_2^2} \text{ and } \frac{\gamma_1^2}{\tau_1^2}-\frac{\gamma_2^2}{\tau_2^2}=\frac{\gamma_1}{\tau_1^2}-\frac{\gamma_2}{\tau_2^2}.$$

For both equations to hold, we would need $\gamma_1=\gamma_2=1$, but then Equation (34) would only have a non-zero constant term—a contradiction that would lead to $\tau_1^2=\tau_2^2$. If we took $\gamma_1=\gamma_2\neq 1$, we would find that we needed $\tau_1^2=\tau_2^2$—a contradiction once again as Equation (34) is now identically zero. We conclude that this third case is also not possible and that the SAR model is identifiable in this setting. □

S6. Useful Results.

S6.1. *Useful Identities.*

1. For a random variable $\mathbf{X}$ with mean $\mathbb{E}[\mathbf{X}]=\boldsymbol{\mu}$ and covariance matrix $\mathrm{Var}[\mathbf{X}]=\boldsymbol{\Sigma}$ and a constant matrix $\mathbf{A}$,

$$\mathbb{E}[\mathbf{X}^\top\mathbf{A}\,\mathbf{X}]=\mu^\top\mathbf{A}\,\mu+\mathrm{Tr}[\mathbf{A}\,\boldsymbol{\Sigma}]. \tag{38}$$

2. For matrices $\mathbf{X}$ and $\mathbf{Y}$ and a scalar t, we have (Dattorro, 2010, D.2.4)

$$\frac{d}{dt}\log\det[\mathbf{X}+t\,\mathbf{Y}]=\mathrm{Tr}[(\mathbf{X}+t\,\mathbf{Y})^{-1}\,\mathbf{Y}]. \tag{39}$$

3. For a matrix $\mathbf{A}\in\mathbb{R}^{p\times p}$, vectors $\mathbf{b}$ and $\mathbf{x}\in\mathbb{R}^p$, and a scalar c,

$$\nabla_{\mathbf{x}}(\mathbf{x}^\top\mathbf{A}\,\mathbf{x}+\mathbf{b}^\top\mathbf{x}+c)=(\mathbf{A}+\mathbf{A}^\top)\,\mathbf{x}+\mathbf{b}\,. \tag{40}$$

S6.2. *Useful Definitions.*

DEFINITION 4 (Moran's I). For a vector $\mathbf{z}\in\mathbb{R}^J$ with entries z_j, let $\bar{z}=\sum_j z_j/J$. The Moran's I is defined as

$$I=\frac{J}{W}\frac{\sum_{j=1}^J\sum_{j'=1}^J w_{j,j'}(z_j-\bar{z})(z_{j'}-\bar{z})}{\sum_{j=1}^J(z_j-\bar{z})^2}, \tag{41}$$

where the $w_{j,j'}$ are spatial weights, with $w_{j,j}=0$ and $W=\sum_{j,j'}w_{j,j'}$ (Cressie, 2015, Sec. 6.5.1).

For a lattice model, the entries of the adjacency matrix $\mathbf{W}$ can be used in (41), or, if coordinates are available, the inverse of the Euclidean distance between pairs of points is another common choice.

S6.3. *Useful Lemmas.*

LEMMA 5. *If for a square, symmetric matrix $\mathbf{A}\in\mathbb{R}^{J\times J}$, a vector $\mathbf{b}\in\mathbb{R}^J$, and a scalar c we have that*

$$\mathbf{x}^\top\mathbf{A}\,\mathbf{x}+\mathbf{b}^\top\mathbf{x}+c=0 \tag{42}$$

for all vectors $\mathbf{x}\in\mathbb{R}^J$, then we must have that all of $\mathbf{A}$, $\mathbf{b}$, and c are zero.

bioRxiv preprint doi: https://doi.org/10.64898/2026.04.27.720955; this version posted April 30, 2026. The copyright holder for this preprint (which was not certified by peer review) is the author/funder, who has granted bioRxiv a license to display the preprint in perpetuity. It is made available under aCC-BY 4.0 International license.

PROOF. If Equation (42) holds for all $\mathbf{x}$, then the gradient must also be identically zero for all $\mathbf{x}$. Taking the gradient in $\mathbf{x}$, we find that

$$2\,\mathbf{A}^\top\mathbf{x}+\mathbf{b} = 2\,\mathbf{A}\,\mathbf{x}+\mathbf{b} = \mathbf{0}$$

or that

$$\mathbf{A}\,\mathbf{x} = -\frac{1}{2}\,\mathbf{b}$$

for all $\mathbf{x}$. Assuming $\mathbf{A}$ is not identically zero, then there must exist a non-zero $\mathbf{x}$ that is not in the nullspace of $\mathbf{A}$. Then, for any scalar α, we have that

$$\mathbf{A}(\alpha\,\mathbf{x}) = -\frac{\alpha}{2}\,\mathbf{b},$$

but we also know that, by assumption,

$$\mathbf{A}(\alpha\,\mathbf{x}) = -\frac{1}{2}\,\mathbf{b}\,.$$

Thus, we must have

$$-\frac{\alpha}{2}\,\mathbf{b} = -\frac{1}{2}\,\mathbf{b}$$

for all $\alpha \in \mathbb{R}$, so that $\mathbf{b}$ must be identically zero. But if $\mathbf{b}$ is identically zero, we have that $\mathbf{A}\,\mathbf{x} = 0$ for all $\mathbf{x}$. If this holds, we must have that the nullspace of $\mathbf{A}$ is all of $\mathbb{R}^J$, so that $\mathbf{A}$ is entirely zero, which is a contradiction of our assumption that $\mathbf{A}$ is not identically zero. Then, taking $\mathbf{A}$ to be the zero matrix, we must have that

$$\mathbf{A}\,\mathbf{x} = \mathbf{0} = -\frac{1}{2}\,\mathbf{b},$$

or that $\mathbf{b}$ must be identically zero. Returning to Equation (42), if $\mathbf{A}$ and $\mathbf{b}$ are zero, we require $c = 0$. □

bioRxiv preprint doi: https://doi.org/10.64898/2026.04.27.720955; this version posted April 30, 2026. The copyright holder for this preprint (which was not certified by peer review) is the author/funder, who has granted bioRxiv a license to display the preprint in perpetuity. It is made available under aCC-BY 4.0 International license.

S7. Supplementary Figures.

S7.1. *Synthetic Data: Method Runtime.*

bioRxiv preprint doi: https://doi.org/10.64898/2026.04.27.720955; this version posted April 30, 2026. The copyright holder for this preprint (which was not certified by peer review) is the author/funder, who has granted bioRxiv a license to display the preprint in perpetuity. It is made available under aCC-BY 4.0 International license.

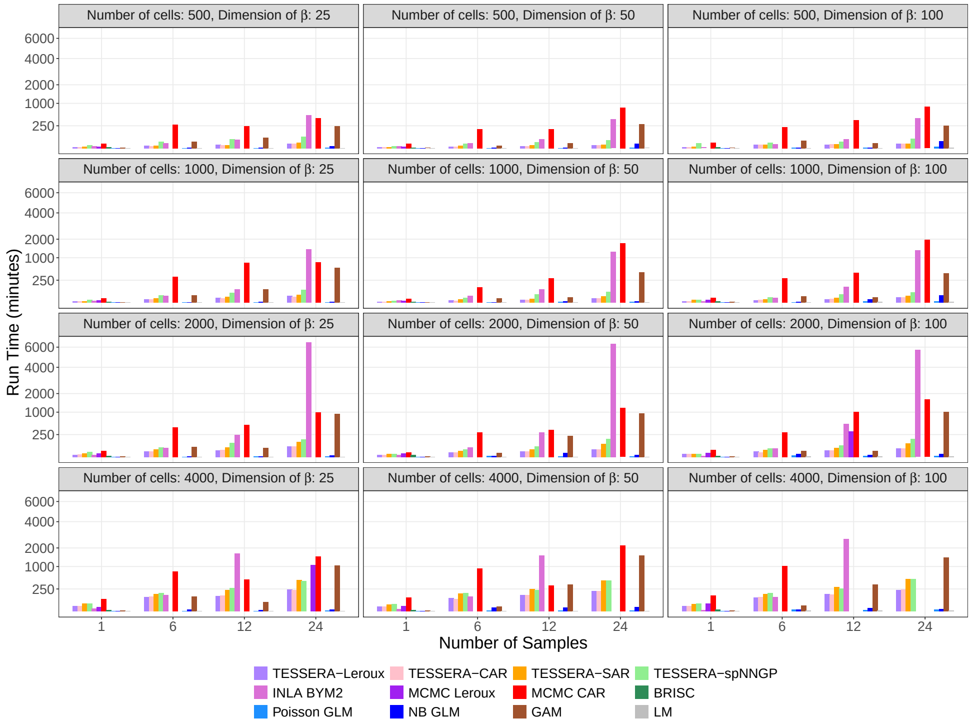

Figure S-1: **Computational scalability of TESSERA across different simulation scenarios.** We evaluated method run time by simulating Poisson-Leroux-distributed data for two conditions with balanced sample sizes and varying numbers of cell types within condition. This simulation was designed exclusively to assess computational scalability of model fitting; it was not used for hypothesis testing or evaluating statistical power. We systematically varied the number of samples ($n \in \{1, 6, 12, 24\}$, where $n = 1$ represents a single-sample baseline and $n > 1$ represents two balanced groups of $n/2$ samples each), the number of cells per sample (J_i ranging from 500 to 4,000), and the fixed-effect dimensionality ($\dim(\boldsymbol{\beta}) \in \{25, 50, 100\}$). For $n = 1$, $\boldsymbol{\beta}$ consists solely of cell type effects; otherwise, it incorporates disease, sample, and cell type effects. The number of cell types is then chosen so as to achieve a given $\dim(\boldsymbol{\beta})$. Spatial parameters were derived from empirical fits to the IGHG2 gene across 14 individual samples from a real-world kidney dataset (Abedini et al., 2024). Specifically, we obtained 14 pairs of spatial covariance parameters (γ, τ^2) from these fits. For each simulation, we selected covariance parameters by sampling at random, with replacement from this pool of 14 empirical pairs to achieve the desired sample size. Fixed effects $\boldsymbol{\beta}$ were drawn from a normal distribution matching the empirical mean and variance of the entries of the estimated $\boldsymbol{\beta}$ corresponding to each condition. Cell coordinates were sampled uniformly in the unit square and distance-thresholded to maintain an average of five neighbors per cell. Each simulation was run on a single thread of a 2.4 GHz Intel Xeon processor with 22 GB of RAM. Reported run times represent the average across five independent simulation trials. TESSERA was implemented with a 30-iteration limit, a threshold typically sufficient for convergence on the real-world data; implementation details for the compared methods are provided in Table 2. Facets represent unique combinations of cell count and fixed-effect dimensionality. Missing bars indicate simulation scenarios where a method (e.g., INLA or MCMC samplers) failed to terminate due to convergence issues, memory constraints, or time-limit exhaustion.

bioRxiv preprint doi: https://doi.org/10.64898/2026.04.27.720955; this version posted April 30, 2026. The copyright holder for this preprint (which was not certified by peer review) is the author/funder, who has granted bioRxiv a license to display the preprint in perpetuity. It is made available under aCC-BY 4.0 International license.

S7.2. *Synthetic Data: Comparison of* `TESSERA` *with Multi-Sample Methods.*

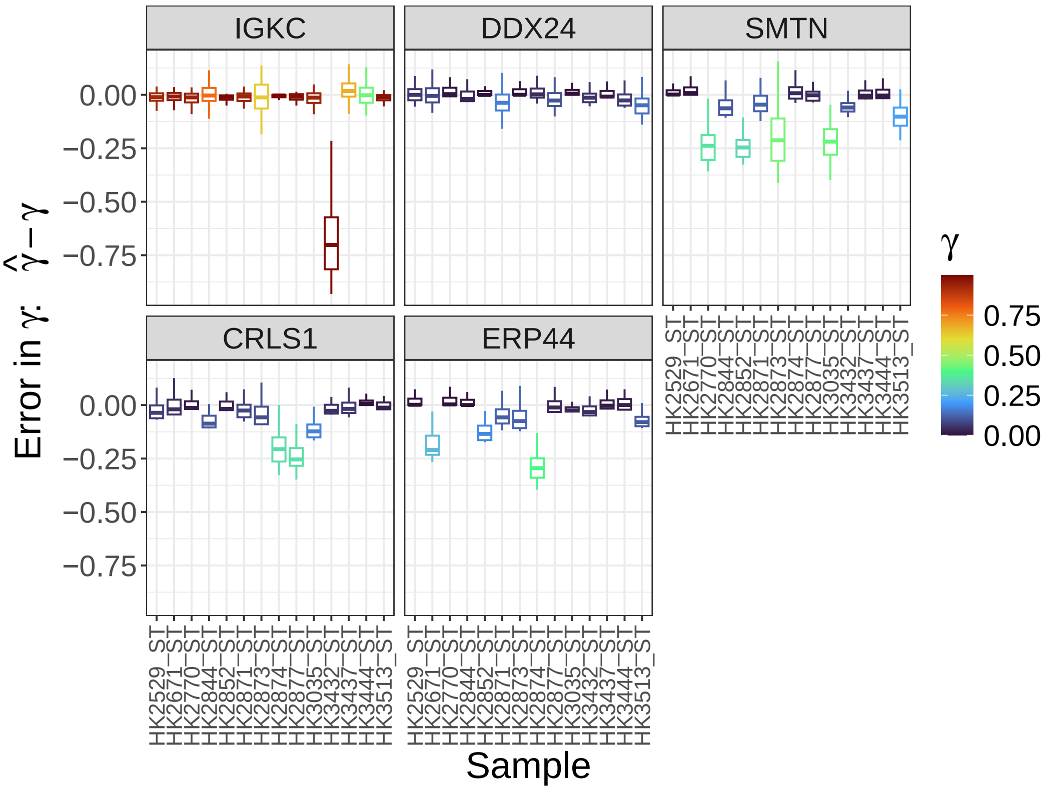

Figure S-2: **Error in estimating the sample-specific spatial covariance parameter** γ**.** To evaluate how effectively TESSERA estimates the sample-specific spatial covariance parameter $\gamma \in [0, 1)$, we examine the estimation error $\widehat{\gamma} - \gamma$ across 100 independent simulation trials. Synthetic data were generated from a Poisson-Leroux distribution with generative parameters estimated from the multi-sample kidney dataset (Abedini et al., 2024), for a representative subset of five genes selected based on their raw count variances across all cells and samples: *IGKC* (maximum variance), *DDX24* (third quartile), *SMTN* (median), *CRLS1* (first quartile), and *ERP44* (minimum). Each facet corresponds to a given gene, and facets are ordered row-wise and top-to-bottom by decreasing expression variance. Within each facet, each boxplot summarizes the estimation error over 100 trials for each of the 14 kidney samples and is color-coded by the true generative γ value used in the simulation (see legend). TESSERA fit with a Poisson-Leroux model yields consistently low estimation error for γ across a selection of genes exhibiting a wide range of variances in expression measures (with the exception of one sample for the highly variable gene *IGKC*).

bioRxiv preprint doi: https://doi.org/10.64898/2026.04.27.720955; this version posted April 30, 2026. The copyright holder for this preprint (which was not certified by peer review) is the author/funder, who has granted bioRxiv a license to display the preprint in perpetuity. It is made available under aCC-BY 4.0 International license.

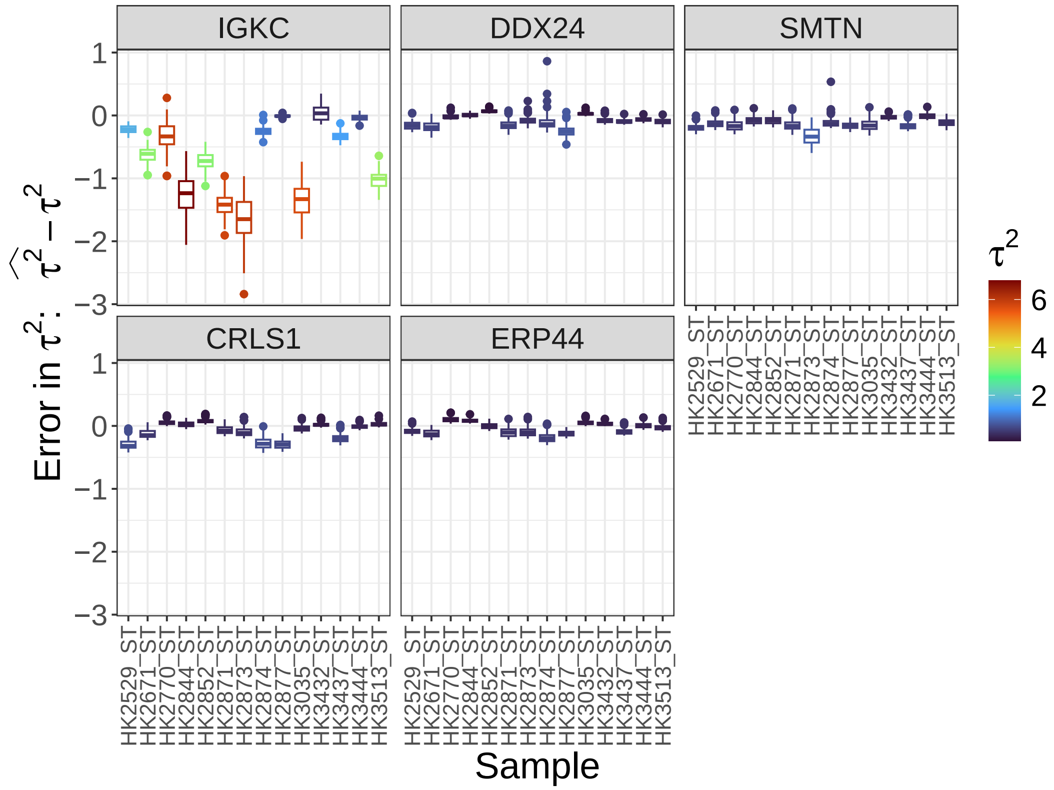

Figure S-3: **Error in estimating the sample-specific spatial covariance parameter τ^2.** To evaluate how effectively TESSERA estimates the sample-specific spatial covariance parameter $\tau^2 \in (0, \infty)$, we examine the estimation error $\widehat{\tau^2} - \tau^2$ across 100 independent simulation trials. Synthetic data were generated from a Poisson-Leroux distribution with generative parameters estimated from the multi-sample kidney dataset (Abedini et al., 2024), for a representative subset of five genes selected based on their raw count variances across all cells and samples: *IGKC* (maximum variance), *DDX24* (third quartile), *SMTN* (median), *CRLS1* (first quartile), and *ERP44* (minimum). Each facet corresponds to a given gene, and facets are ordered row-wise and top-to-bottom by decreasing expression variance. Within each facet, each boxplot summarizes the estimation error over 100 trials for each of the 14 kidney samples and is color-coded by the true generative τ^2 value used in the simulation (see legend). TESSERA fit with a Poisson-Leroux model yields consistently low estimation error for τ^2 across a selection of genes exhibiting a wide range of variances in expression measures. Note that although the absolute estimation error is higher for *IGKC* than for the other genes, this gene also has higher true parameter value for τ^2. Thus, the absolute error remains small relative to the magnitude of the parameter.

bioRxiv preprint doi: https://doi.org/10.64898/2026.04.27.720955; this version posted April 30, 2026. The copyright holder for this preprint (which was not certified by peer review) is the author/funder, who has granted bioRxiv a license to display the preprint in perpetuity. It is made available under aCC-BY 4.0 International license.

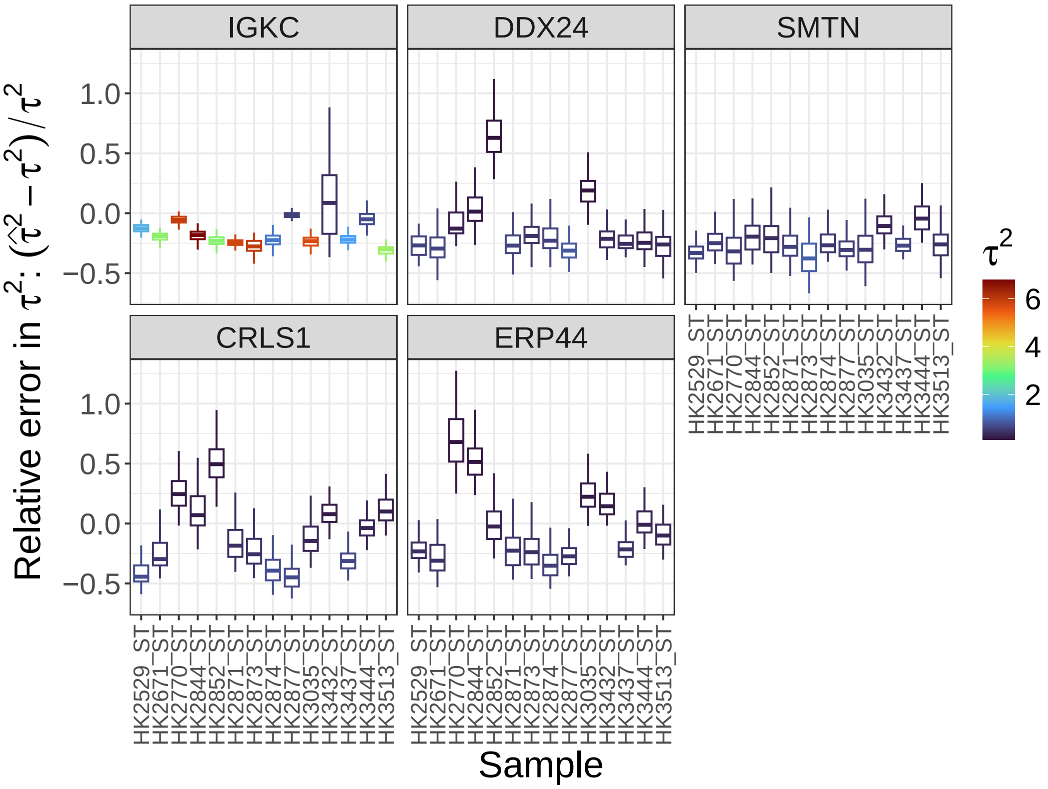

Figure S-4: **Error in estimating the sample-specific spatial covariance parameter τ^2.** To evaluate how effectively TESSERA estimates the sample-specific spatial covariance parameter $\tau^2 \in (0, \infty)$, we examine the relative estimation error $(\widehat{\tau^2} - \tau^2)/\tau^2$ across 100 independent simulation trials. Synthetic data were generated from a Poisson-Leroux distribution with generative parameters estimated from the multi-sample kidney dataset (Abedini et al., 2024), for a representative subset of five genes selected based on their raw count variances across all cells and samples: *IGKC* (maximum variance), *DDX24* (third quartile), *SMTN* (median), *CRLS1* (first quartile), and *ERP44* (minimum). Each facet corresponds to a given gene, and facets are ordered row-wise and top-to-bottom by decreasing expression variance. Within each facet, each boxplot summarizes the estimation error over 100 trials for each of the 14 kidney samples and is color-coded by the true generative τ^2 value used in the simulation (see legend).

bioRxiv preprint doi: https://doi.org/10.64898/2026.04.27.720955; this version posted April 30, 2026. The copyright holder for this preprint (which was not certified by peer review) is the author/funder, who has granted bioRxiv a license to display the preprint in perpetuity. It is made available under aCC-BY 4.0 International license.

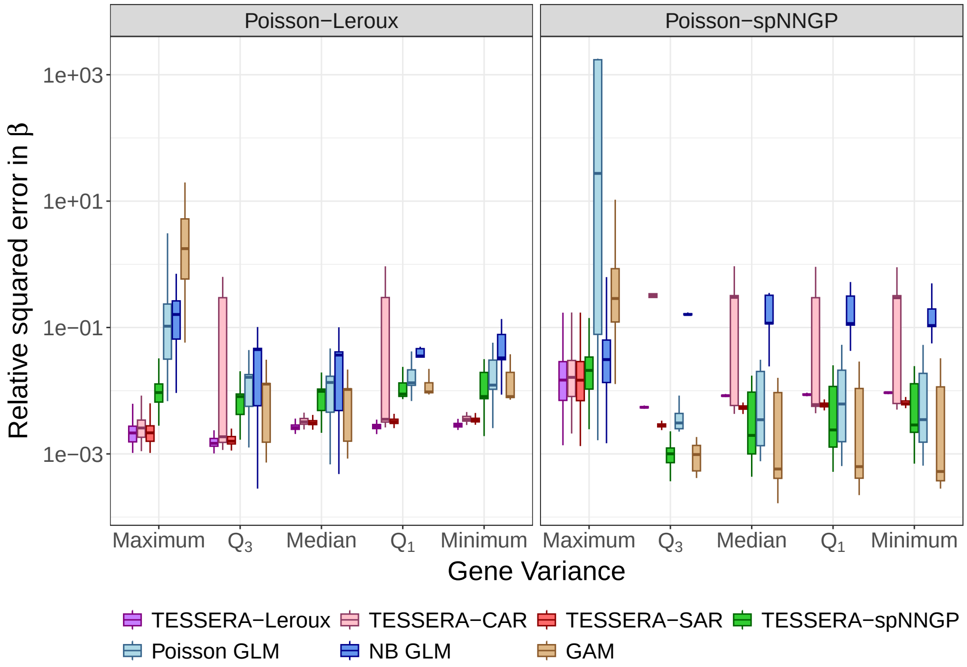

Figure S-5: **Relative squared error in estimating the shared fixed effect β.** Synthetic data were generated using Poisson-Leroux and Poisson-spNNGP distributions, with generative parameters estimated from a multi-sample real-world kidney dataset (Abedini et al., 2024). We compared TESSERA fit with lattice and spNNGP models against a Poisson GLM, an NB GLM, and a GAM. Estimation accuracy of the fixed effects β was assessed via the relative squared error, $\| \beta - \widehat{\beta} \|_2^2 / \| \beta \|_2^2$, calculated across 100 independent simulation trials. Performance is displayed for a representative subset of genes covering the full distribution of observed expression variance; specifically, genes were selected corresponding to the maximum (*IGKC*), third quartile (*DDX24*), median (*SMTN*), first quartile (*CRLS1*), and minimum (*ERP44*) values of raw count variance across all cells and samples. Facets represent the underlying data-generating models.

bioRxiv preprint doi: https://doi.org/10.64898/2026.04.27.720955; this version posted April 30, 2026. The copyright holder for this preprint (which was not certified by peer review) is the author/funder, who has granted bioRxiv a license to display the preprint in perpetuity. It is made available under aCC-BY 4.0 International license.

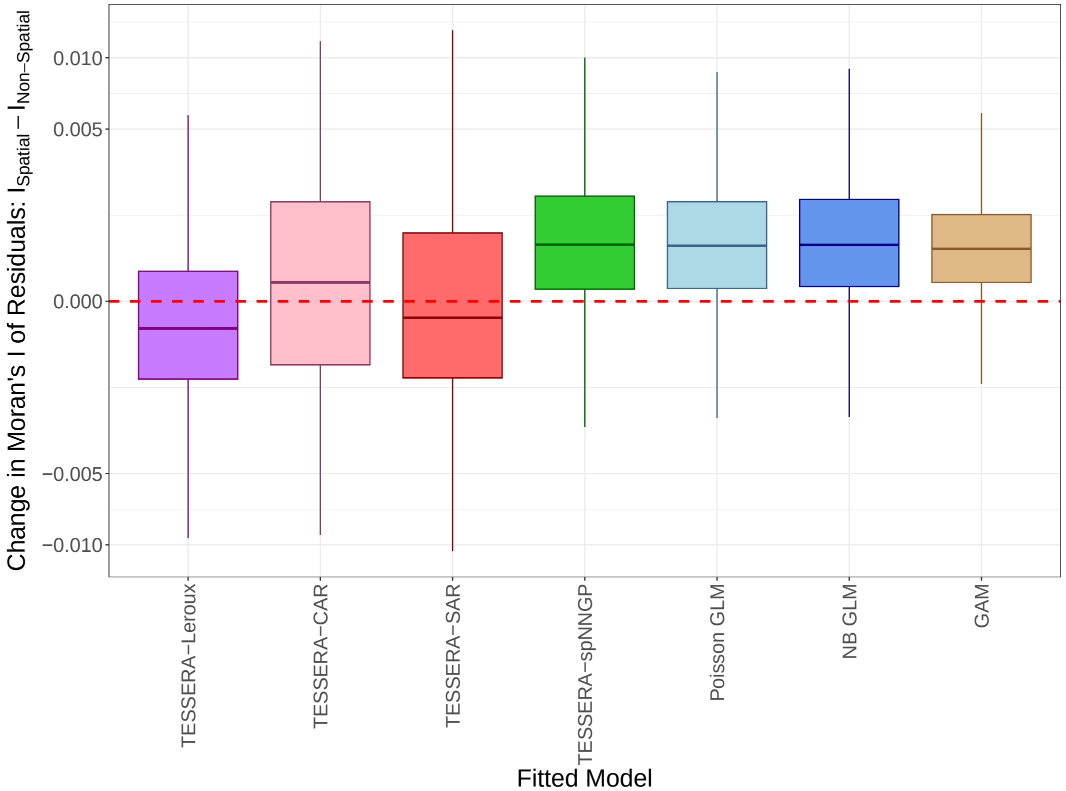

Figure S-6: **Residual spatial autocorrelation across fitted models (Poisson-Leroux-generated data).** To evaluate how effectively each model accounts for spatial autocorrelation, we examine the change in the Moran's I of residuals between spatial and non-spatial generative settings ($I_{Spatial} - I_{Non-Spatial}$). This differencing accounts for the varying baseline capacities of models to reduce autocorrelation due to general model flexibility and complexity. The boxplots summarize differences in Moran's I over 100 simulation trials, for each of seven methods: TESSERA (fit with lattice and spNNGP models), a Poisson GLM, an NB GLM, and a GAM. Values at or below zero (red dashed line) indicate that the method effectively accounts for the spatial autocorrelation present in the data. To generate these results, we produced 100 independent simulation trials for each of two synthetic datasets from a Poisson-Leroux distribution with generative parameters estimated from a multi-sample real-world kidney dataset (Abedini et al., 2024). To calculate $I_{Spatial}$, we use the parameters as fitted; for $I_{Non-Spatial}$, we set the spatial correlation parameter γ to zero so that the generated counts are independent.

bioRxiv preprint doi: https://doi.org/10.64898/2026.04.27.720955; this version posted April 30, 2026. The copyright holder for this preprint (which was not certified by peer review) is the author/funder, who has granted bioRxiv a license to display the preprint in perpetuity. It is made available under aCC-BY 4.0 International license.

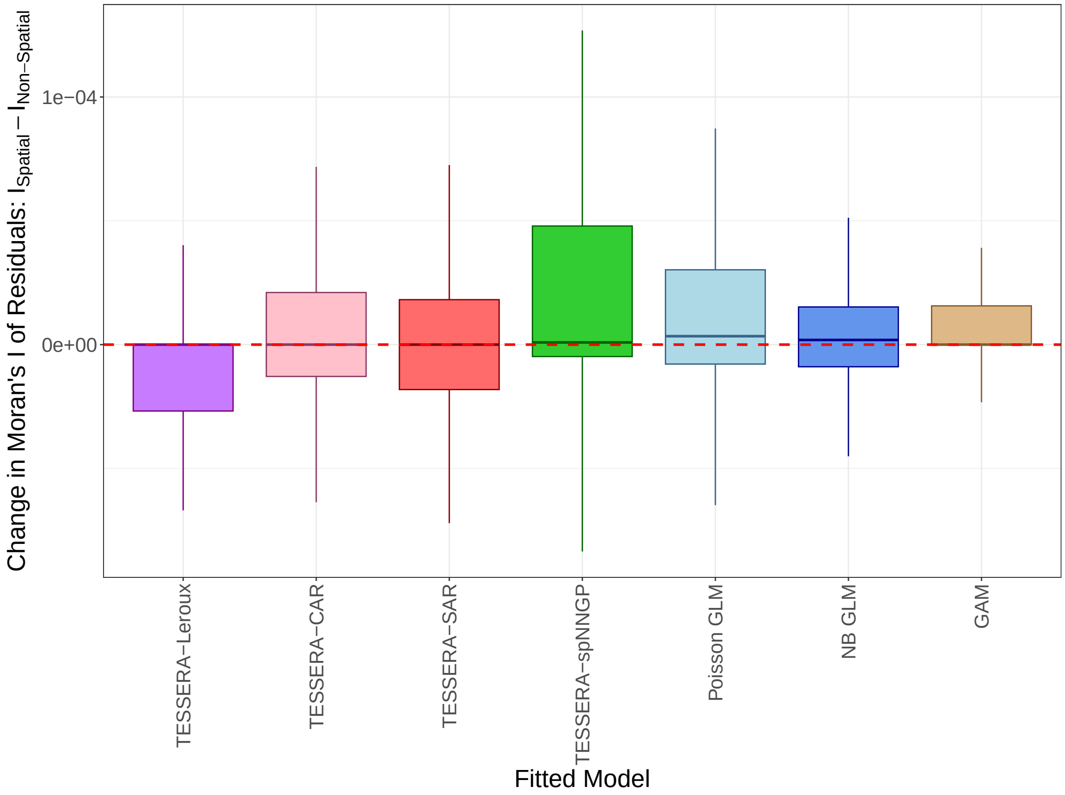

Figure S-7: **Residual spatial autocorrelation across fitted models (Poisson-spNNGP-generated data).** To evaluate how effectively each model accounts for spatial autocorrelation, we examine the change in the Moran's I of residuals between spatial and non-spatial generative settings ($I_{Spatial} - I_{Non-Spatial}$). This differencing accounts for the varying baseline capacities of models to reduce autocorrelation due to general model flexibility and complexity. The boxplots summarize differences in Moran's I over 100 simulation trials, for each of seven methods: TESSERA (fit with lattice and spNNGP models), a Poisson GLM, an NB GLM, and a GAM. Values at or below zero (red dashed line) indicate that the model effectively accounts for the spatial autocorrelation present in the data. To generate these results, we produced 100 independent simulation trials for each of two synthetic datasets from a Poisson-spNNGP distribution with generative parameters estimated from a multi-sample real-world kidney dataset (Abedini et al., 2024). To calculate $I_{Spatial}$, we use the parameters as fitted; for $I_{Non-Spatial}$, we set the spatial variance parameter (the partial sill) to zero so that the generated counts are independent.

bioRxiv preprint doi: https://doi.org/10.64898/2026.04.27.720955; this version posted April 30, 2026. The copyright holder for this preprint (which was not certified by peer review) is the author/funder, who has granted bioRxiv a license to display the preprint in perpetuity. It is made available under aCC-BY 4.0 International license.

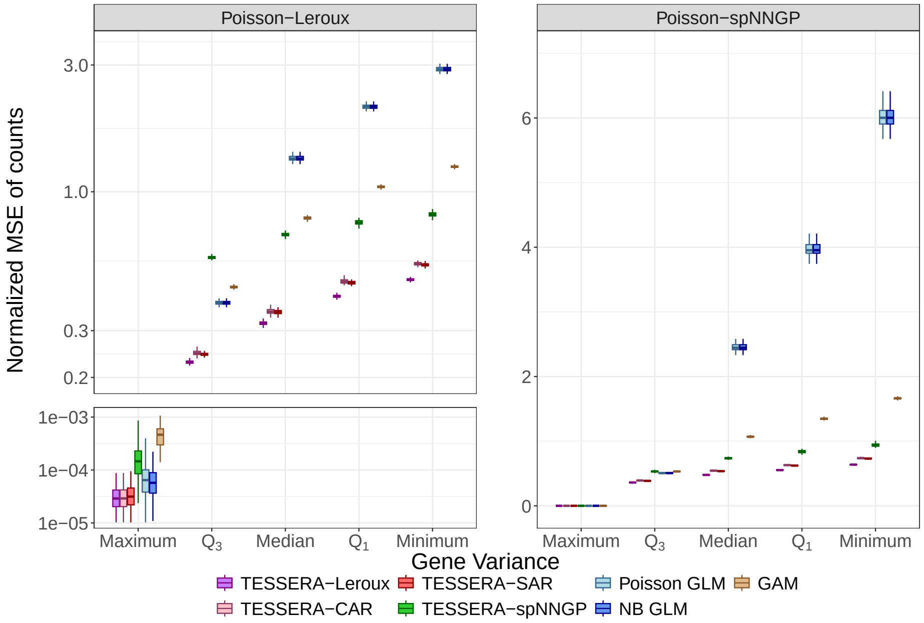

Figure S-8: **Normalized mean squared error for fitted counts $\widehat{\mathbf{Z}}$.** Synthetic data were generated using Poisson-Leroux and Poisson-spNNGP distributions, with generative parameters estimated from a multi-sample real-world kidney dataset (Abedini et al., 2024). We compared TESSERA fit with lattice and spNNGP models against a Poisson GLM, an NB GLM, and a GAM. The boxplots summarize the normalized mean squared error (MSE) for the fitted counts, $(\sum_{i,j}(Z_{i,j} - \widehat{Z}_{i,j})^2/\sum_i J_i)/(\sum_{i,j} Z_{i,j}^2/\sum_i J_i)$, for each method across 100 independent simulation trials. Performance is displayed for a representative subset of genes covering the full distribution of observed expression variance; specifically, genes were selected corresponding to the maximum (*IGKC*), third quartile (*DDX24*), median (*SMTN*), first quartile (*CRLS1*), and minimum (*ERP44*) values of raw count variance across all cells and samples. Facets represent the underlying data-generating models.

bioRxiv preprint doi: https://doi.org/10.64898/2026.04.27.720955; this version posted April 30, 2026. The copyright holder for this preprint (which was not certified by peer review) is the author/funder, who has granted bioRxiv a license to display the preprint in perpetuity. It is made available under aCC-BY 4.0 International license.

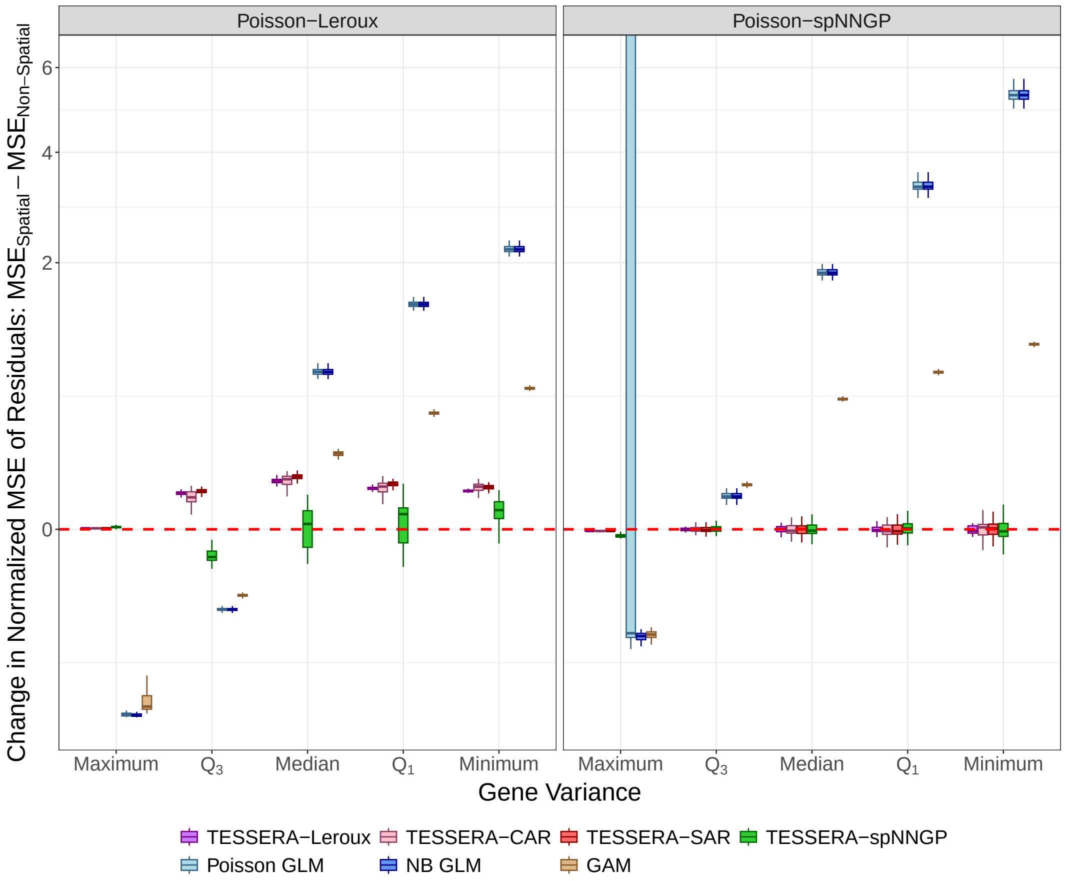

Figure S-9: **Change in normalized mean squared error for fitted counts $\widehat{\mathbf{Z}}$.** To evaluate how effectively each model accounts for spatial autocorrelation, we examine the change in the normalized MSE of the fitted counts between spatial and non-spatial generative settings ($MSE_{Spatial} - MSE_{Non-Spatial}$). This differencing accounts for the varying baseline capacities of models to reduce normalized MSE due to general model flexibility and complexity. The boxplots summarize differences in normalized MSE over 100 simulation trials, for each of seven methods: `TESSERA` (fit with lattice and spNNGP models), a Poisson GLM, an NB GLM, and a GAM and for each of five representative genes covering the full distribution of observed expression variance. Specifically, genes were selected corresponding to the maximum (*IGKC*), third quartile (*DDX24*), median (*SMTN*), first quartile (*CRLS1*), and minimum (*ERP44*) values of raw count variance across all cells and samples. Values at or close to zero (red dashed line) indicate that the model is equally effective in both settings. To generate these results, we produced 100 independent simulation trials for each of two synthetic datasets from either a Poisson-Leroux or Poisson-spNNGP distribution with generative parameters estimated from a multi-sample real-world kidney dataset (Abedini et al., 2024). To calculate $MSE_{Spatial}$, we use the parameters as fitted; for $MSE_{Non-Spatial}$, we set the spatial component (γ for lattice models or the partial sill for an spNNGP model) to zero to ensure the generated counts are independent.

bioRxiv preprint doi: https://doi.org/10.64898/2026.04.27.720955; this version posted April 30, 2026. The copyright holder for this preprint (which was not certified by peer review) is the author/funder, who has granted bioRxiv a license to display the preprint in perpetuity. It is made available under aCC-BY 4.0 International license.

S7.3. *Synthetic Data: Comparison of* `TESSERA` *with Single-Sample Methods.*

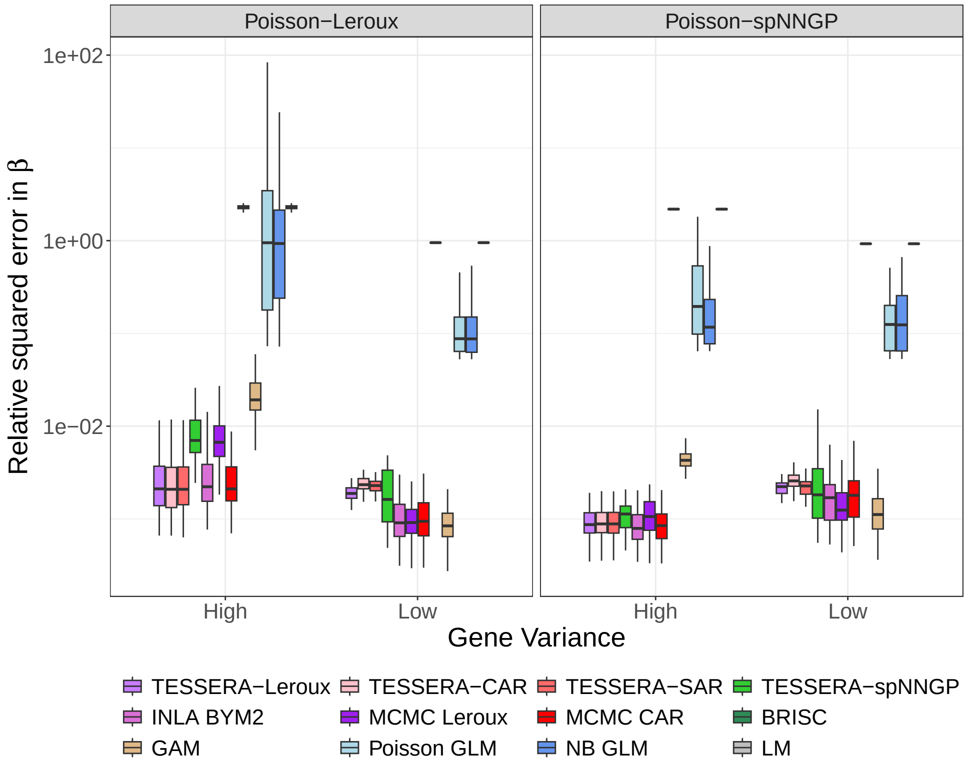

Figure S-10: **Relative squared error in β in single-sample setting (excluding low-abundance cell types).** Synthetic data were generated using Poisson-Leroux and Poisson-spNNGP distributions with respective generative parameters estimated on a multi-sample real-world kidney dataset (Abedini et al., 2024). Estimation methods were evaluated on a single sample (corresponding to the *HK3035_ST* sample from the kidney dataset). Low-abundance cell types (those with ≤ 3 cells) were **excluded** in this set of simulations. We compared `TESSERA` fit with lattice and spNNGP models against existing spatial methods (MCMC-based Leroux/CAR, INLA BYM2, and BRISC), with a Poisson GLM, an NB GLM, a GAM, and a linear model (LM) serving as baselines. Estimation accuracy of the fixed effects β was assessed via the relative squared error, $\|\beta - \widehat{\beta}\|_2^2 / \|\beta\|_2^2$, calculated across 100 independent simulation trials. Performance is shown for genes with the maximum and minimum variances of the raw counts across all cells and samples (denoted by 'High' and 'Low', respectively). Facets represent the underlying data-generating models.

bioRxiv preprint doi: https://doi.org/10.64898/2026.04.27.720955; this version posted April 30, 2026. The copyright holder for this preprint (which was not certified by peer review) is the author/funder, who has granted bioRxiv a license to display the preprint in perpetuity. It is made available under aCC-BY 4.0 International license.

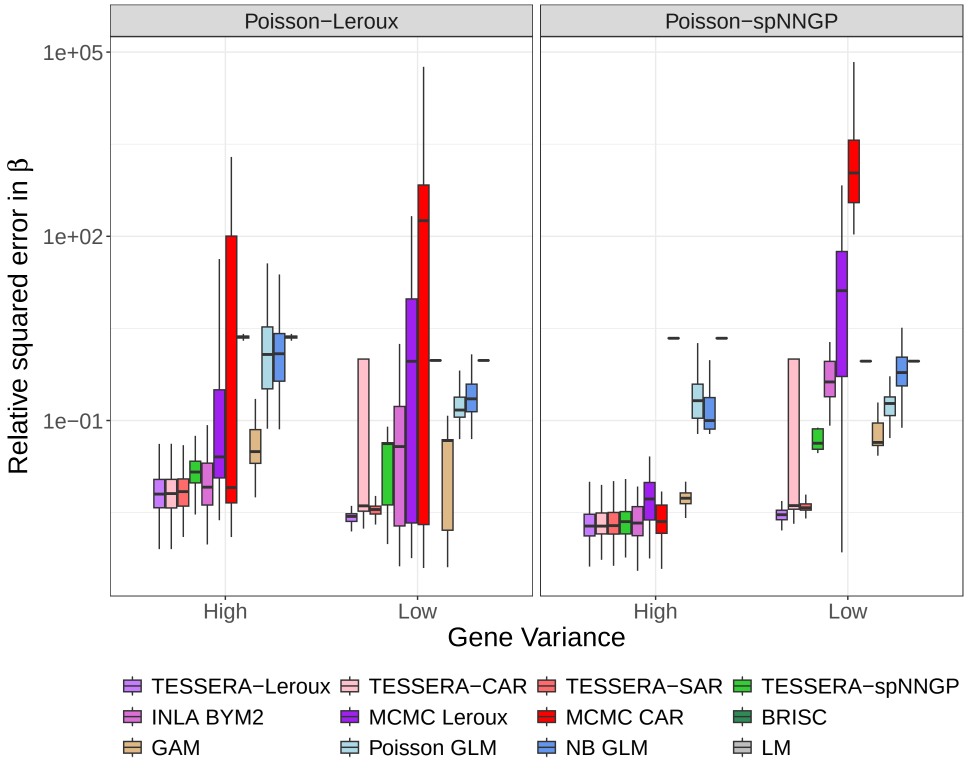

Figure S-11: **Relative squared error in β in single-sample setting (including low-abundance cell types).** Synthetic data were generated using Poisson-Leroux and Poisson-spNNGP distributions, with generative parameters estimated from a multi-sample real-world kidney dataset (Abedini et al., 2024). Estimation methods were evaluated on a single sample (corresponding to sample *HK3035_ST* from the kidney dataset), where low-abundance cell types (≤ 3 cells) were **included** to assess model robustness to small cell groups representing high-variance elements of $\boldsymbol{\beta}$. We compared `TESSERA` with lattice and spNNGP models against existing spatial methods (MCMC-based Leroux/CAR, INLA BYM2, and BRISC), with a Poisson GLM, an NB GLM, a GAM, and a linear model (LM) serving as baselines. Estimation accuracy of the fixed effects $\boldsymbol{\beta}$ was assessed via the relative squared error, $\|\boldsymbol{\beta}-\widehat{\boldsymbol{\beta}}\|_2^2/\|\boldsymbol{\beta}\|_2^2$, calculated across 100 independent simulation trials. Performance is shown for genes with the maximum and minimum variances of raw counts across all cells and samples (denoted as 'High' and 'Low', respectively). Facets represent the underlying data-generating models.

bioRxiv preprint doi: https://doi.org/10.64898/2026.04.27.720955; this version posted April 30, 2026. The copyright holder for this preprint (which was not certified by peer review) is the author/funder, who has granted bioRxiv a license to display the preprint in perpetuity. It is made available under aCC-BY 4.0 International license.

S7.4. *Synthetic Data: Power Simulation.*

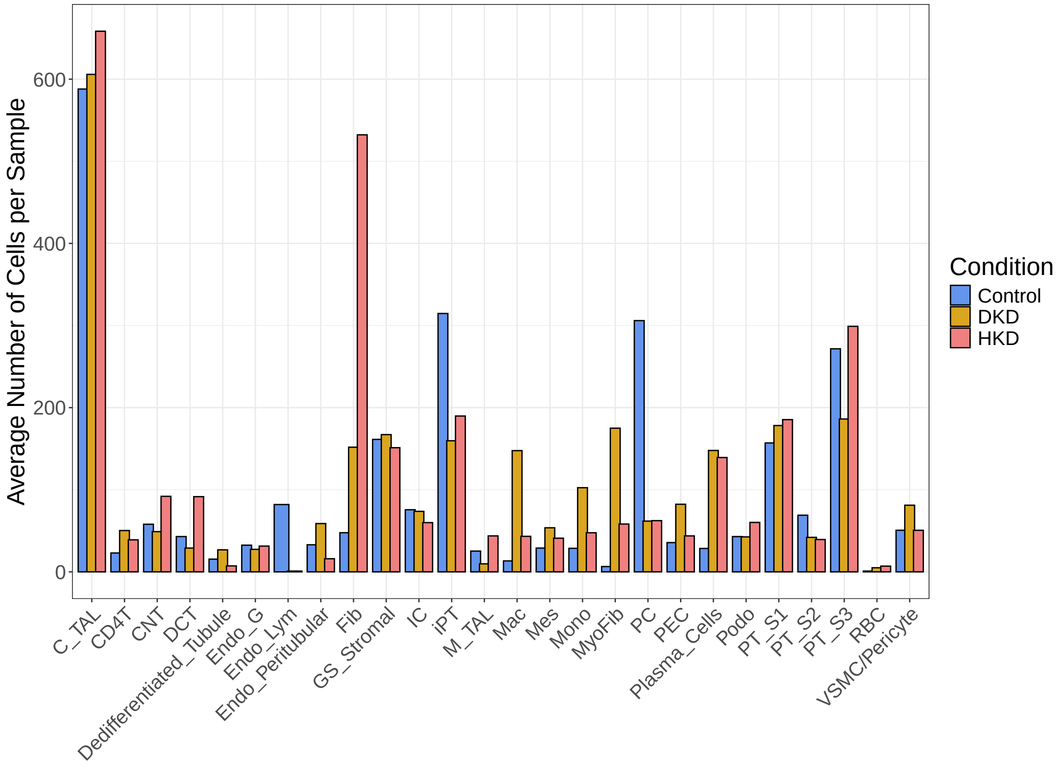

Figure S-12: **Average number of cells per sample by cell type and condition.** Shown are average cell abundance by cell type and condition for a multi-sample real-world kidney dataset (Abedini et al., 2024). Bars represent the mean number of cells per sample for each cell type-condition pair.

bioRxiv preprint doi: https://doi.org/10.64898/2026.04.27.720955; this version posted April 30, 2026. The copyright holder for this preprint (which was not certified by peer review) is the author/funder, who has granted bioRxiv a license to display the preprint in perpetuity. It is made available under aCC-BY 4.0 International license.

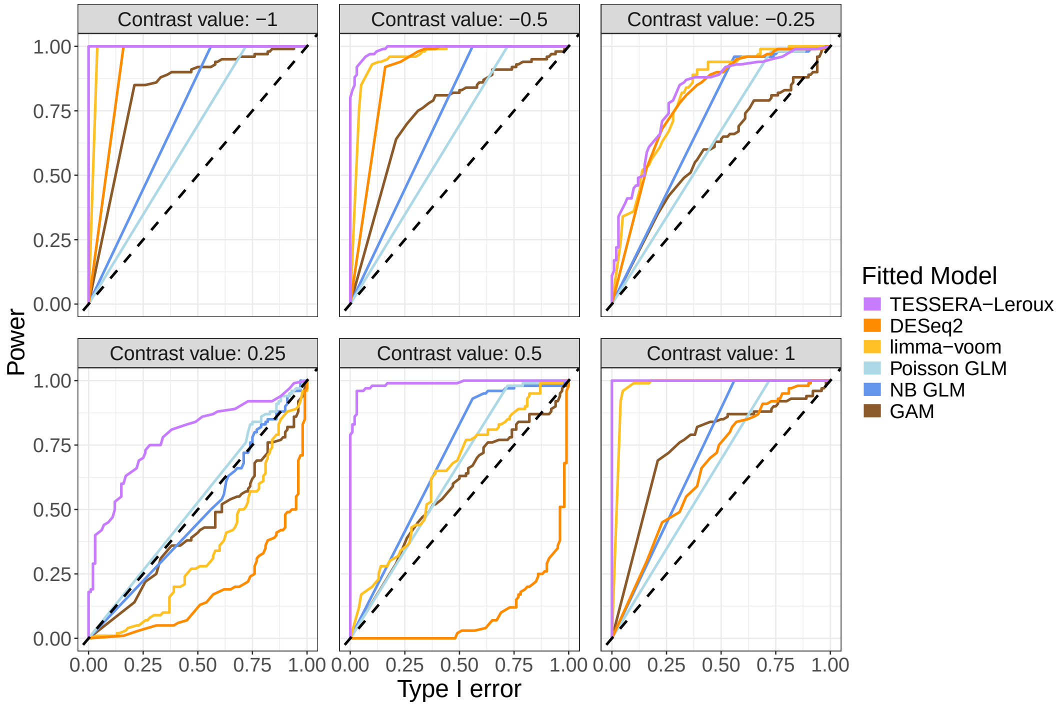

Figure S-13: **ROC curves for Poisson-Leroux-generated data.** This figure evaluates the trade-off between power and Type I error rate for detecting DE between conditions for the *C_TAL* cell type across a range of effect sizes. Synthetic data were generated using a Poisson-Leroux distribution, with generative parameters estimated from the *IGHG2* gene in a multi-sample real-world kidney dataset (Abedini et al., 2024). For each panel shown, we modify two entries of the fixed effects vector $\boldsymbol{\beta}$, corresponding to the Control and the DKD conditions, to generate a contrast of specified size. We set the control coefficient to be the average of all coefficients and the DKD coefficient to be the control coefficient plus a given effect size. We then fit the `TESSERA` Poisson-Leroux model as well as a Poisson GLM, an NB GLM, and a GAM to the data and perform a Wald test to see if the contrast is detectable. For comparison, we also evaluate pseudobulk methods `DESeq2` and `limma-voom`. We present ROC curves of power (y-axis) as a function of the Type I error rate (x-axis) across 100 independent simulation trials for each contrast value. The dotted diagonal line represents the $y = x$ identity line, where the power is exactly equal to the Type I error rate. Curves that rise more steeply toward the top-left corner indicate superior method performance, with `TESSERA` (fit with a Poisson-Leroux model) consistently showing comparable or superior performance to other methods across all contrast values.

bioRxiv preprint doi: https://doi.org/10.64898/2026.04.27.720955; this version posted April 30, 2026. The copyright holder for this preprint (which was not certified by peer review) is the author/funder, who has granted bioRxiv a license to display the preprint in perpetuity. It is made available under aCC-BY 4.0 International license.

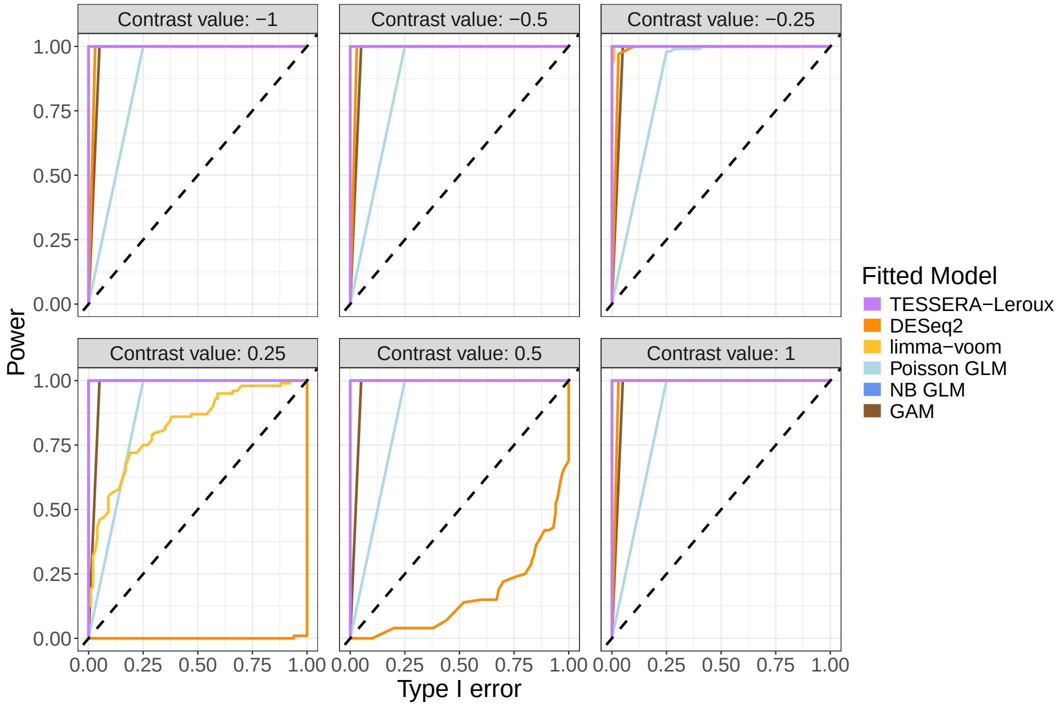

Figure S-14: **ROC curves for Poisson-spNNGP-generated data.** This figure evaluates the trade-off between power and Type I error rate for detecting DE between conditions for the *C_TAL* cell type across a range of effect sizes. Synthetic data were generated using a Poisson-spNNGP distribution, with generative parameters estimated from the *IGHG2* gene in a multi-sample real-world kidney dataset (Abedini et al., 2024). For each panel shown, we modify two entries of the fixed effects vector $\boldsymbol{\beta}$, corresponding to the Control and the DKD conditions, to generate a contrast of specified size. We set the control coefficient to be the average of all coefficients and the DKD coefficient to be the control coefficient plus a given effect size. We then fit the TESSERA Poisson-Leroux model as well as a Poisson GLM, an NB GLM, and a GAM to the data and perform a Wald test to see if the contrast is detectable. For comparison, we also evaluate pseudobulk methods DESeq2 and limma-voom. We present ROC curves of power (y-axis) as a function of the Type I error rate (x-axis) across 100 independent simulation trials for each contrast value. The dotted diagonal line represents the $y = x$ identity line, where the power is exactly equal to the Type I error rate. Curves that rise more steeply toward the top-left corner indicate superior model performance, with TESSERA (fit with a Poisson-Leroux model) consistently showing comparable or superior performance to other methods across all contrast values.

bioRxiv preprint doi: https://doi.org/10.64898/2026.04.27.720955; this version posted April 30, 2026. The copyright holder for this preprint (which was not certified by peer review) is the author/funder, who has granted bioRxiv a license to display the preprint in perpetuity. It is made available under aCC-BY 4.0 International license.

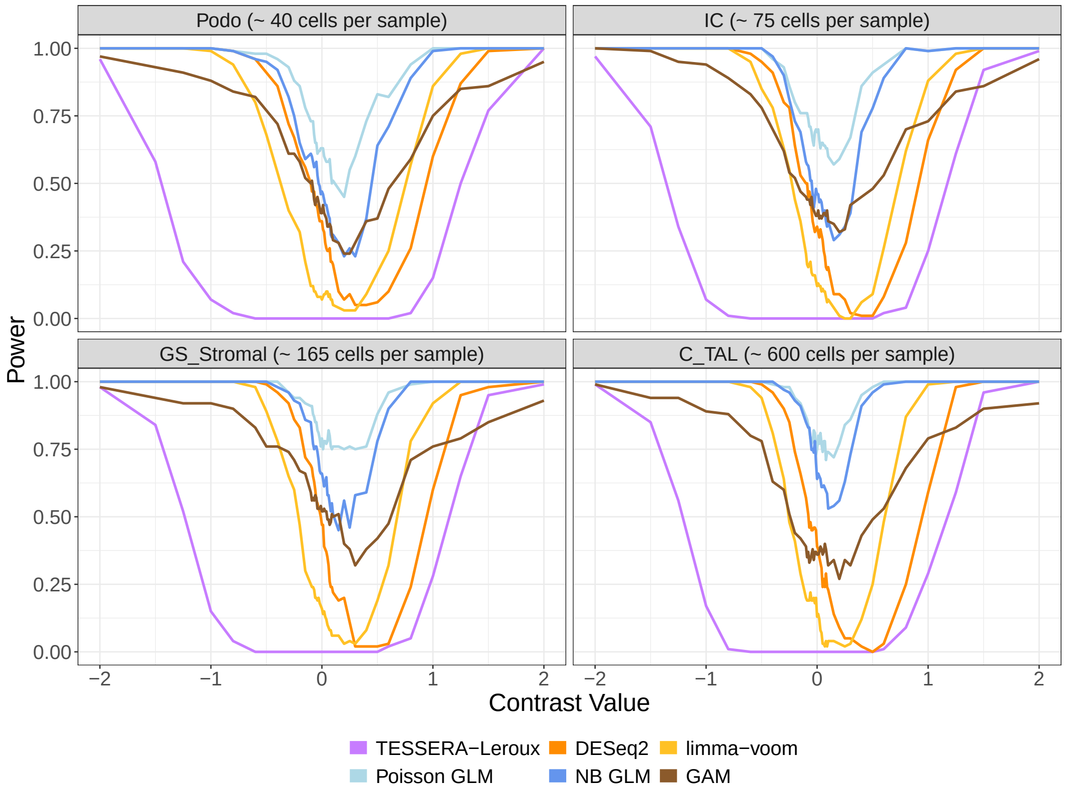

Figure S-15: **Power for Poisson-Leroux-generated data.** This figure examines power for detecting DE between conditions within a cell type for one gene. It presents plots of power (y-axis) as a function of the contrast value (x-axis), where power is estimated by the proportion of 100 independent simulation trials in which the null hypothesis of no DE is rejected at nominal Type I error rate of 0.05. At an effect size of 0, this value represents the Type I error rate. Synthetic data were generated using a Poisson-Leroux distribution, with generative parameters estimated from the *IGHG2* gene in a multi-sample real-world kidney dataset (Abedini et al., 2024). For each cell type shown, we modify two entries of the fixed effects vector β, corresponding to the Control and the DKD conditions, to generate a contrast of specified size. We set the control coefficient to be the average of all coefficients and the DKD coefficient to be the control coefficient plus a given effect size. We then fit the `TESSERA` Poisson-Leroux model as well as a Poisson GLM, an NB GLM, and a GAM to the data and perform a Wald test to see if the contrast is detectable. For comparison, we also evaluate pseudobulk methods `DESeq2` and `limma-voom`.

bioRxiv preprint doi: https://doi.org/10.64898/2026.04.27.720955; this version posted April 30, 2026. The copyright holder for this preprint (which was not certified by peer review) is the author/funder, who has granted bioRxiv a license to display the preprint in perpetuity. It is made available under aCC-BY 4.0 International license.

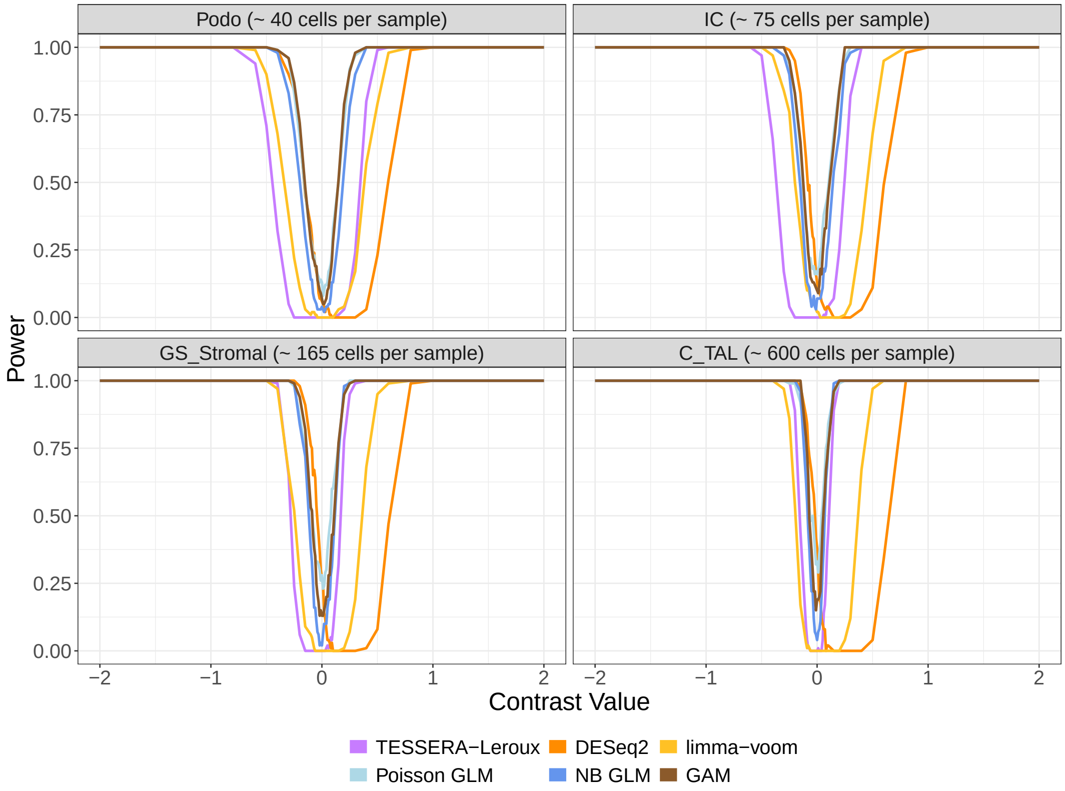

Figure S-16: **Power for Poisson-spNNGP-generated data.** This figure examines power for detecting DE between conditions within a cell type for one gene. It presents plots of power (y-axis) as a function of the contrast value (x-axis), where power is estimated by the proportion of 100 independent simulation trials in which the null hypothesis of no DE is rejected at nominal Type I error rate of 0.05. At an effect size of 0, this value represents the Type I error rate. Synthetic data were generated using a Poisson-spNNGP distribution, with generative parameters estimated from the *IGHG2* gene in a multi-sample real-world kidney dataset (Abedini et al., 2024). For each cell type shown, we modify two entries of the fixed effects vector $\boldsymbol{\beta}$, corresponding to the Control and the DKD conditions to generate a contrast of specified size. We set the control coefficient to be the average of all coefficients and the DKD coefficient to be the control coefficient plus a given effect size. We then fit the TESSERA Poisson-Leroux model as well as a Poisson GLM, an NB GLM, and a GAM to the data and perform a Wald test to see if the contrast is detectable. For comparison, we also evaluate pseudobulk methods DESeq2 and limma-voom.

bioRxiv preprint doi: https://doi.org/10.64898/2026.04.27.720955; this version posted April 30, 2026. The copyright holder for this preprint (which was not certified by peer review) is the author/funder, who has granted bioRxiv a license to display the preprint in perpetuity. It is made available under aCC-BY 4.0 International license.

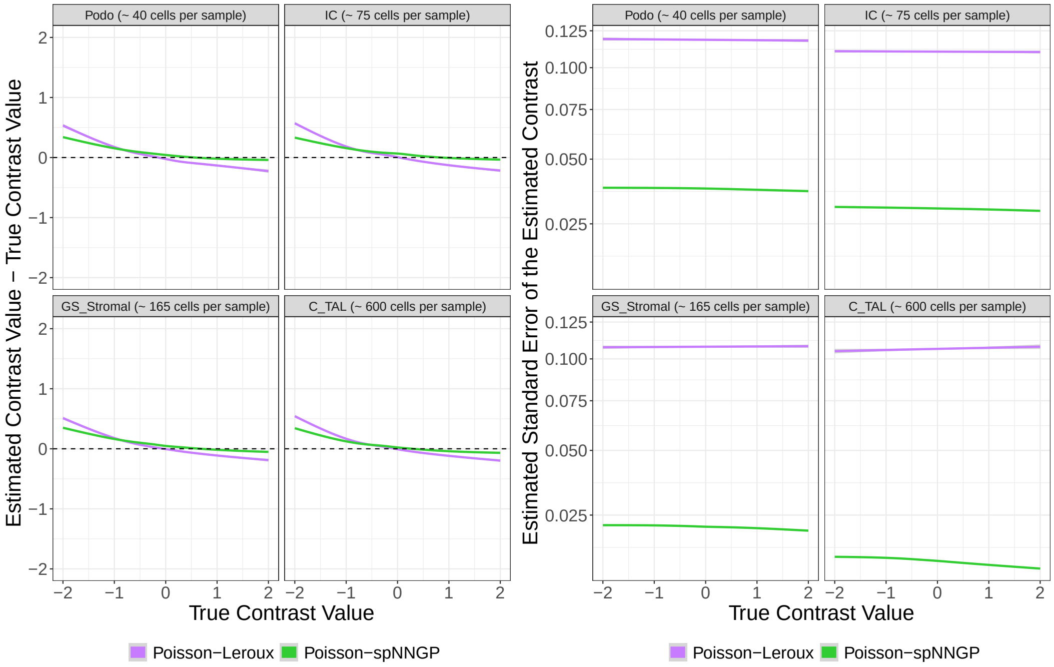

Figure S-17: **Estimated contrast value and standard error of the estimated contrast for Poisson-Leroux and Poisson-spNNGP-generated data.** Synthetic data were generated using a Poisson-Leroux or Poisson-spNNGP distribution, with generative parameters estimated from the *IGHG2* gene in a multi-sample real-world kidney dataset (Abedini et al., 2024). For each cell type shown, we modify two entries of the fixed effects vector β, corresponding to the Control and the DKD conditions to generate a contrast of specified size. We set the control coefficient to be the average of all coefficients and the DKD coefficient to be the control coefficient plus a given effect size. We then fit the `TESSERA` Poisson-Leroux model to the data and perform a Wald test to see if the contrast is detectable. Across 100 independent simulation trials for each contrast and contrast value, we plot performance metrics against the true contrast value. The left-hand plot shows the difference between the average of the estimated contrasts and the true contrast values, while the right-hand plot displays the average estimated standard error of the estimated contrast.

bioRxiv preprint doi: https://doi.org/10.64898/2026.04.27.720955; this version posted April 30, 2026. The copyright holder for this preprint (which was not certified by peer review) is the author/funder, who has granted bioRxiv a license to display the preprint in perpetuity. It is made available under aCC-BY 4.0 International license.

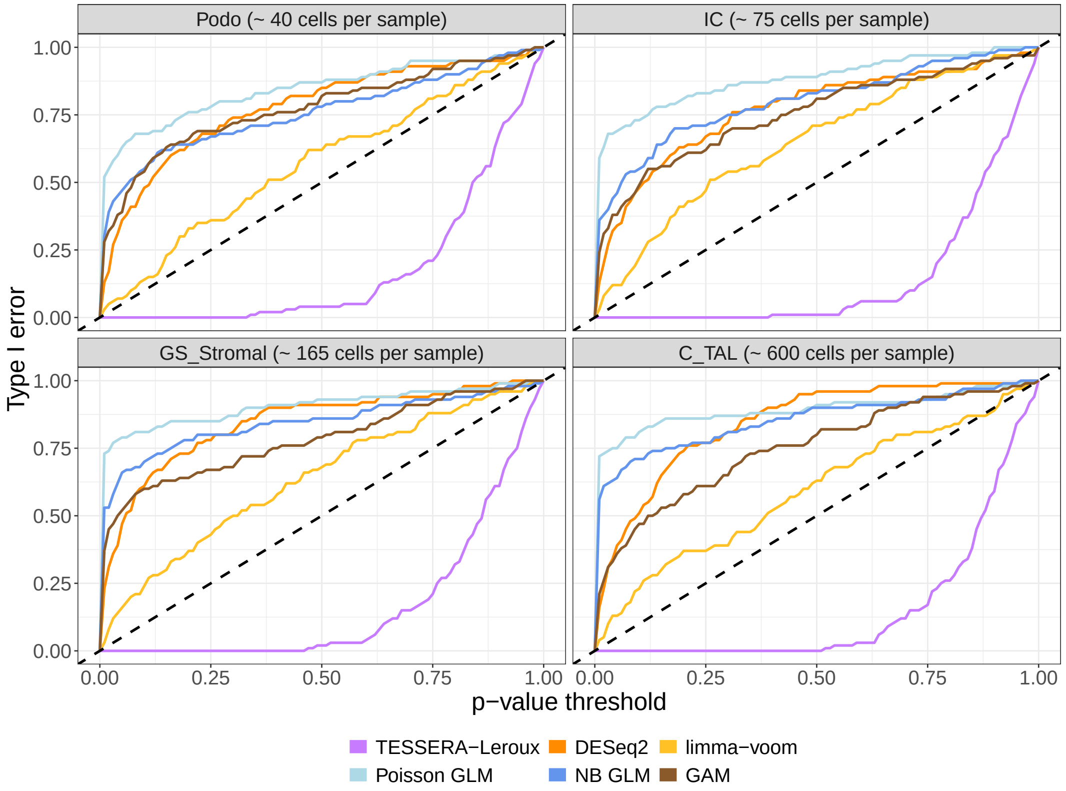

Figure S-18: **Type I error rate for Poisson-Leroux-generated data.** Synthetic data were generated using a Poisson-Leroux distribution, with generative parameters estimated from the *IGHG2* gene in a multi-sample real-world kidney dataset (Abedini et al., 2024). For each cell type shown, we modify two entries of the fixed effects vector $\boldsymbol{\beta}$, corresponding to the Control and the DKD conditions, to generate a contrast of specified size. We set the control coefficient to be the average of all coefficients and the DKD coefficient to be the control coefficient plus a given effect size. We then fit the TESSERA Poisson-Leroux model as well as a Poisson GLM, an NB GLM, and a GAM to the data and perform a Wald test to see if the contrast is detectable. For comparison, we also evaluate pseudobulk methods DESeq2 and limma-voom. We present curves of the Type I error rate as a function of the p-value threshold across 100 independent simulation trials for each contrast and effect size. Exact control of the Type I error rate is represented by the dotted line. Methods with curves exceeding the dotted line fail to control the Type I error rate, with TESSERA being the only method that achieves error control by remaining consistently below the threshold.

bioRxiv preprint doi: https://doi.org/10.64898/2026.04.27.720955; this version posted April 30, 2026. The copyright holder for this preprint (which was not certified by peer review) is the author/funder, who has granted bioRxiv a license to display the preprint in perpetuity. It is made available under aCC-BY 4.0 International license.

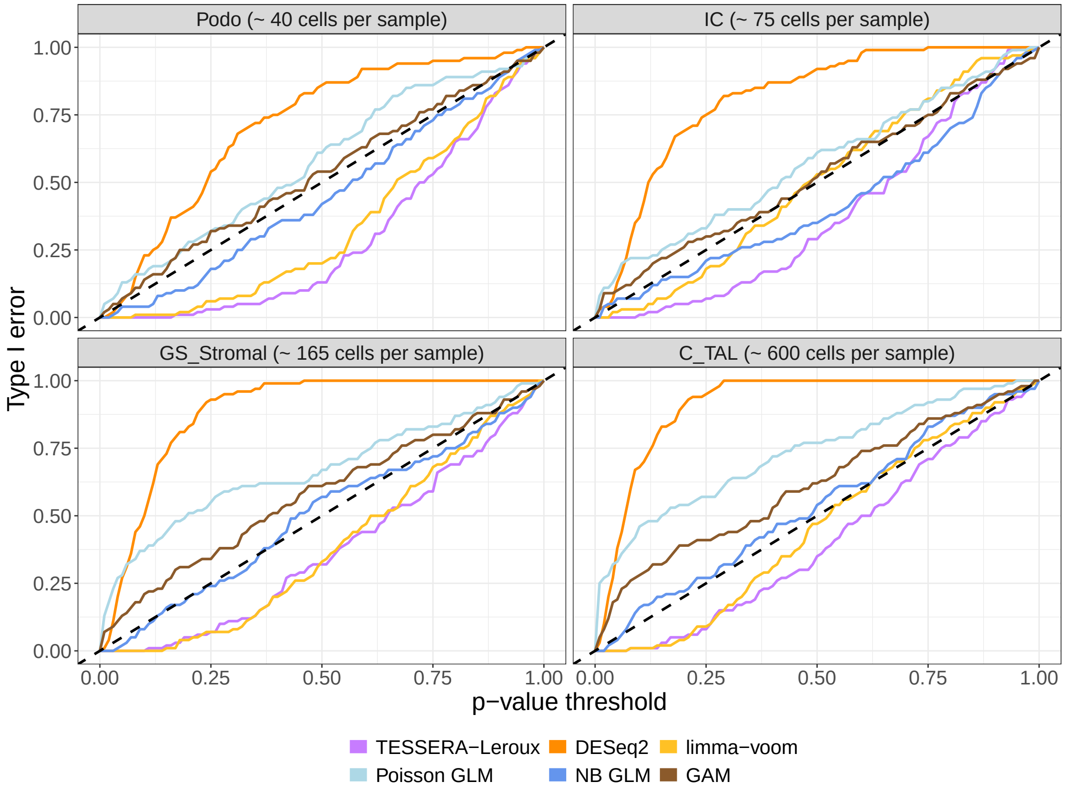

Figure S-19: **Type I error rate for Poisson-spNNGP-generated data.** Synthetic data were generated using a Poisson-spNNGP distribution, with generative parameters estimated from the *IGHG2* gene in a multi-sample real-world kidney dataset (Abedini et al., 2024). For each cell type shown, we modify two entries of the fixed effects vector $\boldsymbol{\beta}$, corresponding to the Control and the DKD conditions to generate a contrast of specified size. We set the control coefficient to be the average of all coefficients and the DKD coefficient to be the control coefficient plus a given effect size. We then fit the `TESSERA` Poisson-Leroux model as well as a Poisson GLM, an NB GLM, and a GAM to the data and perform a Wald test to see if the contrast is detectable. For comparison, we also evaluate pseudobulk methods `DESeq2` and `limma-voom`. We present curves of the Type I error rate as a function of the p-value threshold across 100 independent simulation trials for each contrast and effect size. Exact control of the Type I error rate is represented by the dotted line. Methods with curves exceeding the dotted line fail to control the Type I error rate, with `TESSERA` being the only method that achieves error control by remaining consistently below the threshold.

bioRxiv preprint doi: https://doi.org/10.64898/2026.04.27.720955; this version posted April 30, 2026. The copyright holder for this preprint (which was not certified by peer review) is the author/funder, who has granted bioRxiv a license to display the preprint in perpetuity. It is made available under aCC-BY 4.0 International license.

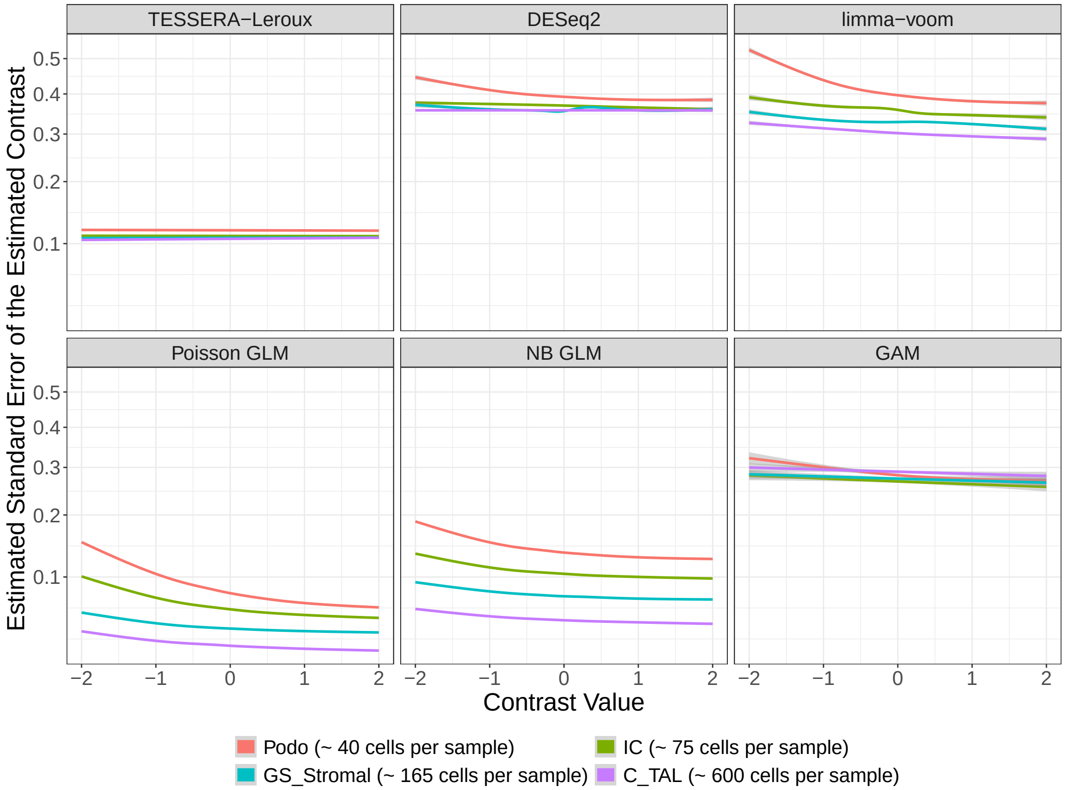

Figure S-20: **Estimated standard error of the estimated contrast for Poisson-Leroux-generated data.** Synthetic data were generated using a Poisson-Leroux distribution, with generative parameters estimated from the *IGHG2* gene in a multi-sample real-world kidney dataset (Abedini et al., 2024). For each cell type shown, we modify two entries of the fixed effects vector β, corresponding to the Control and the DKD conditions to generate a contrast of specified size. We set the control coefficient to be the average of all coefficients and the DKD coefficient to be the control coefficient plus a given effect size. We then fit the TESSERA Poisson-Leroux model as well as a Poisson GLM, an NB GLM, and a GAM to the data and perform a Wald test to see if the contrast is detectable. For comparison, we also evaluate pseudobulk methods DESeq2 and limma−voom. Across 100 independent simulation trials, for each contrast and contrast value, we plot the average estimated standard error of the estimated contrast against the true contrast value.

bioRxiv preprint doi: https://doi.org/10.64898/2026.04.27.720955; this version posted April 30, 2026. The copyright holder for this preprint (which was not certified by peer review) is the author/funder, who has granted bioRxiv a license to display the preprint in perpetuity. It is made available under aCC-BY 4.0 International license.

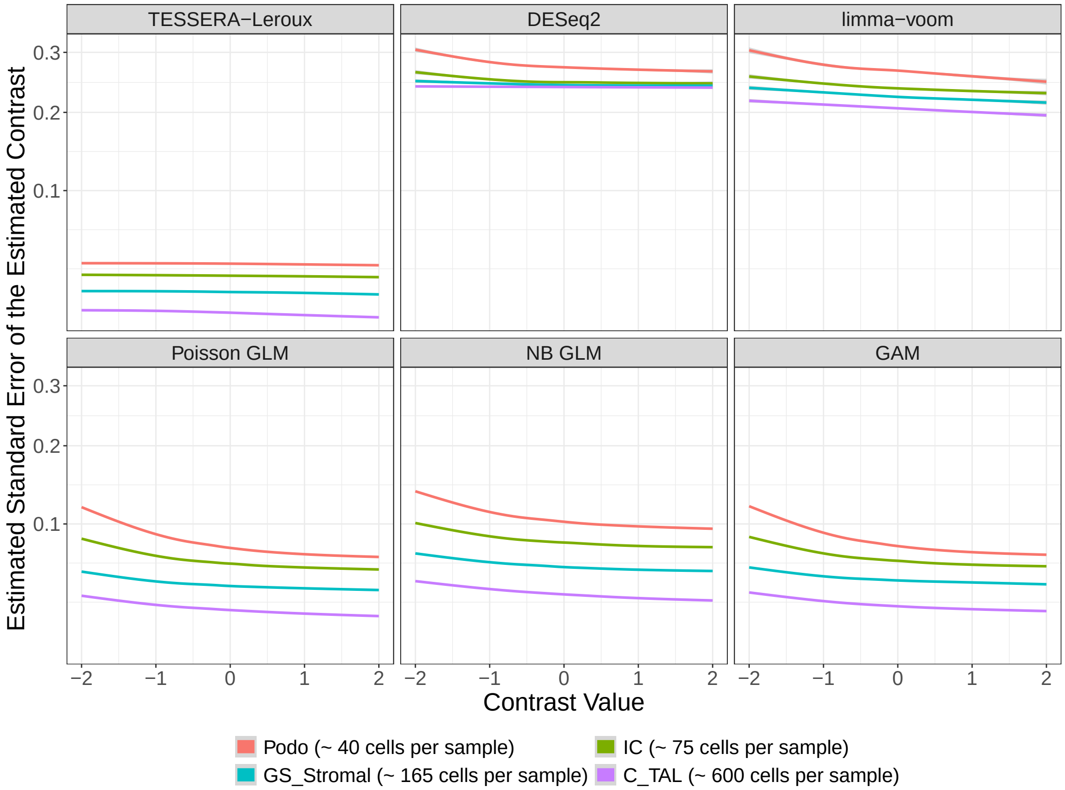

Figure S-21: **Estimated standard error of the estimated contrast for Poisson-spNNGP-generated data.** Synthetic data were generated using a Poisson-spNNGP distribution, with generative parameters estimated from the *IGHG2* gene in a multi-sample real-world kidney dataset (Abedini et al., 2024). For each cell type shown, we modify two entries of the fixed effects vector β, corresponding to the Control and the DKD conditions to generate a contrast of specified size. We set the control coefficient to be the average of all coefficients and the DKD coefficient to be the control coefficient plus a given effect size. We then fit the `TESSERA` Poisson-Leroux model as well as a Poisson GLM, an NB GLM, and a GAM to the data and perform a Wald test to see if the contrast is detectable. For comparison, we also evaluate pseudobulk methods `DESeq2` and `limma-voom`. Across 100 independent simulation trials, for each contrast and contrast value, we plot the average estimated standard error of the estimated contrast against the true contrast value.

bioRxiv preprint doi: https://doi.org/10.64898/2026.04.27.720955; this version posted April 30, 2026. The copyright holder for this preprint (which was not certified by peer review) is the author/funder, who has granted bioRxiv a license to display the preprint in perpetuity. It is made available under aCC-BY 4.0 International license.

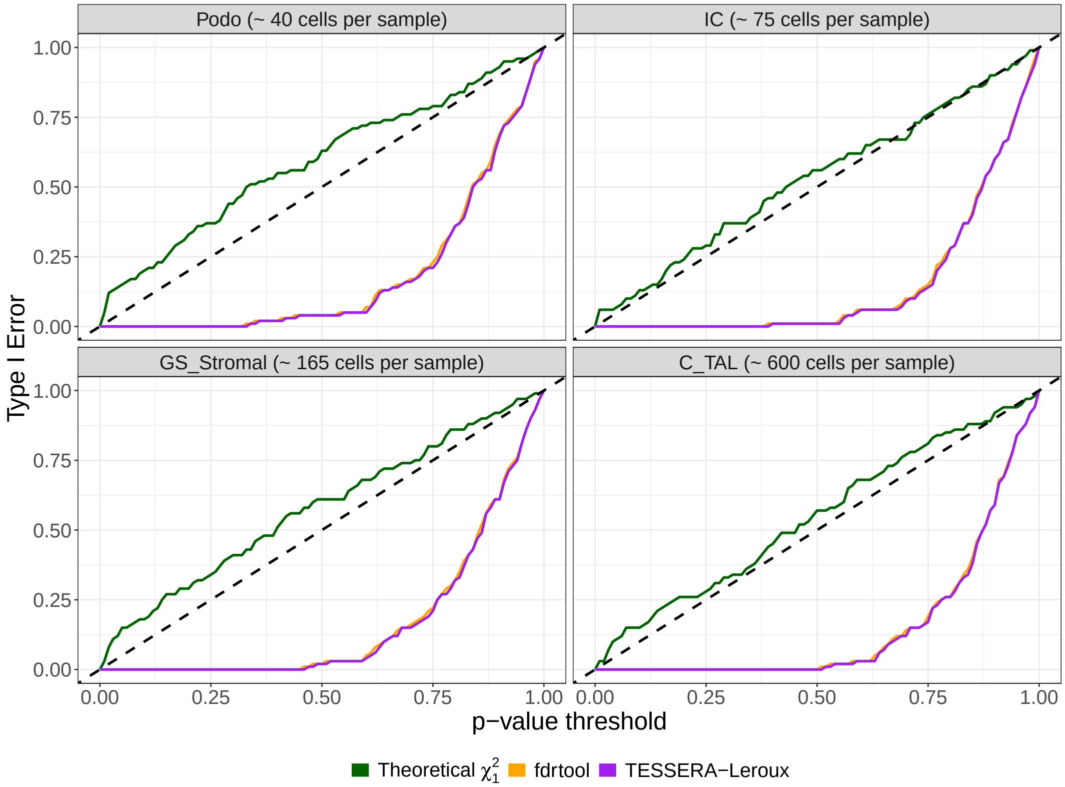

Figure S-22: **Type I error rate of empirical null distribution estimation procedures and theoretical χ^2_1 baseline in Poisson-Leroux-generated data.** Synthetic data were generated using a Poisson-Leroux distribution, with generative parameters estimated from the *IGHG2* gene in a multi-sample real-world kidney dataset (Abedini et al., 2024). For each cell type shown, we modify two entries of the fixed effects vector $\boldsymbol{\beta}$, corresponding to the Control and the DKD conditions to generate a contrast of specified size. We set the control coefficient to be the average of all coefficients and the DKD coefficient to be the control coefficient plus a given effect size. We then fit the TESSERA Poisson-Leroux model and evaluate the detectability of the contrasts by comparing two empirical null distribution estimation procedures, the one used by TESSERA (Section 2.4) and the default fdrtool (Strimmer, 2008) implementation, against a theoretical χ^2_1 baseline. We present curves of the Type I error rate as a function of the p-value threshold across 100 independent simulation trials for each contrast and effect size. Exact control of the Type I error rate is represented by the dotted line. Methods with curves exceeding the dotted line fail to control the Type I error rate, with TESSERA being the only method that achieves error control by remaining consistently below the threshold.

bioRxiv preprint doi: https://doi.org/10.64898/2026.04.27.720955; this version posted April 30, 2026. The copyright holder for this preprint (which was not certified by peer review) is the author/funder, who has granted bioRxiv a license to display the preprint in perpetuity. It is made available under aCC-BY 4.0 International license.

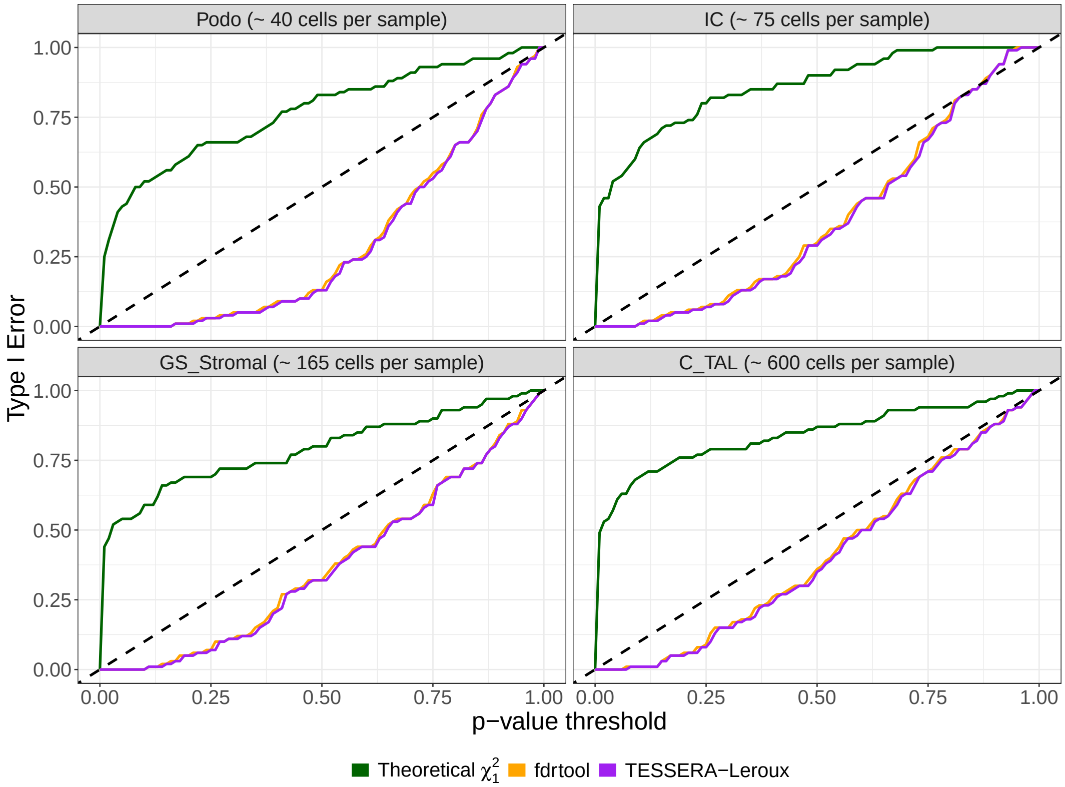

Figure S-23: **Type I error rate of empirical null distribution estimation procedures and theoretical χ_1^2 baseline in Poisson-spNNGP-generated data.** Synthetic data were generated using a Poisson-spNNGP distribution, with generative parameters estimated from the *IGHG2* gene in a multi-sample real-world kidney dataset (Abedini et al., 2024). For each cell type shown, we modify two entries of the fixed effects vector $\boldsymbol{\beta}$, corresponding to the Control and the DKD conditions to generate a contrast of specified size. We set the control coefficient to be the average of all coefficients and the DKD coefficient to be the control coefficient plus a given effect size. We then fit the `TESSERA` Poisson-Leroux model and evaluate the detectability of the contrasts by comparing two empirical null distribution estimation procedures, the one used by `TESSERA` (Section 2.4) and the default `fdrtool` (Strimmer, 2008) implementation, against a theoretical χ_1^2 baseline. We present curves of the Type I error rate as a function of the p-value threshold across 100 independent simulation trials for each contrast and effect size. Exact control of the Type I error rate is represented by the dotted line. Methods with curves exceeding the dotted line fail to control the Type I error rate, with `TESSERA` being the only method that achieves error control by remaining consistently below the threshold.

bioRxiv preprint doi: https://doi.org/10.64898/2026.04.27.720955; this version posted April 30, 2026. The copyright holder for this preprint (which was not certified by peer review) is the author/funder, who has granted bioRxiv a license to display the preprint in perpetuity. It is made available under aCC-BY 4.0 International license.

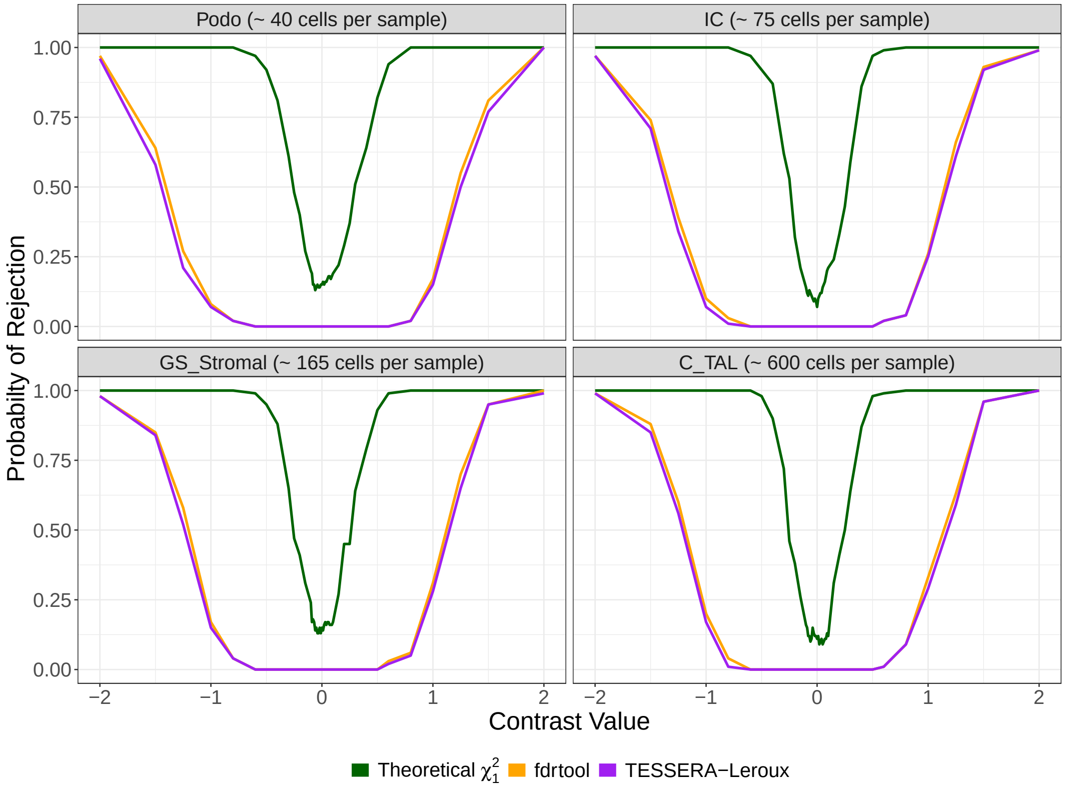

Figure S-24: **Power of empirical null distribution estimation procedures and theoretical χ_1^2 baseline in Poisson-Leroux-generated data.** This figure examines power for detecting DE between conditions within a cell type for one gene. It presents plots of power (y-axis) as a function of the contrast value (x-axis), where power is estimated by the proportion of 100 independent simulation trials in which the null hypothesis of no DE is rejected at nominal Type I error rate of 0.05. At an effect size of 0, this value represents the Type I error rate. Synthetic data were generated using a Poisson-Leroux distribution, with generative parameters estimated from the *IGHG2* gene in a multi-sample real-world kidney dataset (Abedini et al., 2024). For each cell type shown, we modify two entries of the fixed effects vector $\boldsymbol{\beta}$, corresponding to the Control and the DKD conditions, to generate a contrast of specified size. We set the control coefficient to be the average of all coefficients and the DKD coefficient to be the control coefficient plus a given effect size. We then fit the TESSERA Poisson-Leroux model and evaluate the detectability of the contrasts by comparing two empirical null distribution estimation procedures, the one used by TESSERA (Section 2.4) and the default fdrtool (Strimmer, 2008) implementation, against a theoretical χ_1^2 baseline.

bioRxiv preprint doi: https://doi.org/10.64898/2026.04.27.720955; this version posted April 30, 2026. The copyright holder for this preprint (which was not certified by peer review) is the author/funder, who has granted bioRxiv a license to display the preprint in perpetuity. It is made available under aCC-BY 4.0 International license.

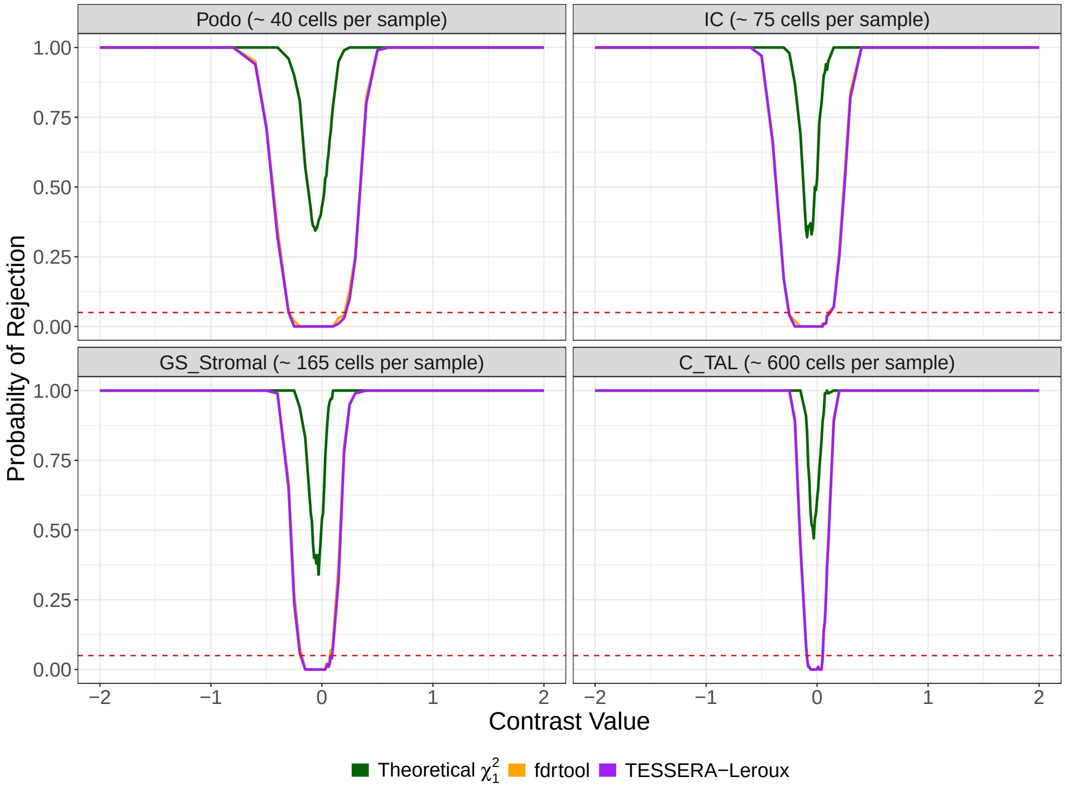

Figure S-25: **Power of empirical null distribution estimation procedures and theoretical χ_1^2 baseline in Poisson-spNNGP-generated data.** This figure examines power for detecting DE between conditions within a cell type for one gene. It presents plots of power (y-axis) as a function of the contrast value (x-axis), where power is estimated by the proportion of 100 independent simulation trials in which the null hypothesis of no DE is rejected at nominal Type I error rate of 0.05. At an effect size of 0, this value represents the Type I error rate. Synthetic data were generated using a Poisson-spNNGP distribution, with generative parameters estimated from the *IGHG2* gene in a multi-sample real-world kidney dataset (Abedini et al., 2024). For each cell type shown, we modify two entries of the fixed effects vector $\boldsymbol{\beta}$, corresponding to the Control and the DKD conditions, to generate a contrast of specified size. We set the control coefficient to be the average of all coefficients and the DKD coefficient to be the control coefficient plus a given effect size. We then fit the `TESSERA` Poisson-Leroux model and evaluate the detectability of the contrasts by comparing two empirical null distribution estimation procedures, the one used by `TESSERA` (Section 2.4) and the default `fdrtool` (Strimmer, 2008) implementation, against a theoretical χ_1^2 baseline.

bioRxiv preprint doi: https://doi.org/10.64898/2026.04.27.720955; this version posted April 30, 2026. The copyright holder for this preprint (which was not certified by peer review) is the author/funder, who has granted bioRxiv a license to display the preprint in perpetuity. It is made available under aCC-BY 4.0 International license.

S7.5. *Real Data: Model Fit.*

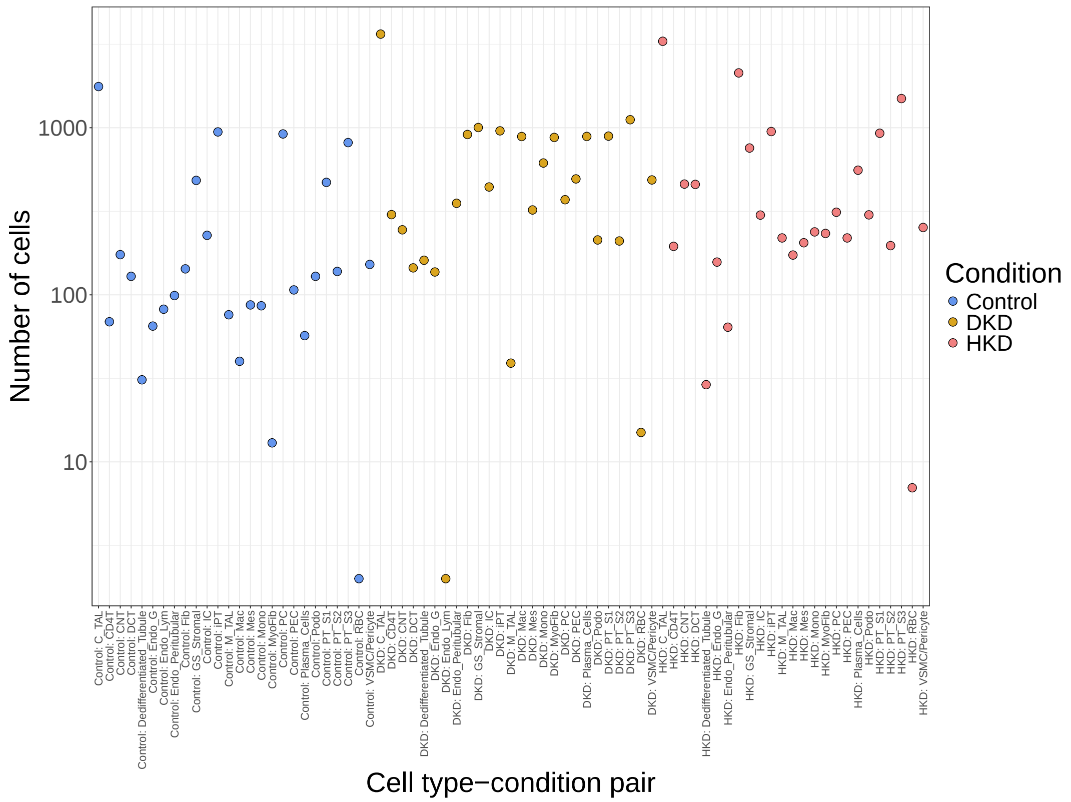

Figure S-26: **Number of expressed cells across cell type-condition pairs.** We plot the total number of cells expressed, i.e., cells with non-zero expression measurements, for each cell type-condition pair (e.g., *C_TAL* cells in DKD samples) in the kidney dataset (Abedini et al., 2024). Each cell type-condition pair corresponds to a column in the design matrix (detailed in Section 4) and the number of expressed cells is the total number of non-zero entries in that column.

bioRxiv preprint doi: https://doi.org/10.64898/2026.04.27.720955; this version posted April 30, 2026. The copyright holder for this preprint (which was not certified by peer review) is the author/funder, who has granted bioRxiv a license to display the preprint in perpetuity. It is made available under aCC-BY 4.0 International license.

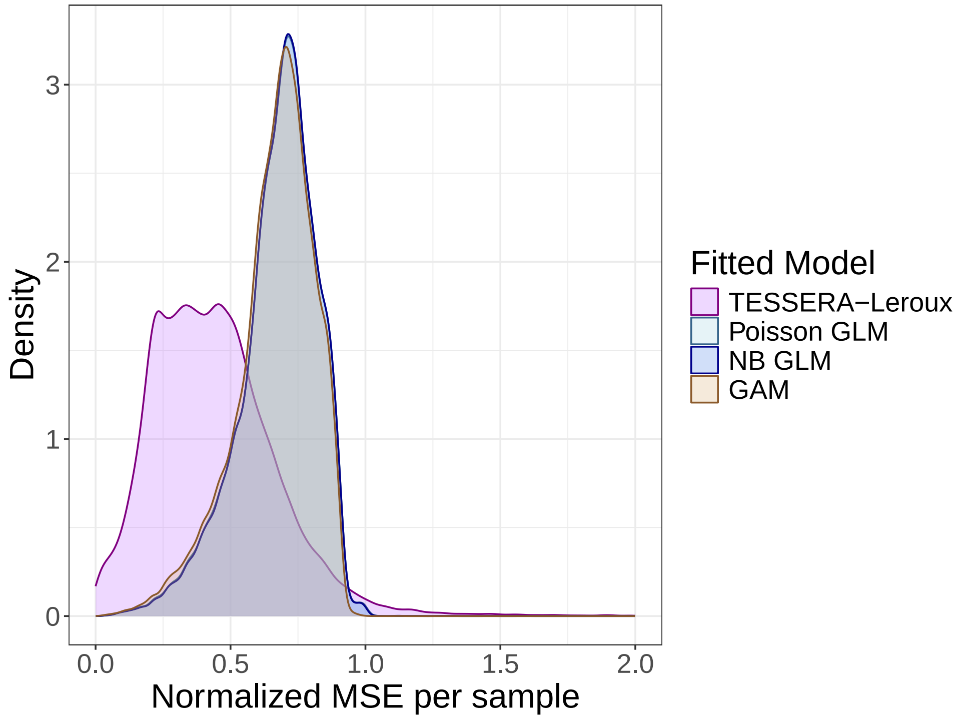

Figure S-27: **Normalized MSE per sample.** For a multi-sample real-world kidney dataset (Abedini et al., 2024), we compare the distribution of the normalized mean squared error (MSE) for TESSERA (fitted with the Poisson-Leroux model) alongside a Poisson GLM, an NB GLM, and a GAM. For each sample-gene pair, the MSE is normalized by the mean of the squared counts: $(\sum_j (Z_{i,j} - \widehat{Z}_{i,j})^2/J_i)/(\sum_j Z_{i,j}^2/J_i)$. The TESSERA Poisson-Leroux model generally has smaller residuals relative to the other methods.

bioRxiv preprint doi: https://doi.org/10.64898/2026.04.27.720955; this version posted April 30, 2026. The copyright holder for this preprint (which was not certified by peer review) is the author/funder, who has granted bioRxiv a license to display the preprint in perpetuity. It is made available under aCC-BY 4.0 International license.

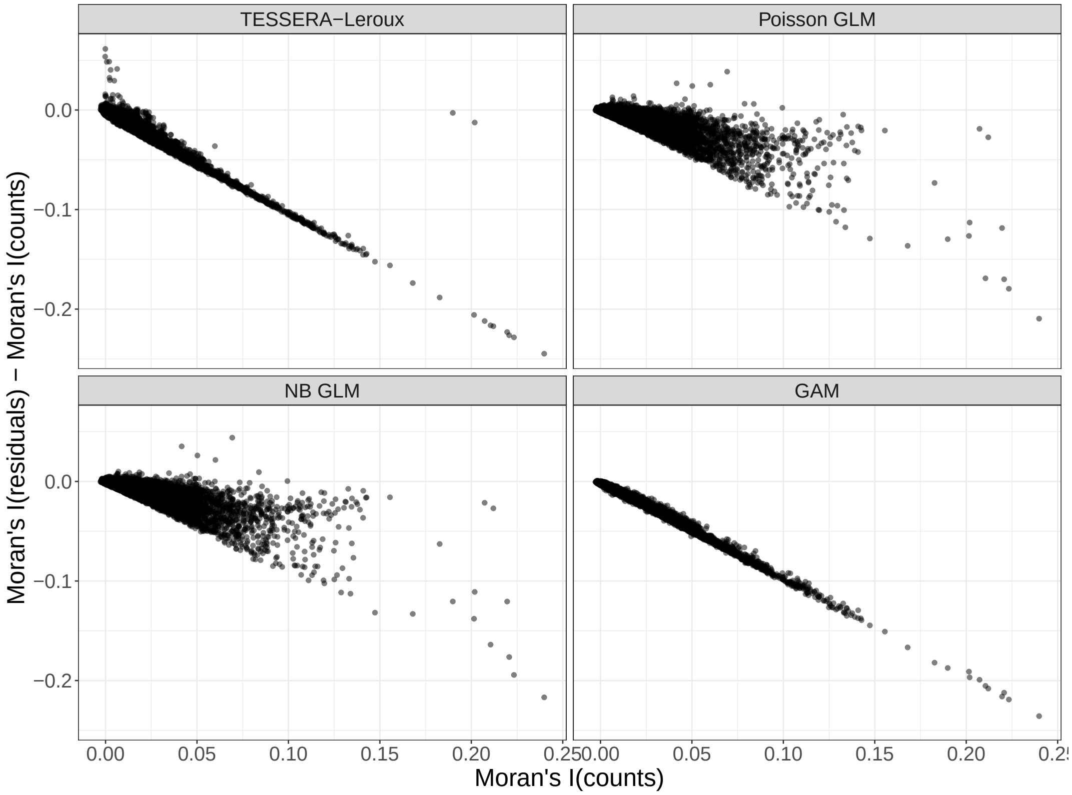

Figure S-28: **Reduction in Moran's I of expression measurements.** For a multi-sample real-world kidney dataset (Abedini et al., 2024), we assess the reduction in autocorrelation resulting from fitting a particular model by computing, for each gene-sample pair, the difference between the Moran's I of the raw counts and that of the residuals. We compare TESSERA (fitted with a Poisson-Leroux model) against a Poisson GLM, an NB GLM, and a GAM. Plots of the differences in Moran's I against the Moran's I of the raw counts indicate that, as expected, the larger the raw count spatial dependencies, the larger the reduction in spatial dependencies.

bioRxiv preprint doi: https://doi.org/10.64898/2026.04.27.720955; this version posted April 30, 2026. The copyright holder for this preprint (which was not certified by peer review) is the author/funder, who has granted bioRxiv a license to display the preprint in perpetuity. It is made available under aCC-BY 4.0 International license.

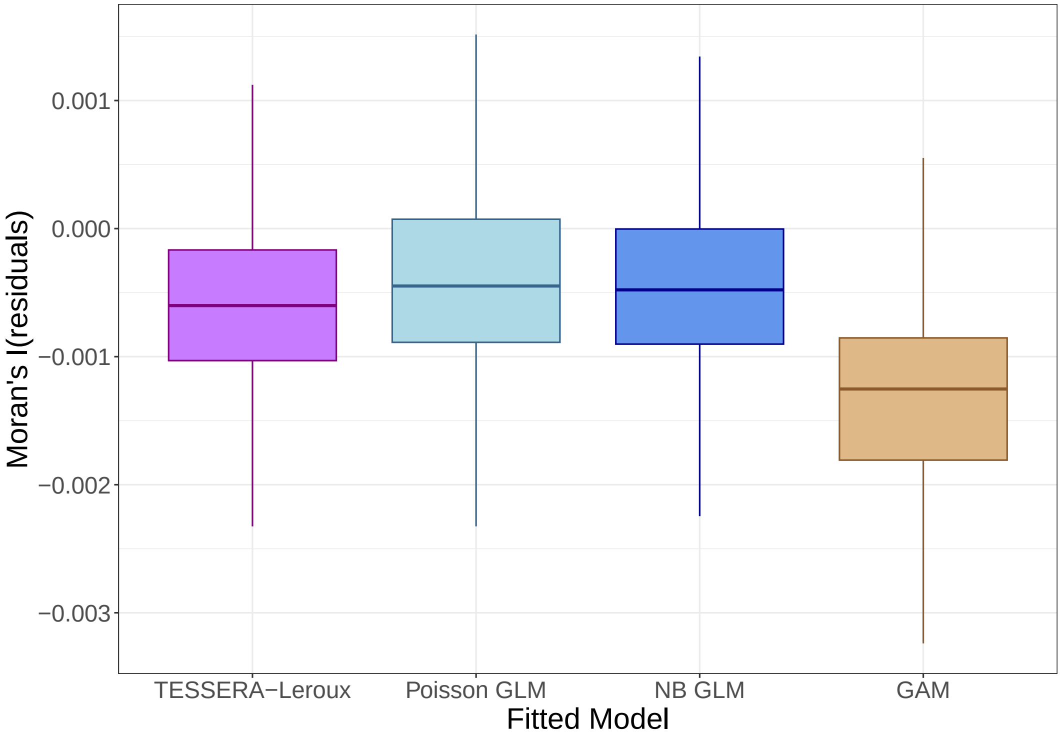

Figure S-29: **Moran's I of residuals for spatially independent synthetic measurements.** For each of 100 independent simulation trials, we generate a synthetic dataset based on the kidney dataset from Abedini et al. (2024) by sampling from a Poisson-Leroux distribution. We use the scale parameters τ^2 derived from fitting the model to the real data and set the spatial correlation parameter γ to zero, yielding a distribution with independent measurements. We then fit the `TESSERA` Poisson-Leroux model as well as a Poisson GLM, an NB GLM, and a GAM to the data. No significant spatial autocorrelation is exhibited, with all methods having Moran's $I \approx 0$.

bioRxiv preprint doi: https://doi.org/10.64898/2026.04.27.720955; this version posted April 30, 2026. The copyright holder for this preprint (which was not certified by peer review) is the author/funder, who has granted bioRxiv a license to display the preprint in perpetuity. It is made available under aCC-BY 4.0 International license.

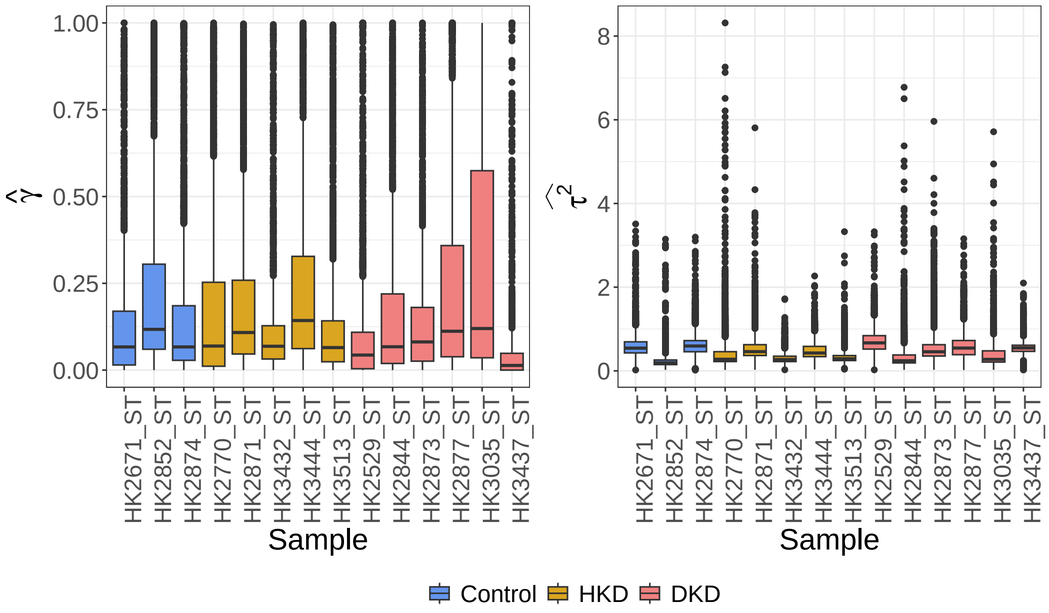

Figure S-30: **Estimated spatial covariance parameters for TESSERA fit with the Poisson-Leroux model.** For a multi-sample real-world kidney dataset (Abedini et al., 2024), we present the gene- and sample-specific estimated spatial covariance parameters γ and τ^2 in boxplots stratified by sample. The boxplots reveal substantial inter-sample heterogeneity, indicating that the magnitude and structure of spatial dependence vary across individual samples. This variation underscores the necessity of a multi-sample method, while the predominantly non-zero estimates $\widehat{\gamma}$ and $\widehat{\tau^2}$ justify the inclusion of spatial random effects.

bioRxiv preprint doi: https://doi.org/10.64898/2026.04.27.720955; this version posted April 30, 2026. The copyright holder for this preprint (which was not certified by peer review) is the author/funder, who has granted bioRxiv a license to display the preprint in perpetuity. It is made available under aCC-BY 4.0 International license.

S7.6. *Real Data: Differential Expression Between Cell Types.*

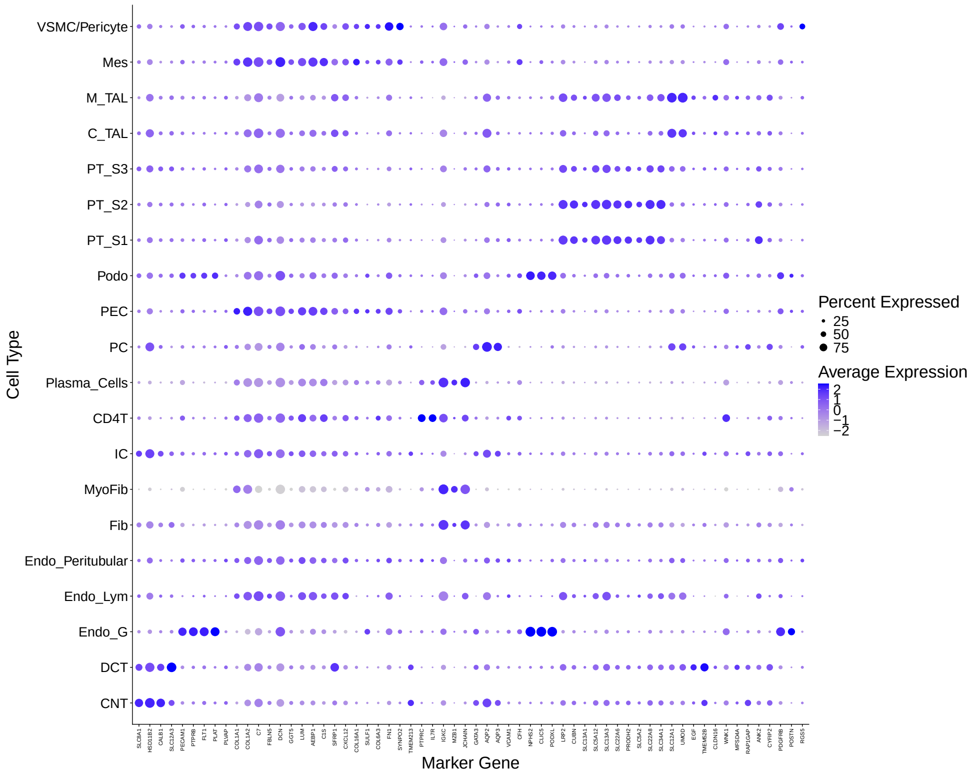

Figure S-31: **Marker gene expression for each cell type.** We visualize the kidney marker genes outlined in Lake et al. (2023, Supp. Table 5) mapped to the same cell types present in the kidney dataset from Abedini et al. (2024). For the marker genes present in the dataset, we plot the percentage of cells expressed and the average expression, scaled to unit variance and zero mean across cell types following library-size normalization and log-transformation.

bioRxiv preprint doi: https://doi.org/10.64898/2026.04.27.720955; this version posted April 30, 2026. The copyright holder for this preprint (which was not certified by peer review) is the author/funder, who has granted bioRxiv a license to display the preprint in perpetuity. It is made available under aCC-BY 4.0 International license.

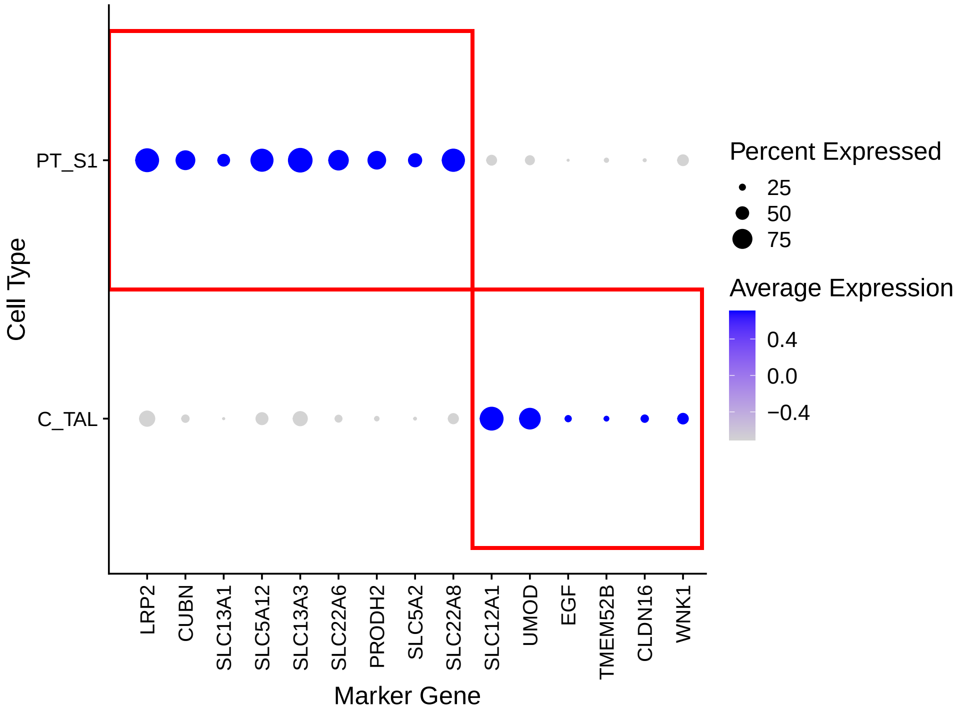

Figure S-32: **Marker gene expression for *PT_S1* and *C_TAL* cell types.** We plot the average expression of marker genes for proximal tubule segment 1 (*PT_S1*) and cortical thick ascending limb (*C_TAL*) cell types in the kidney dataset (Abedini et al., 2024). These genes were selected based on established markers from Lake et al. (2023, Supp. Table 5) that were also present in the dataset. The red boxes indicate the specific marker genes associated with each cell type. We see that the marker genes serve as effective differentiators between the two cell populations.

bioRxiv preprint doi: https://doi.org/10.64898/2026.04.27.720955; this version posted April 30, 2026. The copyright holder for this preprint (which was not certified by peer review) is the author/funder, who has granted bioRxiv a license to display the preprint in perpetuity. It is made available under aCC-BY 4.0 International license.

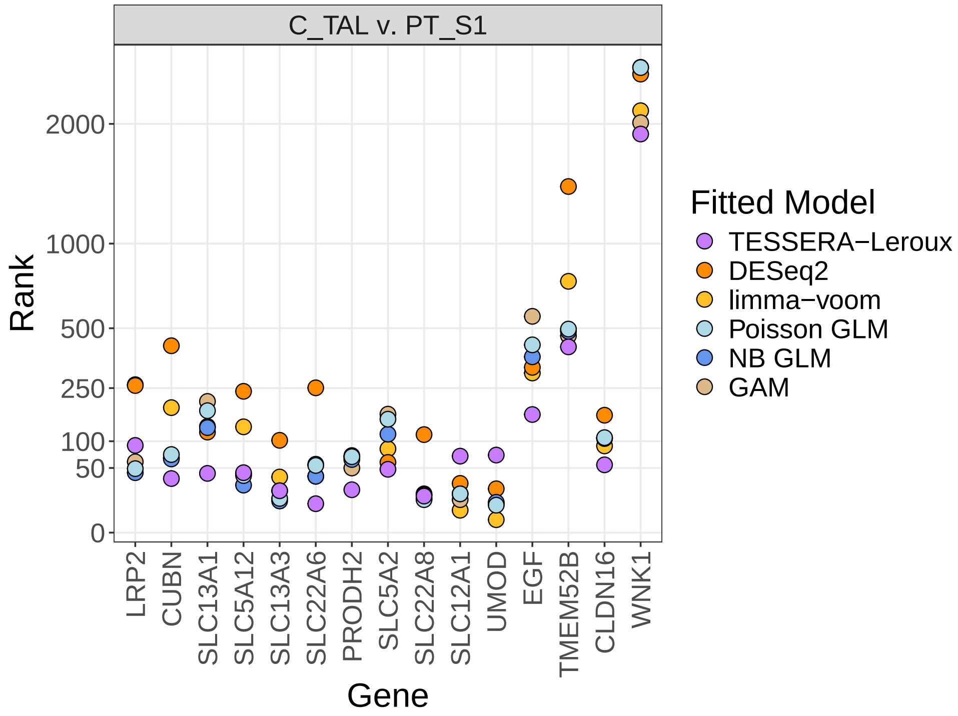

Figure S-33: **Evaluation of marker gene ranks for DE between cell types.** `TESSERA` (fitted with a Poisson-Leroux model), a Poisson GLM, an NB GLM, a GAM, and pseudobulk methods (`DESeq2` and `limma-voom`) are compared in terms of their ability to identify DE genes between the *PT_S1* and *C_TAL* cell types, based on established kidney marker genes for *PT_S1* and *C_TAL* from Lake et al. (2023, Supp. Table 5), which are mapped to the same cell types present in the kidney dataset from Abedini et al. (2024). Genes are ranked according to their test statistics and the plot depicts these rankings specifically for the identified marker genes. A lower numerical rank indicates a larger test statistic, providing stronger evidence of differential expression.

bioRxiv preprint doi: https://doi.org/10.64898/2026.04.27.720955; this version posted April 30, 2026. The copyright holder for this preprint (which was not certified by peer review) is the author/funder, who has granted bioRxiv a license to display the preprint in perpetuity. It is made available under aCC-BY 4.0 International license.

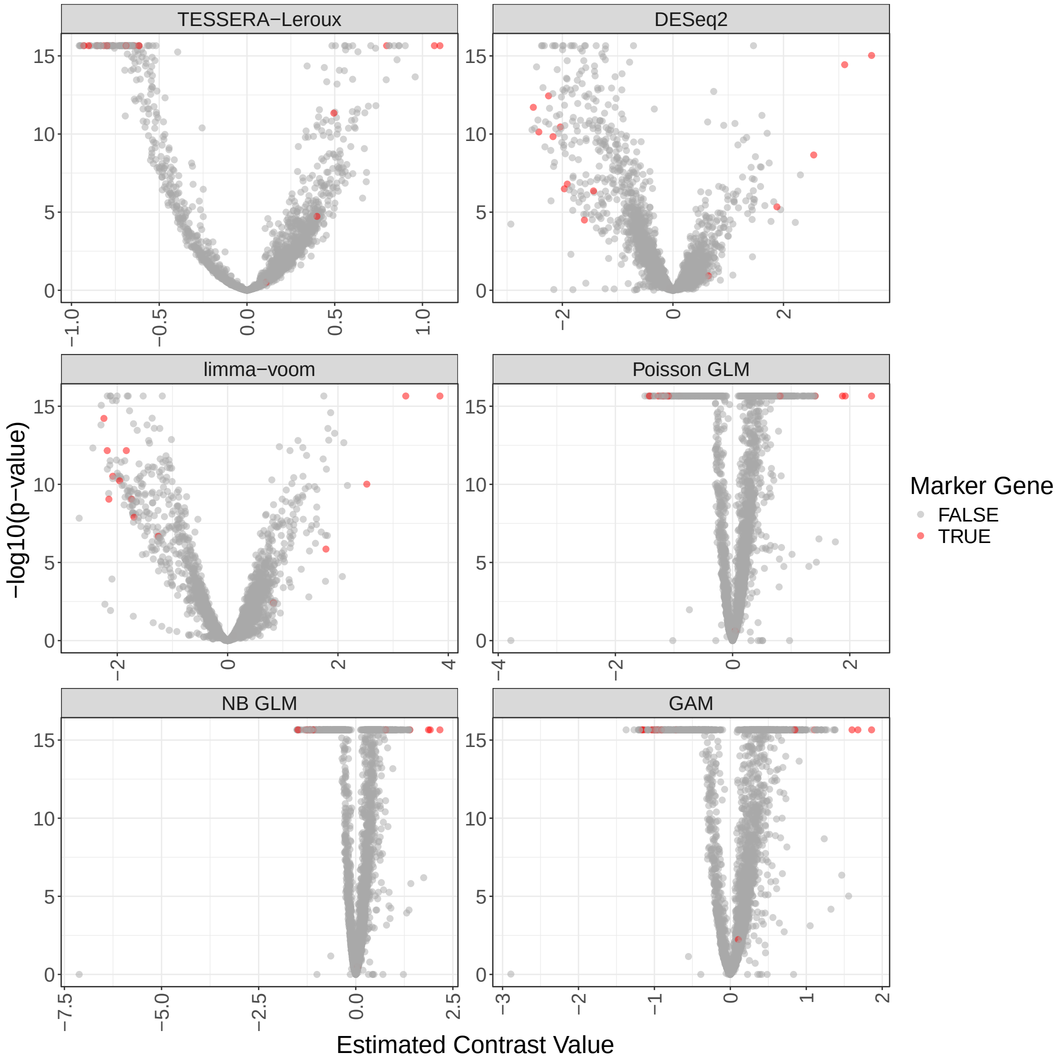

Figure S-34: **Volcano plot for DE between cell types.** TESSERA (fitted with a Poisson-Leroux model), a Poisson GLM, an NB GLM, a GAM, and pseudobulk methods (DESeq2 and limma-voom) are compared in terms of their ability to identify DE genes between the *PT_S1* and *C_TAL* cell types using volcano plots of $-\log_{10}$ p-values against estimated contrast values. Across all methods, established marker genes exhibit larger contrast magnitudes and lower p-values, as expected.

bioRxiv preprint doi: https://doi.org/10.64898/2026.04.27.720955; this version posted April 30, 2026. The copyright holder for this preprint (which was not certified by peer review) is the author/funder, who has granted bioRxiv a license to display the preprint in perpetuity. It is made available under aCC-BY 4.0 International license.

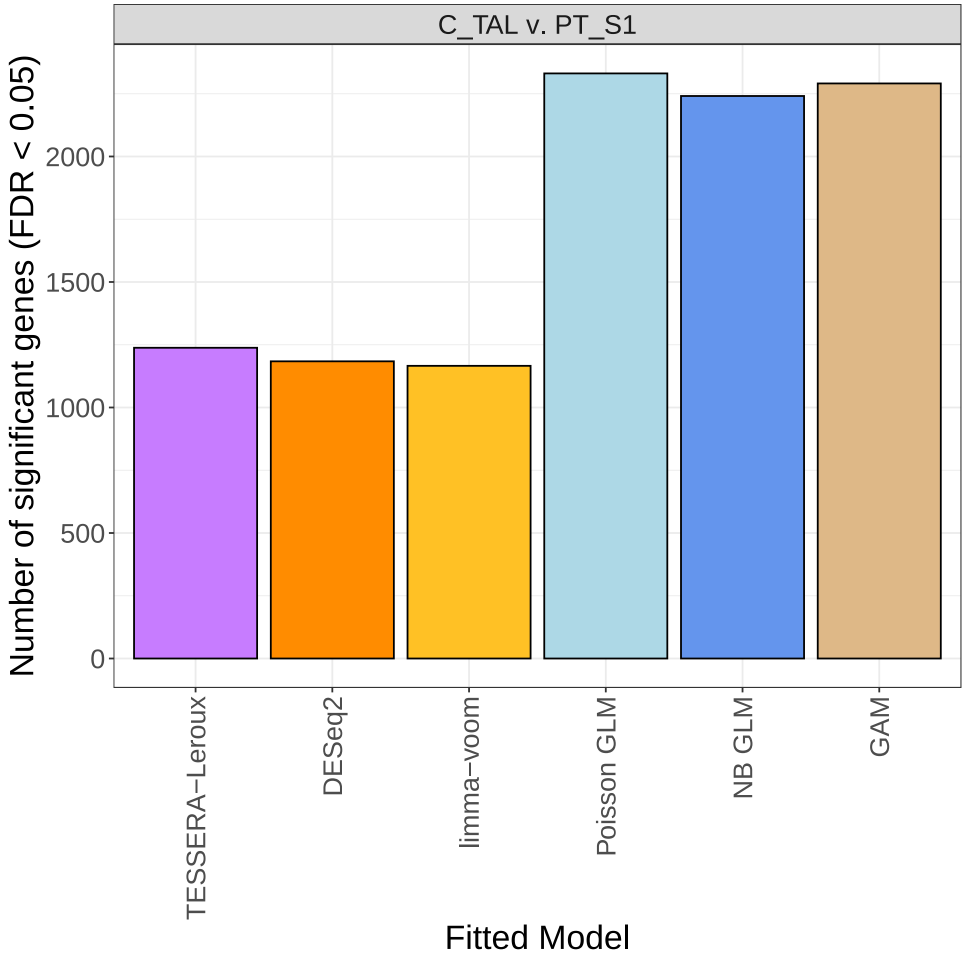

Figure S-35: **Number of genes found to be significantly DE between cell types.** `TESSERA` (fitted with a Poisson-Leroux model), a Poisson GLM, an NB GLM, a GAM, and pseudobulk methods (`DESeq2` and `limma-voom`) are compared in terms of their ability to identify DE genes between the *PT_S1* and *C_TAL* cell types. We plot the total number of genes identified as differentially expressed by each method at a nominal FDR level of 0.05 using Benjamini-Hochberg adjusted p-values.

bioRxiv preprint doi: https://doi.org/10.64898/2026.04.27.720955; this version posted April 30, 2026. The copyright holder for this preprint (which was not certified by peer review) is the author/funder, who has granted bioRxiv a license to display the preprint in perpetuity. It is made available under aCC-BY 4.0 International license.

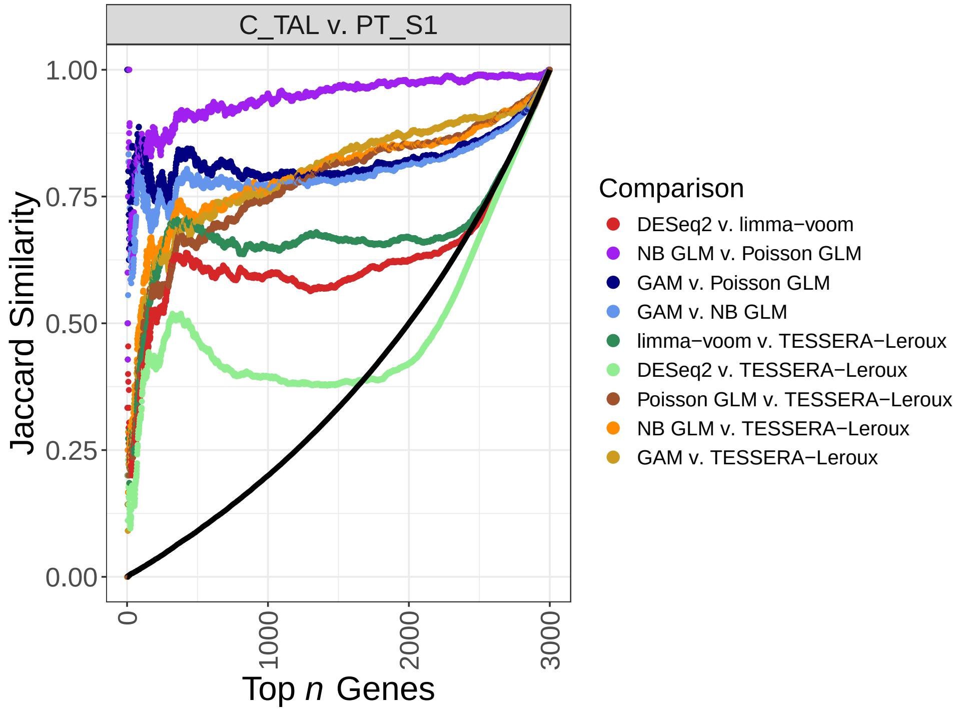

Figure S-36: **Jaccard similarity analysis of gene rankings for DE between cell types.** TESSERA (fitted with a Poisson-Leroux model), a Poisson GLM, an NB GLM, a GAM, and pseudobulk methods (DESeq2 and limma-voom) are compared in terms of their ability to identify DE genes between the *PT_S1* and *C_TAL* cell types. Genes are ranked according to their test statistics and rankings between pairs of methods are compared with the Jaccard similarity (the size of the intersection divided by the size of the union) for each possible set of top n genes, where n ranges from 1 to the total number of 3000 genes. The plot illustrates pairwise comparisons of TESSERA (fitted with a Poisson-Leroux model) against all other methods, the two pseudobulk methods against each other, and all combinations among the single-cell level non-spatial methods (Poisson GLM, NB GLM, and GAM). The black curve represents the null baseline, derived via simulation by calculating the average Jaccard similarity between two randomly permuted gene rankings across 100 trials.

bioRxiv preprint doi: https://doi.org/10.64898/2026.04.27.720955; this version posted April 30, 2026. The copyright holder for this preprint (which was not certified by peer review) is the author/funder, who has granted bioRxiv a license to display the preprint in perpetuity. It is made available under aCC-BY 4.0 International license.

S7.7. *Real Data: Differential Expression Between Conditions, Within Cell Types.*

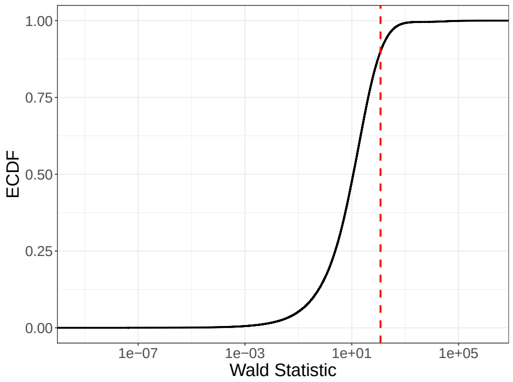

(a) ECDF of Wald test statistics.

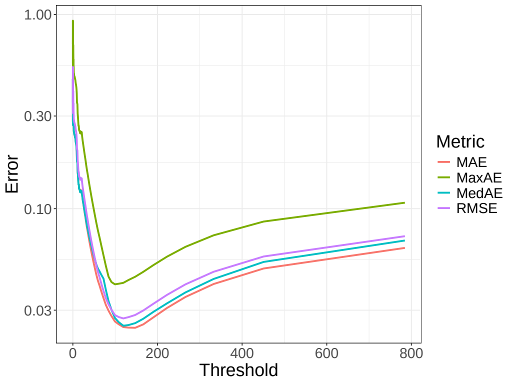

(b) Error measures.

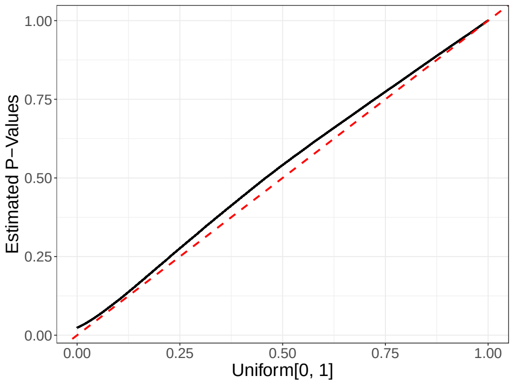

(c) QQ-plot.

Figure S-37: **Behavior of `TESSERA` Wald statistics.** For the kidney dataset (Abedini et al., 2024), we apply `TESSERA` fit with a Poisson-Leroux model to identify genes that are differentially expressed between conditions within the proximal tubule segment 1 (*PT_S1*) cell type. We evaluate the Wald test procedure described in Section 2.4 using several diagnostic plots. Figure S-37a: The empirical cumulative distribution function (ECDF) of the Wald test statistics shows that most statistics remain small, with a rapid increase occurring near the threshold indicated by the red dashed vertical line. Figure S-37b: Our procedure involves selecting a threshold below which statistics are considered to follow the "null" distribution. We measure the error between the resulting p-values and a uniform distribution on $[0, 1]$ as a function of this threshold. While we formally select the threshold by minimizing the MSE, the sharp trough observed across different error measures identifies a natural choice for the threshold; we plot the RMSE alongside these metrics to maintain a consistent scale for visual comparison. Figure S-37c: A quantile-quantile plot (QQ-plot) of the "null" p-values against the Uniform $[0, 1]$ distribution demonstrates a high-quality fit, supported by an L^2-error of 3.4×10^{-4}.

bioRxiv preprint doi: https://doi.org/10.64898/2026.04.27.720955; this version posted April 30, 2026. The copyright holder for this preprint (which was not certified by peer review) is the author/funder, who has granted bioRxiv a license to display the preprint in perpetuity. It is made available under aCC-BY 4.0 International license.

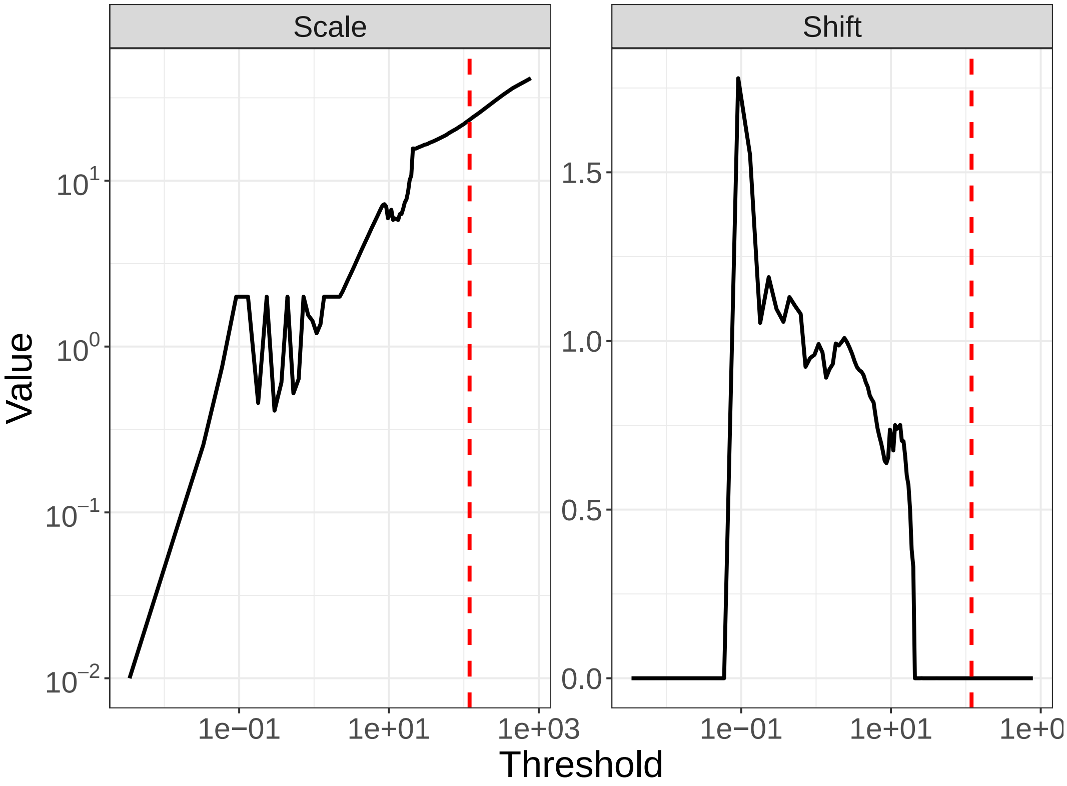

Figure S-38: **Estimation of null distribution parameters for `TESSERA` Wald test statistics.** For the kidney dataset (Abedini et al., 2024), we apply TESSERA fit with a Poisson-Leroux model to identify genes that are differentially expressed between conditions within the proximal tubule segment 1 (*PT_S1*) cell type. We evaluate the Wald test procedure described in Section 2.4 using diagnostic plots of the null distribution parameters. According to our model, the test statistics follow a scaled non-central χ_1^2 distribution under the null hypothesis. We display the estimated parameters of this distribution as a function of the threshold for determining the statistics that come from the "null" distribution. The threshold, selected to minimize the L^2-error (or MSE) relative to a Uniform $[0, 1]$ distribution, is indicated by a red dashed vertical line in both panels. Notably, this threshold occurs just prior to the point where the scale and shift parameters reach a plateau.

bioRxiv preprint doi: https://doi.org/10.64898/2026.04.27.720955; this version posted April 30, 2026. The copyright holder for this preprint (which was not certified by peer review) is the author/funder, who has granted bioRxiv a license to display the preprint in perpetuity. It is made available under aCC-BY 4.0 International license.

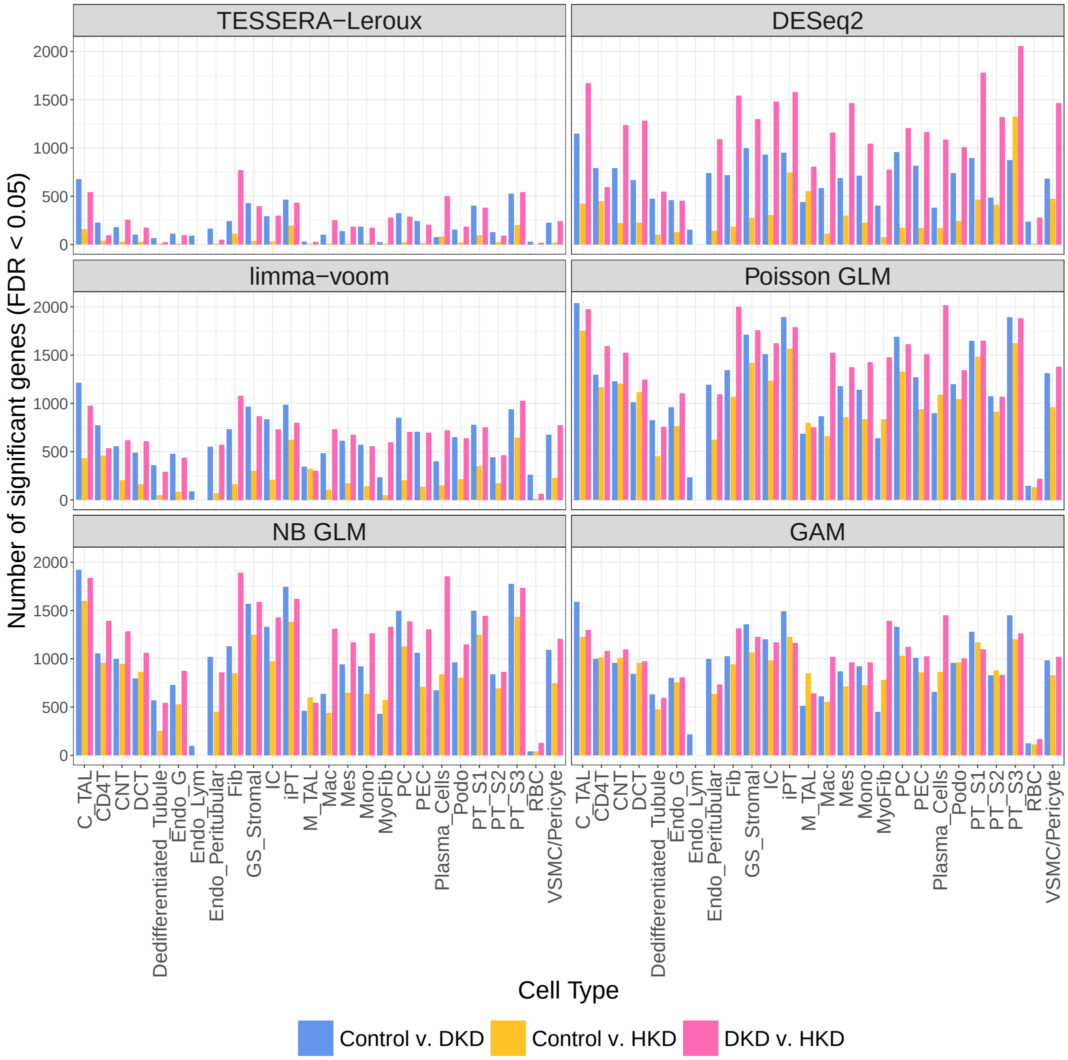

Figure S-39: **Number of differentially expressed genes (faceted by method).** For the kidney dataset (Abedini et al., 2024), we apply several methods to identify genes that are differentially expressed between pairs of conditions within the proximal tubule segment 1 (*PT_S1*) cell type. Significance is determined at a nominal FDR level of 0.05 using Benjamini-Hochberg adjusted p-values. We compare `TESSERA` (fitted with a Poisson-Leroux model) against a Poisson GLM, an NB GLM, a GAM, and pseudobulk methods (`DESeq2` and `limma-voom`). The individual facets of the plot represent the specific methods used to identify these differentially expressed genes. Within each facet, the bars represent the total number of genes identified as differentially expressed for each condition pair across the various cell types.

bioRxiv preprint doi: https://doi.org/10.64898/2026.04.27.720955; this version posted April 30, 2026. The copyright holder for this preprint (which was not certified by peer review) is the author/funder, who has granted bioRxiv a license to display the preprint in perpetuity. It is made available under aCC-BY 4.0 International license.

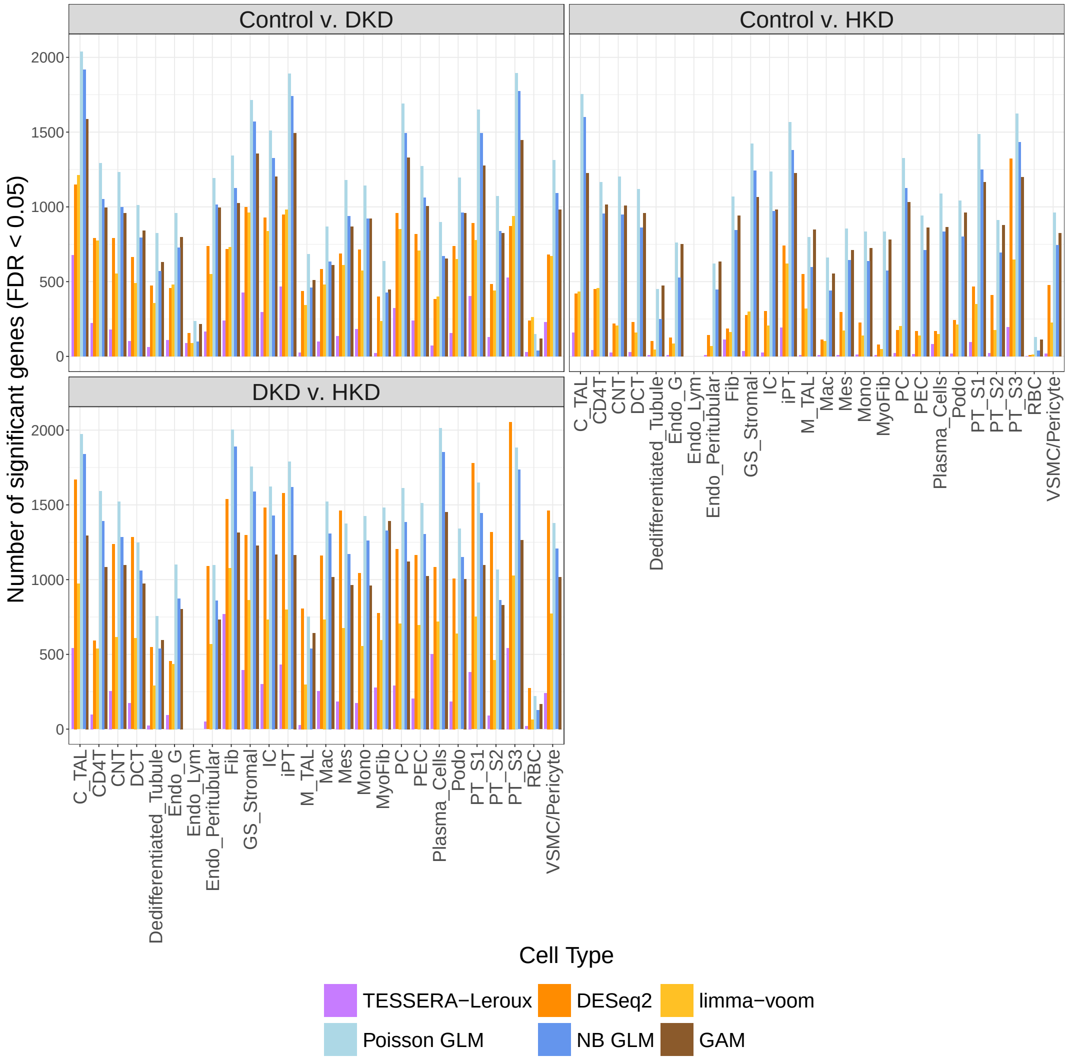

Figure S-40: **Number of differentially expressed genes (faceted by condition pair).** For the kidney dataset (Abedini et al., 2024), we apply several methods to identify genes that are differentially expressed between pairs of conditions within the proximal tubule segment 1 (*PT_S1*) cell type. Significance is determined at a nominal FDR level of 0.05 using Benjamini-Hochberg adjusted p-values. We compare TESSERA (fitted with a Poisson-Leroux model) against a Poisson GLM, an NB GLM, a GAM, and pseudobulk methods (DESeq2 and limma-voom). The individual facets of the plot represent the specific condition pairs being compared. Within each facet, the bars represent the total number of genes identified as differentially expressed by each respective method.

bioRxiv preprint doi: https://doi.org/10.64898/2026.04.27.720955; this version posted April 30, 2026. The copyright holder for this preprint (which was not certified by peer review) is the author/funder, who has granted bioRxiv a license to display the preprint in perpetuity. It is made available under aCC-BY 4.0 International license.

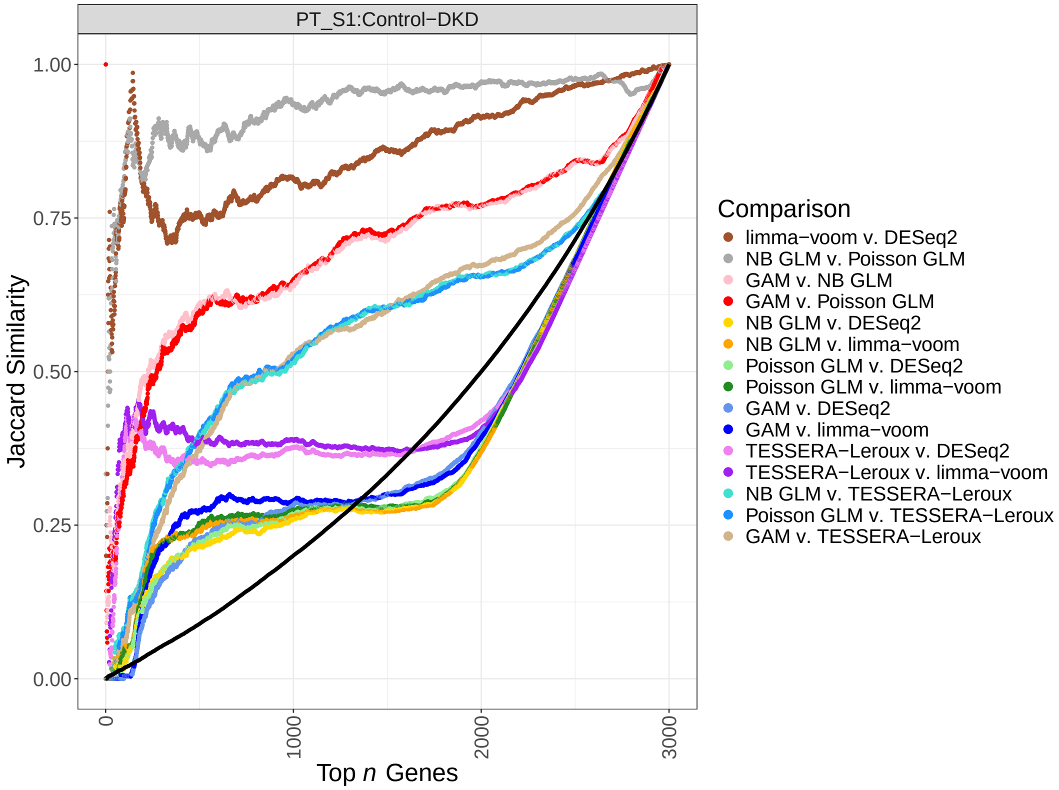

Figure S-41: **Jaccard similarity analysis of gene rankings.** For the kidney dataset (Abedini et al., 2024), we apply several methods to identify genes that are differentially expressed between the Control and DKD conditions within the proximal tubule segment 1 (*PT_S1*) cell type. Genes are ranked by their test statistics calculated from `TESSERA` (fitted with a Poisson-Leroux model), a Poisson GLM, an NB GLM, a GAM, and pseudobulk methods (`DESeq2` and `limma-voom`). We compare the rankings between pairs of methods with the Jaccard similarity (the size of the intersection divided by the size of the union) for each possible set of top n genes, where n ranges from 1 to the total $3,000$ genes. The plot illustrates pairwise comparisons of `TESSERA` (fitted with a Poisson-Leroux model) against all other methods, alongside comparisons between each pair of non-spatial methods, which include both pseudobulk approaches (`limma-voom`, `DESeq2`) and single-cell level models (Poisson GLM, NB GLM, and GAM). The black curve represents the null baseline, derived via simulation by calculating the average Jaccard similarity between two randomly permuted gene rankings across 100 trials.

bioRxiv preprint doi: https://doi.org/10.64898/2026.04.27.720955; this version posted April 30, 2026. The copyright holder for this preprint (which was not certified by peer review) is the author/funder, who has granted bioRxiv a license to display the preprint in perpetuity. It is made available under aCC-BY 4.0 International license.

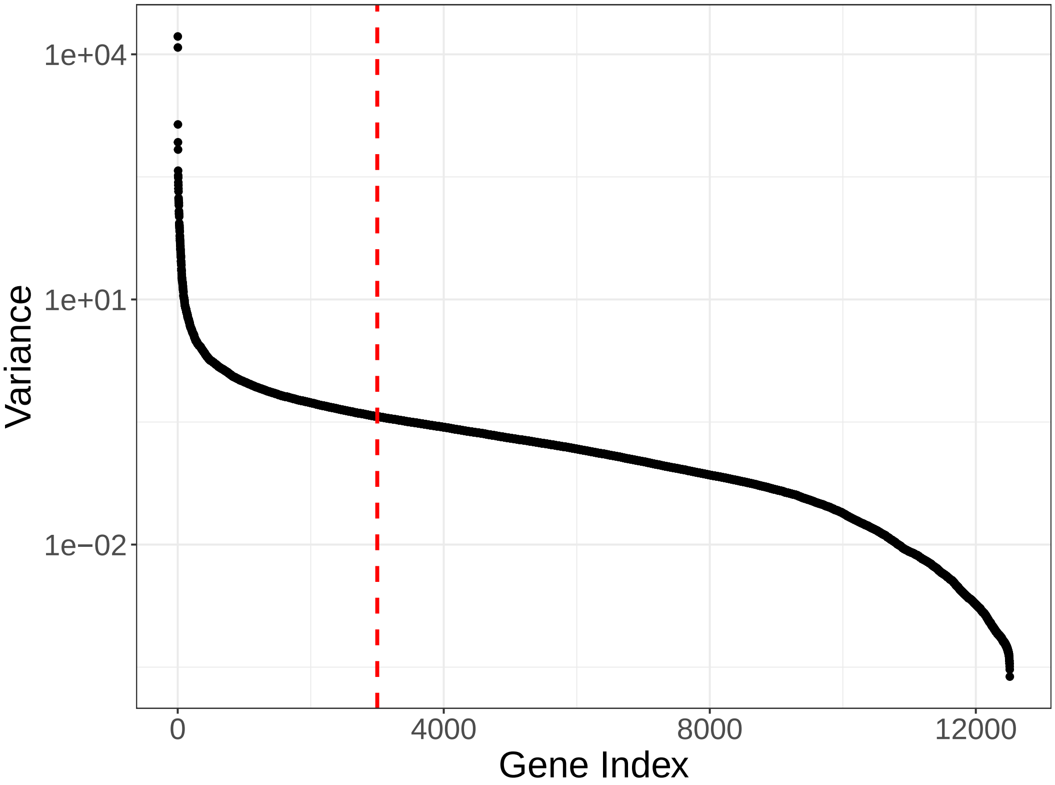

Figure S-42: **Gene selection based on raw count variance.** We plot the sorted variance of raw counts (computed for each gene across all cells) in the kidney dataset (Abedini et al., 2024). The $3,000$ most highly variable genes were selected for downstream analysis as they capture the majority of the variation; beyond this threshold (indicated by the dashed vertical line), the variance profile flattens significantly, indicating a transition toward genes which we also found to have low expression.
